# Transcriptome Profile Analyses of Head Kidney in Roach (*Rutilus rutilus*), Common Bream (*Abramis brama*) and Their Hybrids: Does Infection by Monogenean Parasites in Freshwater Fish Reveal Differences in Fish Vigour among Parental Species and Their Hybrids?

**DOI:** 10.3390/biology12091199

**Published:** 2023-09-01

**Authors:** Andrea Šimková, Kristína Civáňová Křížová, Kristýna Voříšková, Lukáš Vetešník, Vojtěch Bystrý, Martin Demko

**Affiliations:** 1 Department of Botany and Zoology, Faculty of Science, Masaryk University, Kotlářská 2, 611 37 Brno, Czech Republic; kristin@sci.muni.cz (K.C.K.); voriskova@sci.muni.cz (K.V.); 2 Institute of Vertebrate Biology of the Czech Academy of Sciences, Květná 8, 603 65 Brno, Czech Republic; vetesnik@ivb.cz (L.V.); 3 Central European Institute of Technology, Masaryk University, 625 00 Brno, Czech Republic; vojtech.bystry@ceitec.muni.cz (V.B.); martin.demko@ceitec.muni.cz (M.D.)

**Keywords:** Monogenea, *Paradiplozoon homoion*, freshwater fish, hybridization, RNA seq, differential gene expression, hybrid heterosis, hybrid breakdown

## Abstract

**Simple Summary:**

Interspecific hybrids of F1 generations have frequently been characterized by high vigour resulting from heterosis advantage. In contrast, post-F1 generations are expected to express hybrid breakdown, i.e., they suffer from low viability and survival, reproductive abnormalities or sterility and limited ecological performance. Resistance or susceptibility to parasites is one of the measures reflecting hybrid vigour. The present study aimed to analyse the experimental infection of the blood-feeding generalist parasite *Paradiplozoon homoion* (Monogenea) in two target fish species, *Abramis brama* and *Rutilus rutilus*, and their reciprocal F1 hybrids and backcross hybrids, and to reveal potential parasite-induced changes in their transcriptome profiles of head kidney. We hypothesized various effects of hybridization in terms of parasitism in F1 hybrids and backcross hybrids reflected by differential gene expression. The number of differentially expressed genes (DEGs) differed between fish lines with a lower number of DEGs in F1 hybrids and a higher number in backcross hybrids when compared to the parental lines, *A. brama* and *R. rutilus*. Backcross hybrids were more infected than F1 hybrids and parental lines. DEG analyses revealed the role of heme binding, erythrocyte differentiation and immunity-related processes in fish after infection by blood-feeding *P. homoion*. Using GO and KEGG analyses, we revealed the similarity in DEGs between two backcross generations of hybrids. This finding may indicate a potential consequence of hybrid breakdown in backcross generations. Gene expression in less parasitized F1 hybrids is in line of hybrid advantage.

**Abstract:**

Hybrid generations usually face either a heterosis advantage or a breakdown, that can be expressed by the level of parasite infection in hybrid hosts. Hybrids are less infected by parasites than parental species (especially F1 generations) or more infected than parental species (especially post-F1 generations). We performed the experiment with blood-feeding gill parasite *Paradiplozoon homoion* (Monogenea) infecting leuciscid species, *Abramis brama* and *Rutilus rutilus*, their F1 generation and two backcross generations. Backcross generations tended to be more parasitized than parental lines and the F1 generation. The number of differentially expressed genes (DEGs) was lower in F1 hybrids and higher in backcross hybrids when compared to each of the parental lines. The main groups of DEGs were shared among lines; however, *A. brama* and *R. rutilus* differed in some of the top gene ontology (GO) terms. DEG analyses revealed the role of heme binding and erythrocyte differentiation after infection by blood-feeding *P. homoion*. Two backcross generations shared some of the top GO terms, representing mostly downregulated genes associated with *P. homoion* infection. KEGG analysis revealed the importance of disease-associated pathways; the majority of them were shared by two backcross generations. Our study revealed the most pronounced DEGs associated with blood-feeding monogeneans in backcross hybrids, potentially (but not exclusively) explainable by hybrid breakdown. The lower DEGs reported in F1 hybrids being less parasitized than backcross hybrids is in line with the hybrid advantage.

## 1. Introduction

Monogeneans are mainly fish ectoparasites, frequently infecting gills, fins and skin and exhibiting a direct life cycle (i.e., an intermediate host is not involved in their life cycle). Their short generation time can result in exponential population growth. Serious effects on the health condition of wild-living fish have rarely been found; however, the considerable economic impact of monogenean infection on the health of aqua-cultured or farmed fish species has been well documented, i.e., monogeneans reduce fish growth and may cause fish morbidity and mortality [1,2,3,4,5,6].

*Paradiplozoon* (Diplozoidae) are relatively large-bodied monogenean parasites (with a body size ranging in different species from 0.8 to 8.2 mm) and common gill parasites on cyprinoid fish [7]. Among *Paradiplozoon* species, *Paradiplozoon homoion* is a generalist species with the highest range of host species [8,9,10,11]. *Paradiplozoon homoion*, like other diplozoid monogeneans, is representative of blood-feeding ectoparasites that degrade host blood components predominantly intracellularly [12]. Usually, the intensity of infection by *P. homoion* (similarly to other diplozoid species) is low and its prevalence varies depending on the reproduction period of the parasite, the localities of sampling and the host species (e.g., [13,14,15,16]). Adult diplozoids are obligatory blood-feeders, and some diplozoid species, e.g., *Eudiplozoon nipponicum*, are known as important pathogens of farmed cyprinoid fishes [7,8,17,18].

Two fish species, common bream (*Abramis brama*) and roach (*Rutilus rutilis*), both representatives of the family Leuciscidae, are widespread in European freshwaters. The hybridization of these species was previously widely documented [13,19,20,21,22]. Hybrids of *A. brama* and *R. rutilus* in natural habitats primarily result from reciprocal crosses of parental species (i.e., hybrids of F1 generations are present), whilst the presence of post-F1 hybrids is negligible [20,21] indicating that F1 hybrids have reproductive disadvantages, or post-F1 hybrids (backcross or F2) experience breakdown, or at least low survival. Both *A. brama* and *R. rutilus* as well as their F1 hybrids are susceptible to *Paradiplozoon homoion* [13], although *R. rutilus* is documented as the most common host species for *P. homoion* (https://www.nhm.ac.uk/research-curation/scientific-resources/taxonomy-systematics/host-parasites/database, accessed on 30 May 2023).

Generally, hybrids of F1 generations have frequently been characterized by high hybrid vigour, i.e., heterosis advantage, which arises when traits are additive, or due to overdominance resulting in synergic effect of parental alleles on hybrid vigour. F1 hybrids often exhibit superiority over their parents, e.g., greater growth, survival and environmental tolerance or resistance to parasites (for fish that have been documented, see, e.g., [13,23,24,25,26]). For the F1 generation of evolutionarily divergent *A. brama* and *R. rutilus*, hybrid heterosis was revealed through the utilization of a broader trophic spectrum and a higher tolerance to fluctuations in food supply compared to parental species [20,27], and in terms of greater survival at early developmental stages [28], faster growth [29] and lower susceptibility to parasites when compared to parental species [13,16,30]. Studies investigating the metazoan parasite communities in *A. brama*, *R. rutilus* and their F1 hybrids either in natural habitats or under experimental conditions consistently reported lower monogenean abundance in F1 hybrids when compared to parental species, which is in line with hybrid heterosis generally predicted for F1 generations [13,16]. Šimková et al. [30] showed that the diversity of MHC II (major histocompatibility complex) genes, i.e., highly polymorphic genes involved in the adaptive immunity of vertebrates, in F1 hybrids of *A. brama* and *R. rutilus* was intermediate between two leuciscid species, supporting hybrid advantage with respect to coping with parasites (i.e., hybrids with intermediate number of MHC alleles and intermediate positively selected sites potentially reflecting optimal MHC carried fewer parasites than either parental species). However, post-F1 generations of hybrids are expected to express so-called hybrid breakdown, i.e., their biological fitness is reduced as they suffer from low viability, low survival, reproductive abnormalities or sterility and limited ecological performance. The phenomenon of hybrid breakdown results from various genetic incompatibilities [31,32]; hybrids exhibit many disadvantageous traits, often resulting from the disruption of gene expression regulation or the disruption of nuclear–mitochondrial gene interactions [33]. Hybrid breakdown in fish was revealed by several studies (e.g., [25,34,35,36,37]). Regarding *A. brama* and *R. rutilus* hybridization, Stolbunova et al. [38] showed the differences in morphology and suggested the differences in viability (based on asymmetrical ratio of offspring genotypes) between backcrossed hybrids with the mtDNA of *R. rutilus* and mtDNA of *A. brama*, which was interpreted as evidence of varying degrees of cyto-nuclear compatibility of the genomes of *A. brama* and *R. rutilus*.

However, the experimental study of monogenean parasite infection in *A. brama*, *R. rutilus* and their various hybrid lines (including F1 and post-F1 generations) did not support hybrid breakdown (the expectation of high parasite abundance in post-F1 generations) and, in contrast, showed that according to the abundance of host-specific parasites, backcross hybrid generations exhibited similarities with parental species whose genes contributed more to the backcross genotype [16].

Knowledge about the immune response in fish hosts to monogenean infection in terms of changes in gene expression is still underexplored. However, several recent studies have focused on the immune response of fish to monogenean infection, especially to *Gyrodactylus* infection (viviparous Monogenea) and *Dactylogyrus* infection (oviparous Monogenea), underlining the role of mucosal immunity [39], the response through immune gene expression in spleen or head kidney [40,41], or in both skin and/or gills following infection by *Gyrodactylus* [42,43], or the coinfection of viviparous and oviparous representatives of monogeneans, i.e., *Gyrodactylus* and *Cichlidogyrus* [44]. In general, there are not a lot of studies investigating hybrid expression profiles in relation to parasitism, especially such studied are limited in fish, which are, however, organisms of high economic interest. Until now, no studies focusing on the effects of monogenean parasite infection on differential gene expression in the immune organs of pure species and their intergeneric hybrids have been performed, and, at the same time, there are no studies documenting potential changes in immune gene expression after diplozoid infection.

In the present study, we focused on the common generalist monogenean parasite *P. homoion*, the presence of which was previously demonstrated in two target fish species, *A. brama* and *R. rutilus* and their F1 hybrids in natural habitats [13] and in their backcross hybrids under experimental infection [16]. We performed an experimental study to compare the susceptibility of species lines (*A. brama* and *R. rutilus*), F1 hybrids and backcross hybrids using the same infectious dose of the *P. homoion* larval stage (free-living oncomiracidium); we also investigated transcriptome profile changes between infected and control fish of pure breed and hybrid lines. We hypothesized various effects of hybridization in terms of parasitism in F1 hybrids and backcross hybrids reflected by differential gene expression. Specifically, we expected differences in parasite load and transcriptome profile response between F1 hybrids and backcross hybrids, hypothesizing hybrid advantage for F1 hybrids and hybrid breakdown for backcross hybrids.

## 2. Material and Methods

### 2.1. Experimental Fish Lines

Specimens of *A. brama, R. rutilus* and their natural F1 hybrids with *A. brama* mtDNA were collected from the Hamry Reservoir (49.73724 N, 15.91395 E; the Czech Republic). The identification of hybrids was based on meristic traits (i.e., the number of gill rakers, the number of scales in the lateral line and the number of branched rays in the anal fin) and molecular markers (the cytochrome *b* gene and microsatellite loci following Krasnovyd et al. [13]). Artificial breeding of the fish transported to the breeding facility was performed as described in Dedic et al. [16]. Hormonal stimulation of fish for ovulation/spermiation followed Gela et al. [45]; for details, see also Dedic et al. [16]. Oocytes of ovulating females and sperm were sampled according to Gela et al. [45] and Linhart et al. [46]. The artificial breeding was performed for three pairs in each parental combination (see below); the offspring of different parental combinations representing the same fish line was mixed and 5 specimens per each fish line were randomly selected for the experiment.

**Figure 1:**
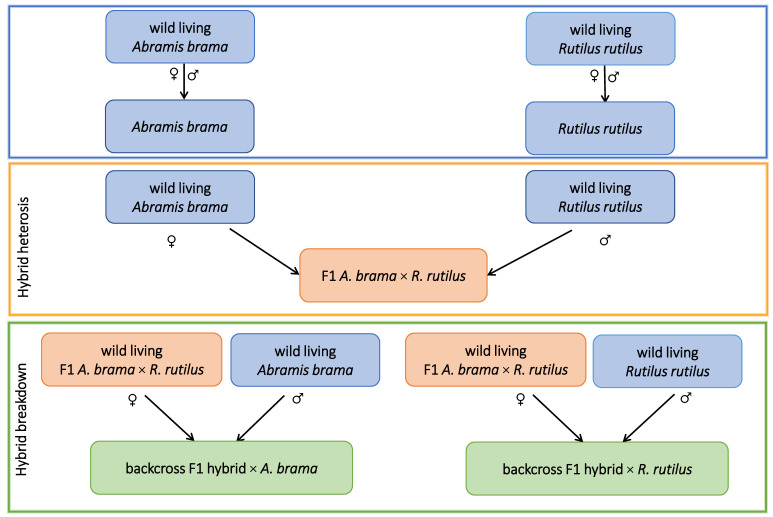
Schema of artificial breeding. Five fish lines used for the experiment are shown (*A. brama*, *R. rutilus*, F1 *A. brama* × *R. rutilus*, backcross of maternal F1 hybrid with *A. brama* mtDNA and paternal *A. brama*, backcross of maternal F1 hybrid with *A. brama* mtDNA and paternal *R. rutilus*). Pure species lines are shown in blue, F1 hybrids are shown in orange, backcross hybrids are shown in green.

The following parental combinations were used for artificial breeding (Figure 1): (1) *A. brama* female and *A. brama* male, (2) *R. rutilus* female and *R. rutilus* male, (3) *A. brama* female and *R. rutilus* male, (4) F1 hybrid female (with *A. brama* mtDNA) and *A. brama* male and (5) F1 hybrid female (with *A. brama* mtDNA) and *R. rutilus* male. Because of the limited number of free-living larval stage, oncomiracidium, used for experimental infection (see below), only F1 generation of hybrids with *A. brama* mtDNA, which is also naturally present in freshwaters, was used in this study. Two years old fish of five lines corresponding to *R. rutilus* line, *A. brama* line, F1 hybrid line with *A. brama* mtDNA (termed F1 *A. brama* × *R. rutilus*) and two backcross lines, i.e., the backcross generation resulting from the crosses of both parents with *A. brama* mtDNA (F1 hybrid female with *A. brama* mtDNA and *A. brama* male, termed backcross of F1 hybrid × *A. brama*) and the backcross generation resulting from the crosses of parents with different mtDNA (F1 hybrid female with *A. brama* mtDNA and *R. rutilus* male, termed backcross of F1 hybrid × *R. rutilus*) were used for the experiment. Standard fish length in millimetres (shown as mean ± SD) for each fish line was measured: *R. rutilus* 79.58 ± 3.68, *A. brama* 82.67 ± 2.99, F1 *A. brama × R. rutilus* 81.42 ± 7.96, backcross of F1 hybrid × *R. rutilus* 80.5 ± 9.84 and backcross of F1 hybrid × *A. brama* 91.17 ± 9.36.

### 2.2. Monogenean Infection

A total of 5 biological replicates (i.e., 5 fish specimens) per fish line were used in the experiment. Each naïve fish specimen was placed individually in a small aquarium (10 L) and acclimatized for one week to experimental room conditions. Temperature of the room was adjusted to 20 °C and aeration in each small aquarium was assured by an air stone roller of size 1 cm.

Eggs of *P. homoion* were collected from the gills of donor fish specimens (donor *Rutilus rutilus* reared under aquarium conditions) and 10–20 eggs were placed into embryo dishes (type 546/40 × 40, VITRUM Rožnov, Czech Republic) filled with tap water that was allowed to stand for 24 h prior to egg transfer. The embryo dishes were then placed into a laboratory incubator at 25 °C. The condition of the eggs was monitored twice daily using a stereomicroscope (Olympus SZX 7, Tokyo, Japan); the water was also changed at the same time. Hatching occurred after 2–3 days. The hatched oncomiracidia were used to infect naïve fish specimens. Twenty-five oncomiracidia specimens were transferred into the aerated and standing tap water in each aquarium in which a single naïve fish specimen was placed. Aeration was stopped for 2 h immediately after oncomiracidia were released into the water to ensure that the oncomiracidia reached the fish (following Pečínková et al. [47], who reported the presence of successful oncomiracidia on fish 2 h from their introduction). Subsequently, aeration was run to ensure fish survival during the experiment. Fish were investigated for the presence of parasites one month after oncomiracidia infection following Pečínková et al. [47] who showed that the development of *P. homoion* from egg to sexually mature adult stage took approximately 33 days. All fish were dissected, i.e., fish were gently sacrificed following law n. 246/1992 of the Czech Republic (Act of the Czech National Council for the Protection of Animals Against Cruelty); then, the gills were removed and using stereomicroscope checked for the presence of *P. homoion* and head kidney of fish was removed using sterile instruments. All parasite specimens of *P. homoion* were counted. Head kidney was collected by fish dissection from individual fish including 5 non-infected control specimens per each fish line and 5 *P. homoion*-infected specimens per fish line. Tissue of each fish was separately submerged in Ambion RNAlater stabilization solution (Thermo Fisher Scientific, Waltham, MA, USA). The tubes with tissues were stored at −80 °C until the isolation of total RNA.

### 2.3. RNA Extraction and Library Preparation

Total RNA was isolated from the head kidney of each fish specimen (25 in total). For extraction, PureLink RNA Mini Kit (Ambion) with Trizol reagent (Thermo Fisher Scientific) and on-column PureLink DNase treatment were used according to the manufacturer’s protocol. Reagent and buffer volumes were adjusted according to the weight of tissue entering the isolation process (15.3 mg on average). The final elution was performed using 100 µL of RNAse-free water in the first step and the primal eluate in the second step. The yield and concentration of RNA isolates were checked using a Qubit^TM^ 4 fluorometer (Invitrogen by Thermo Fisher Scientific) and Qubit RNA HS Assay Kit (Thermo Fisher Scientific). The quality and integrity of RNA were analysed using RNA 6000 Nano Kit on a 2100 Bioanalyzer instrument (Agilent Technologies, Santa Clara, CA, USA).

All RNA isolates were normalized by dilution at a uniform concentration of 20 ng/µL with RNase-free water. They served as templates for DNA library preparation and for the reverse transcription of total RNA into single-stranded cDNA using High Capacity RNA-to-cDNA Kit (Applied Biosystems by Thermo Fisher Scientific) in twice the reaction volume recommended by the manufacturer.

All 25 fish samples (RNA integrity number—RIN > 7) were used for DNA library preparation. We used 500 ng of total RNA for mRNA enrichment using the Poly(A) mRNA Magnetic Isolation Module (New England Biolabs). Subsequently, NEBNext^®^ Ultra™ Directional RNA Library Prep Kit for Illumina^®^ and NEBNext^®^ Multiplex Oligos for Illumina^®^ (Dual Index Primers Set 2, New England Biolabs) were used for library preparation, with 11 PCR cycles utilized for PCR enrichment. RNA fragmentation (13 min at 94 °C) and the size selection conditions (a bead volume of 30 µL and 15 µL for the first and second bead selections, respectively) were further modified in the protocol. The quantification of DNA libraries was performed on a Qubit^TM^ 4 fluorometer (Invitrogen by Thermo Fisher Scientific) using Qubit dsDNA HS Assay Kit, and quality and size control were performed on a 2100 Bioanalyzer with DNA 1000 Kit (Agilent Technologies). Finally, amplicons were pooled in equimolar amounts. The final concentration of each particular library in the pool was 10 nM in the pool. Libraries were sequenced by Macrogen Europe B.V. (Amsterdam, The Netherlands) on an Illumina NovaSeq6000 system (one line on an S4 flow cell) in a paired-end configuration producing 150 bp long reads.

### 2.4. NGS Data Analyses

A quality check of raw paired-end fastq reads was carried out by FastQC [48] and a contamination check against *human*, *mouse*, *yeast*, *E. coli* and other organisms by BioBloom tools [49]. The quality and Illumina adapter trimming of raw reads was performed using Trimmomatic [50]. As a preliminary step, all samples were aligned to the *Carassius auratus* (ASM336829v1-104) and *Cyprinus carpio* (common carp; GCA_000951615.2) genomes using STAR [51]. The mapped reads were deduplicated by Picard’s MarkDuplicates [52]. The quality of mapping was checked using Samtools [53] for overall statistics and Picard for normalized gene coverage, aligned reads strandedness correctness and aligned reads assignment in the reference. The quantification of gene expression was performed by featureCounts [54]. All compatible results and statistics were processed by MultiQC [55]. For more details, see Supplementary File S1.

The poor yield (~30%) of the mapped reads with both genomes (Tables S1 and S2) encouraged us to perform the transcriptome assembly of pure fish lines. First, trimmed reads were mapped against a custom rRNA database (Supplementary File S2) using Bowtie2 [56] and also against the set of overrepresented sequences identified by FastQC using BLAST+ [57]; in addition, all reads mapped to either rRNA or overrepresented sequences were removed to increase the protein-coding transcript yield. Filtered reads were checked using Rcorrector [58] against erroneous k-mers, and all unfixable read-pairs were discarded.

Transcriptome assembly was carried out by Trinity [59], rnaSPAdes [60] and MEGAHIT [61] with multiple k-mer length values. All resulting transcripts were merged together using EvidentialGene’s tr2aacds [62] on the basis of CDS-DNA local alignment identity classification. All transcriptome assemblies (including the merged one) were subjected to various quality control tools: TransRate [63], rnaQUAST [64] and BUSCO [65]. Based on the quality assessment (Tables S5–S8), we decided to proceed with the merged assembly. The annotation step involved TransDecoder [66] and Trinotate [67] as primary tools taking advantage of the following tools and databases: UniProtKB/Swiss-Prot database [68], MEROPS database [69], RefSeq database [70], NCBI Nucleotide database [71], BLAST+ [57], Pfam database [72], HMMER [73] and SignalP [74]. Finally, the abundance of the resulting transcripts was quantified using salmon [75]. All compatible results and statistics were processed by MultiQC. For more details, see Supplementary File S3.

To be able to compare the expression data across lines, we would either need to cross-reference the two obtained transcriptomes or align all samples to the same reference. We tested the second option using the same approach we described earlier. *Abramis brama* uniquely aligned reads mapped to the *R. rutilus* transcriptome ended up only 4% less abundant (on average between samples) than when mapped to the *A. brama* transcriptome and vice versa (Tables S3 and S4). As the *R. rutilus* transcriptome had more predicted genes assembled (Tables S5 and S6), we decided to use it as our main reference and avoid the complicated cross-referencing process.

Differential gene expression analysis between infected and non-infected individuals for each fish line was performed according to gene counts produced by featureCounts and further processed using the Bioconductor package DESeq2 [76]. Genes were considered as differentially expressed only if having the adjusted *p*-value (FDR) smaller than 0.05 and the absolute value of the log2 expression fold-change higher than 1. The DESeq2 tool computes the normalized and shrunk log2 expression fold-change for each gene.

The functional role of significantly differentially expressed genes (i.e., enrichment analysis) was performed according to the KEGG pathway database [77] and the Gene ontology database [78]. For each fish line, the differentially expressed genes were used as the target set, and all of the annotated genes within the transcriptome served as a background for the statistical testing separately for the up- and downregulated significantly differentially expressed genes. The Gene ontology (GO) annotation comes from the semi-automatic annotation made by Trinotate. From the total of 33,767 predicted genes, 12,590 were annotated with GO. KEGG annotation is derived from the KAAS—KEGG Automatic Annotation Server [79] using GHOSTZ [80] on peptide sequences of predicted genes against the set of all fish genes of the KEGG database. From the total of 33,767 predicted genes, 12,618 were annotated with KEGG orthology identifiers. Both functional analyses were done employing in-house scripts using the R packages clusterProfiler [81] for the core of the analysis, data.table [82] for postprocessing and making summary tables and pheatmap [83] and ggplot2 [84] for picturing the results (Supplementary Material with Scripts S1 and S2).

The expression change profile represents the difference in molecular response to *P. homoion* infection for a given gene. We calculated the Spearman correlation between the per-gene expression change profiles and the level of *P. homoion* infection. Biologically relevant genes with the highest correlation were selected for qPCR validation.

### 2.5. Gene Selection and Real-Time Quantitative PCR

Three reference (housekeeping) genes, *RPL8, 18S* and α-tubulin (*A-tub*), were tested to normalize variation in the gene expression. To test their stability in our sample set, 67 representative samples (including at least 5 samples from each line) were subjected to analysis. The amplification was performed under the optimized conditions for qPCR mentioned below. The Reference Gene Selection Tool from Bio-Rad CFX Maestro software (Bio-Rad, Hercules, CA, USA), based on geNorm software principles [85,86] with an algorithm to normalize the Cq of each gene against the Cqs of all reference genes tested across the experimental samples (4), was used. On the basis of their stability, three tested references genes were recognized as follows: *RPL8* (0.66156855; 0.41314167), *A-tub* (0.66156855; 0.41314167) and *18S* (0.73008631; 0.314592522); the average M values and stability values (characterized as (Ln(1/AvgM))) for the tested genes are included in the respective brackets. Finally, two reference genes, *RPL8* and α-tubulin (*A-tub*), were applied to normalize variation in the gene expression in the present study.

A total of 10 biologically relevant genes were selected from transcriptomic outputs and the expressions of 8 of them were validated by real-time quantitative PCR (qPCR). Two genes were excluded because of the amplification of unspecific products. A summary of these genes (including housekeeping genes) is presented in Table 1.

The suitability of primers, their optimal annealing temperatures and amplicon lengths and the specificity of the amplification of all selected genes for representative samples of all fish lines was verified by classical PCR. The PCR reaction mix (10 µL) contained 1× PCR buffer, 1.5 mM MgCl_2_, 10 mM dNTPs, 10 µM forward and reverse primers, 1 U recombinant Taq DNA polymerase (Biogen) and 5 µL of prepared cDNA (see above). PCR was run under the following conditions: initial denaturation at 95 °C/4 min; 30 cycles of 95 °C/30 s, an optimization gradient of 40–65 °C/30 s, 72 °C/45 s; and a final amplification at 72 °C/10 min. At least 5 samples of each line were used for the test.

The following qPCR procedure was applied (also in the testing of amplification efficiency). The reaction mixture (final volume 12 µL) consisted of 6 µL of 2× Power SYBER Green PCR Master mix (Thermo Fisher Scientific), 0.4 µL of each primer, 0.2 µL of ddH_2_O and 5 µL of cDNA template. To test the reaction efficiency and to obtain the standard amplification curve, templates were prepared by means of six serial decimal dilutions of the cDNA of representatives of each fish line. Reactions were run on a CFX96 Real-Time PCR Detection System with a C1000 thermal cycler (Bio-Rad) under the following conditions: 95 °C/10 min; 40 × (95 °C/15 s, 55 °C/30 s, 72 °C/45 s); 95 °C/15 s, 55 °C/1 min, melt curve 55 °C → 95 °C (increment 0.5 °C)/5 s. In each run plate, together with samples run in triplicate, at least one positive control, one negative control and one inter-plate calibrator sample (IPC) in duplex replicates were analysed. For the negative control reaction, RNase/DNase-free water was used instead of the cDNA template. The relative quantification outcomes were analysed by Bio-Rad CFX Manager 3.0 and Bio-Rad CFX Maestro software (Bio-Rad). Unreliable assays, runs and data (110% < E < 90%, −3.58 < slope < −3.10, negative controls c_q_ < 38, R^2^ < 0.98, standard deviation of replicates > 0.3) were excluded from final analyses. The reaction efficiency (E, in %), regression coefficient (R^2^), slope and y-intercept (y-int) were calculated automatically by the abovementioned software. With *RPL8* and *A-tub* as the reference genes, the relative expression value of the differentially expressed target GOI (gene of interest)—the normalized expression—was computed using the ΔΔCq method [87].

**Table 1:** List of reference genes and target genes selected from RNA seq and applied for qPCR validation.

Gen Shortcut	Gene Name	Gene Role (References)	Amplicon (bp)	Forward Primer’s Name	Forward Primer (5′-3′)	Reverse Primer’s Name	Reverse Primer (5′-3′)	Primers’ References
*18S*	18S ribosomal RNA	reference gene (Muduli et al. [88])	140	18S_F	AACGGCTACCACATCCAAGG	18S_R	TCGCCCATGGGTTTAGGATAC	Potrok [89]
*A-tub*	tubulin alpha chain-like	reference gene (Zheng & Sun [90])	157	atub_F	TGCCAACAACTACGCCCG	atub_R	AGAGGTGAAACCAGAGCC	Mo et al. [91]
*RPL8*	ribosomal protein L8	reference gene (Mo et al. [91])	123	RPL8_F	GCAGCAGAAGGCATCCACAC	RPL8_R	CTCCTCCAGACAGCAGACAAT	Mo et al. [91]
*CCND1*	Cyclin D1	target gene (Jirawatnotai et al. [92]; Jiang et al. [93])	208	CCND1_F	AGGAAAATCGTCGCCACATG	CCND1_R	TGCACAACTTCTCTGCTGTT	this study
*ENTH*	ENTH Domain-Containing Protein 1	target gene (Dodd et al. [94])	165	ENTH_F	CAGACCAGCAGTGCATCTTC	ENTH_R	TTTGCTAGTCTGCTCTGCCT	this study
*ING*	Inhibitor of Growth Family	target gene (He et al. [95]); Dantas et al. [96])	182	ING_F	TCGCAGGAAGAAGCACATTG	ING_R	CCATCTCCCGCTCATGTTTC	this study
*HBB*	Haemoglobin Subunit Beta	target gene (Roesner et al. [97])	163	Hemoglobin_F	CGGAAACATGGTTGAGTGGA	Hemoglobin_R	GGCGTTGTAGAGGTTTCCAA	this study
*IL34*	Interleukin 34	target gene (Peixoto et al. [98])	161	IL34_F	GCTTGTGTGCCTCTTGTCC	IL34_R	GGGTGGACGGAGGTATGAAT	this study
*SCAMP5a*	Secretory Carrier-Associated Membrane Protein 5	target gene (Heo et al. [99])	136	Scamp5a_F2	AGGCTCAGGAAGAATGGACC	Scamp5a_R2	TAGTTGTAGGTTGGTGCAGC	this study
*GSN*	Gelsolin	target gene (Azimzadeh & Mohammadisefat [100])	154	GSN_F	GCAAACCTTCCCTCCAGAGA	GSN_R	CCAGGACGGCATAGCATAGA	this study
*ZF-C2H2*	C2H2 zinc finger domain binding	target gene (Yin et al. [101])	172	ZF-C2H2_F	GGTTCAAGCCAGCAAGAATCA	ZF-C2H2_R	GATTTTCCTCRTTCGATTCCAT	this study

## 3. Results

**Figure 2:**
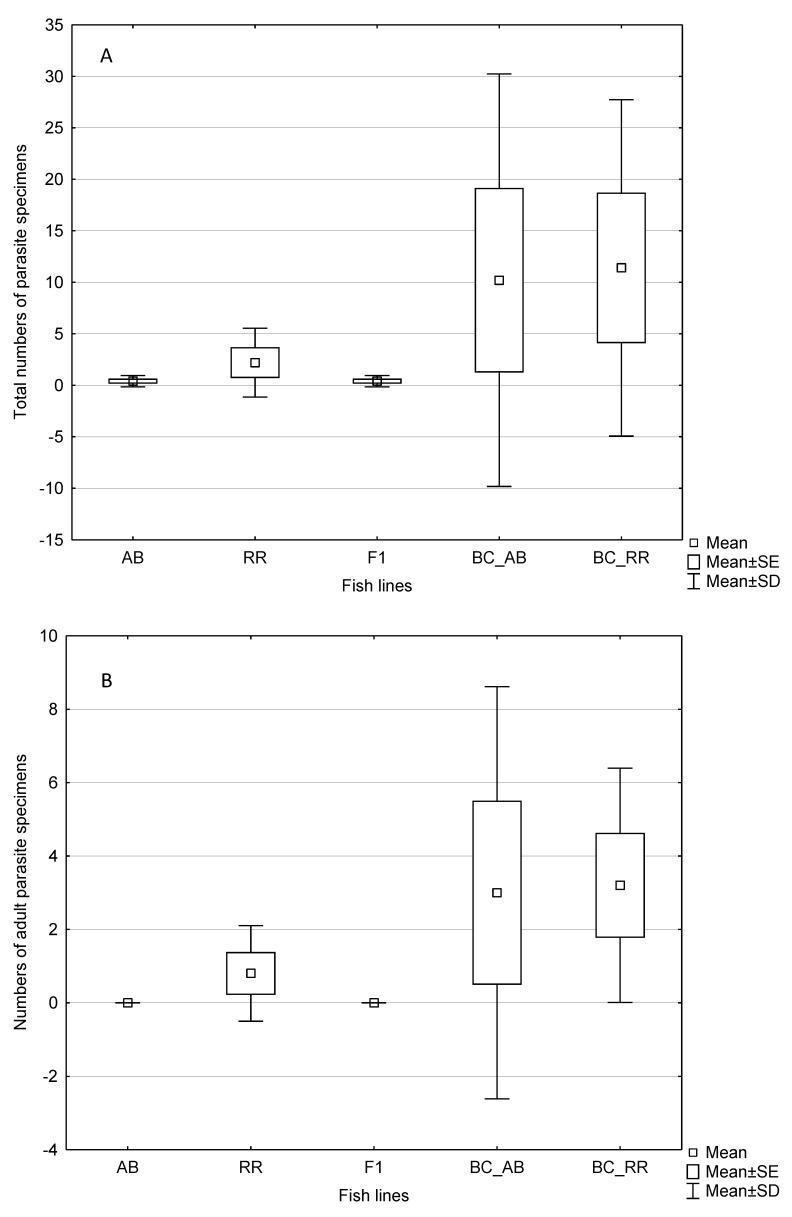
Parasite load in fish lines: (**A**) total numbers of parasite specimens, (**B**) numbers of adult parasite specimens, RR—*Rutilus rutilus*, AB—*Abramis brama*, F1—hybrids of F1 generation (F1 *A. brama* × *R. rutilus*), BC_AB—backcross hybrid generation (F1 hybrid with *A. brama* mtDNA in maternal position and pure *A. brama* in paternal position), BC_RR—backcross hybrid generation (F1 hybrid with *A. brama* mtDNA in maternal position and pure *R. rutilus* in paternal position).

Two developmental stages of *P. homoion* were reported: larval stages termed diporpae, with two or three pairs of attachment clamps, and fused specimens, which originated from the fusion of two diporpae, usually with three pairs or four pairs of clamps (referred to hereafter as the adult form). Diporpae of *P. homoion* were present on specimens of all fish lines; two of five fish specimens were infected in each of the fish lines. The highest numbers of diporpae were found on a single specimen of the backcross generation with paternal *R. rutilus* (24 diporpae) and on a single specimen of the backcross generation with paternal *A. brama* (20 diporpae). No significant difference in abundance of diporpae was found between specimens of pure lines and F1 hybrids or backcross hybrids (Mann–Whitney (MW) test, *p* > 0.05). Adult forms (i.e., the fused form) of *P. homoion* were found only on specimens of *R. rutilus* (two of five specimens), specimens of the backcross generation with paternal *R. rutilus* (all five specimens) and specimens of the backcross generation with paternal *A. brama* (three of five specimens). No specimen of *A. brama* or the F1 generation of hybrids were infected by the fused form of *P. homoion* (Figure 2). Significant difference in abundance of adult *P. homoion* was found between specimens of pure lines and backcross hybrids (MW test, *p* = 0.023), with backcross hybrids being more infected than specimens of pure parental lines. The same difference was found when considering total *P. homoion* abundance, i.e., considering both diporpae and adult counts (MW test, *p* = 0.017). Significant difference in abundance of adult *P. homoion* was found between F1 hybrids and backcross hybrids (MW test, *p* = 0.017) with backcross hybrids being more infected than F1 hybrids. The same difference was found when considering total *P. homoion* abundance (MW test, *p* = 0.014). No significant difference in abundance of adult *P. homoion* or total abundance was found between specimens of pure parental lines and F1 hybrids (MW test, *p* > 0.05). The proportions of infected and non-infected specimens in pure parental lines and backcross hybrids tended to be different (Chi-square test, *p* = 0.051). The proportions of infected and non-infected specimens in F1 hybrids and backcross hybrids were different (Chi-square test, *p* = 0.039). At the time of fish dissection, the numbers of parasites varied among and within fish lines (Figure 2). Within fish lines, a single diporpae as the maximum intensity of infection was found on individual *A. brama* or F1 hybrids, whilst the number of parasites per infected fish specimen (calculating each fused parasite as two specimens) within the remaining lines, i.e., *R. rutilus*, the backcross generation with paternal *R. rutilus* and the backcross generation with paternal *A. brama*, ranged from 1 to 46 per individual fish. Maximum intensity of infection of *P. homoion* was similar in two backcross generations (46 in backcross generation with paternal *A. brama* and 40 in backcross generation with paternal *R. rutilus*).

**Figure 3:**
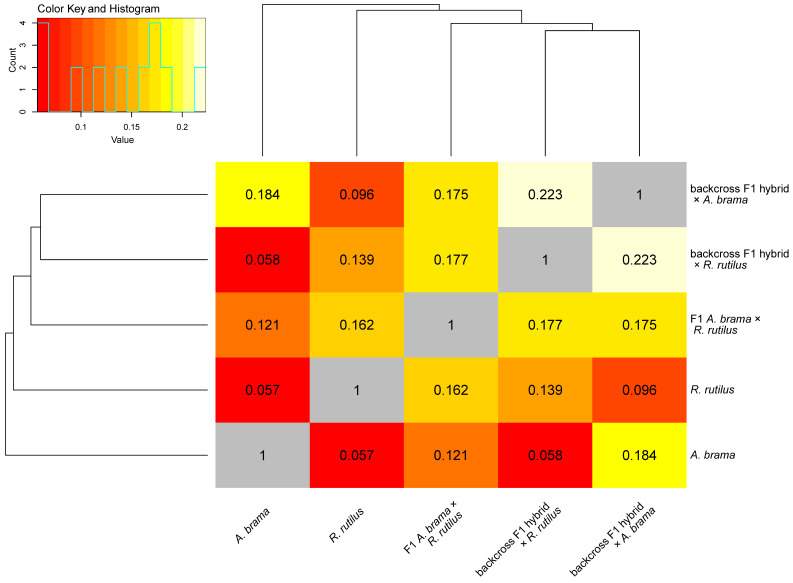
Heatmap of the correlations of gene expression profiles between fish lines. The similarity between fish lines is shown.

Based on correlations of gene expression profiles, the similarities between pairs of fish lines are presented in Figure 3. Despite the overall trend of similarities between the fish lines, the highest similarity was found between two backcross hybrid generations, followed by the similarity between *A. brama* and the backcross generation resulting from the crossing of F1 hybrid × *A. brama* (both with *A. brama* mtDNA). F1 hybrids were more similar to backcross generations of hybrids than to each of the parental species.

**Figure 4:**
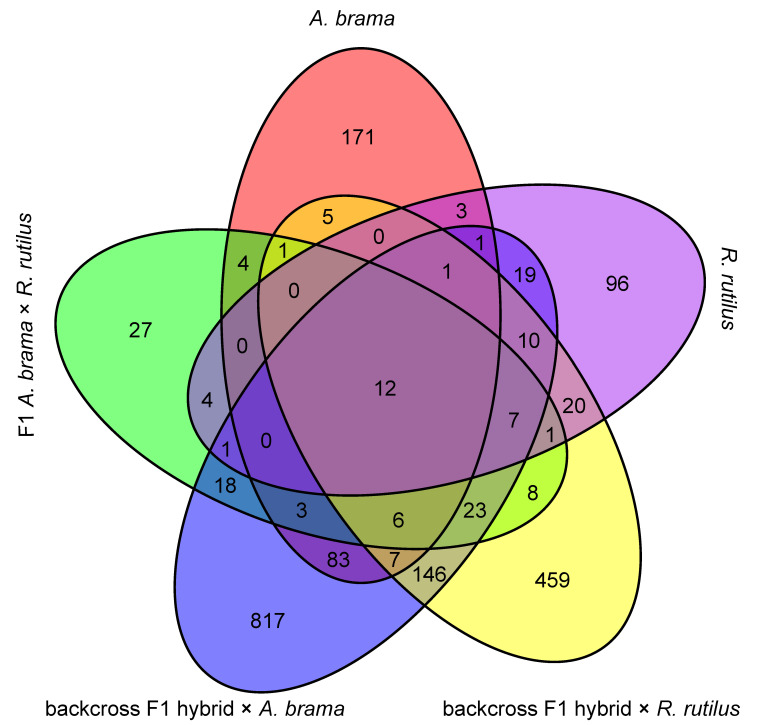
Venn diagram of DEG overlap between fish lines.

The overlap in differentially expressed genes between fish lines is expressed in a Venn diagram (Figure 4), which shows the highest number of DEGs shared between two backcross generations.

**Figure 5:**
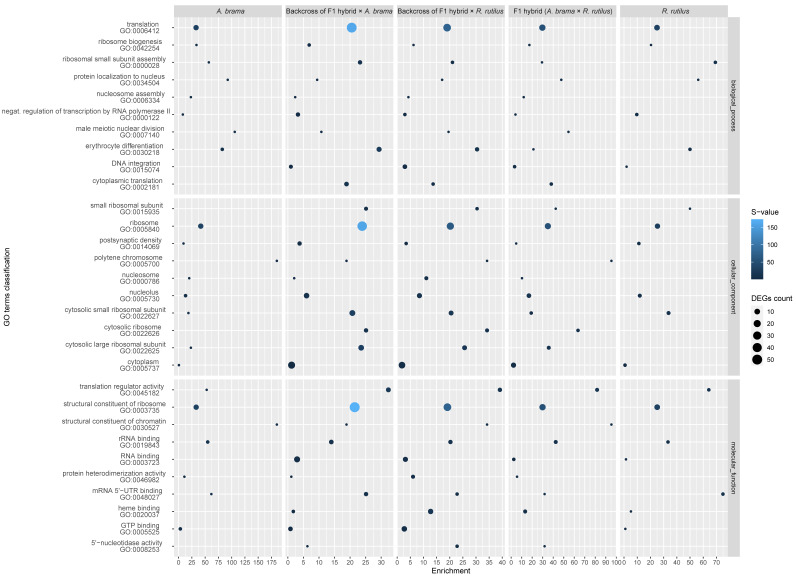
Enrichment score of GO enrichment analysis based on downregulated genes for infected vs. control comparison. The plot shows the top 10 GO terms among all fish lines for three main GO functional categories individually. The enrichment score is computed as ((number of DE genes with particular GO annotation divided by number of all DE genes)/(number of all genes with particular GO annotation divided by number of all genes)). DE genes filtered by FDR ≤ 0.05 and log2FC ≤ −1.

**Figure 6:**
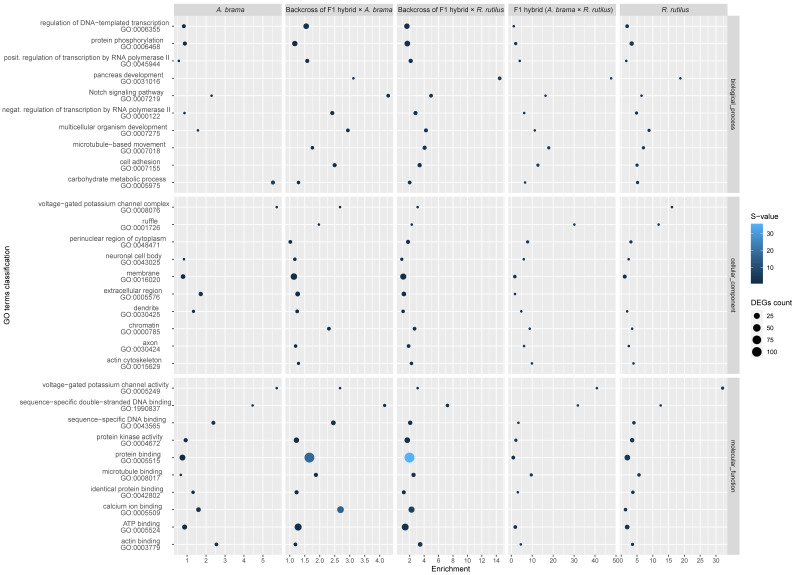
Enrichment score of GO enrichment analysis based on upregulated genes only for infected vs. control comparison. The plot shows the top 10 GO terms among all fish lines for three main GO functional categories individually. The enrichment score is computed as ((number of DE genes with particular GO annotation divided by number of all DE genes)/(number of all genes with particular GO annotation divided by number of all genes)). DE genes filtered by FDR ≤ 0.05 and log2FC ≥ 1.

**Figure 7:**
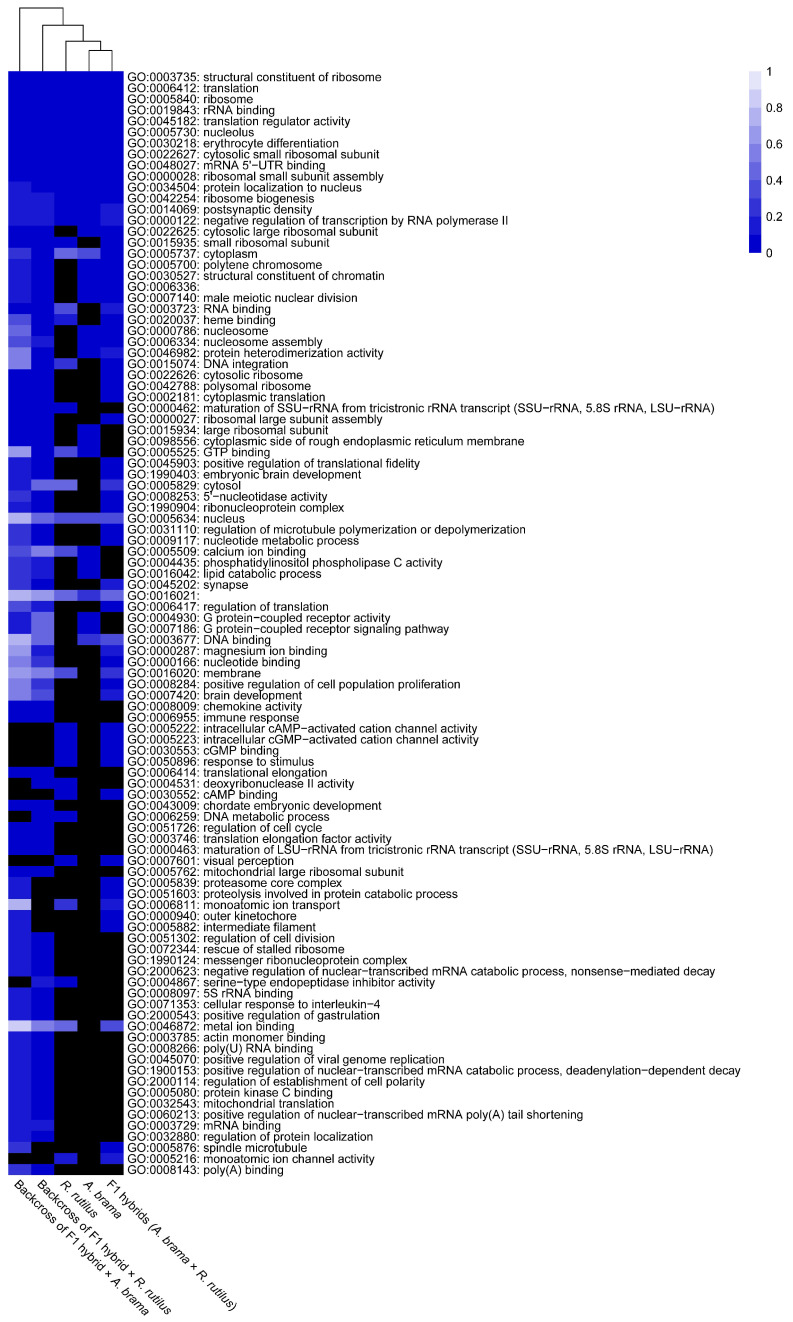
Heatmap of the statistical significance of GO enrichment analysis based on downregulated genes only for infected vs. control comparison. DE genes filtered by FDR ≤ 0.05 and log2FC ≤ −1. Each cell corresponds to the q-value of enrichment (shown in different shades of blue). Black cells indicate missing annotation.

**Figure 8:**
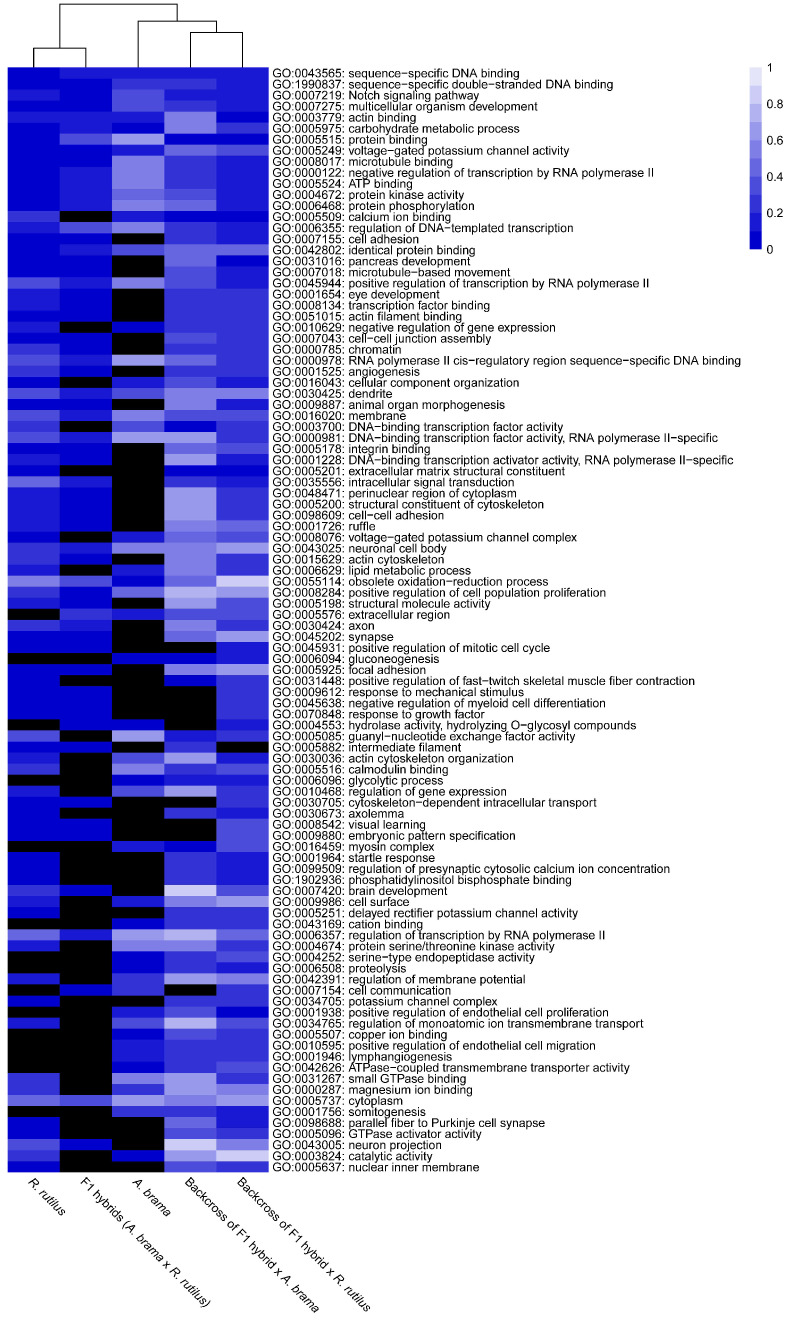
Heatmap of the statistical significance of GO enrichment analysis based on upregulated genes only for infected vs. control comparison. DE genes filtered by FDR ≤ 0.05 and log2FC ≥ 1. Each cell corresponds to the q-value of enrichment (shown in different shades of blue). Black cells indicate missing annotation.

The top 10 enriched GO terms in three main categories (biological process, cellular component and molecular function) for all fish lines are shown in Figure 5 for downregulated genes and in Figure 6 for upregulated genes. The most important of the top 10 enriched GO terms representing downregulated and upregulated genes were shared among fish lines. However, backcross hybrids were more similar to each other than to the three remaining groups (Figure 5, Figure 6, Figure 7 and Figure 8).

From a total of 12,590 annotated genes, 174 genes were differently expressed in the pure line of *A. brama*, including 17 downregulated and 157 upregulated genes (Figure S1a–c). Concerning the blood-feeding strategy of *P. homoion*, erythrocyte differentiation (2/17 downregulated genes) within the biological process category may play a significant role in fish (Figure 5 and Figure S1b). The main contributions of the GO terms representing upregulated genes were recognized for *A. brama* as follows: an obsolete oxidation-reduction process (33/157 genes) within biological process category, an extracellular region (7/157 genes) in the cellular component and oxidoreductase activity (15/157) within the molecular function category (Figure S1c). Upregulated genes potentially involved in *P. homoion* infection also represent iron ion binding (12/157 genes) and heme binding (7/157 genes).

From a total of 12,590 annotated genes, 84 genes were differently expressed in the pure line of *R. rutilus*, including 28 downregulated and 56 upregulated genes (Figure S2a–c). The main contributions of the top enriched GO terms representing downregulated and upregulated genes in *R. rutilus* were similar to those recognized for *A. brama* (Figure 5 and Figure 6). Specifically, the main contribution of the top enriched GO terms representing upregulated genes in *R. rutilus* (Figure S2c) was recognized as follows: protein phosphorylation (8/56 genes) in the biological process category and protein binding (21/56) and protein kinase activity (8/56) in the molecular function category. Analysing all DEGs in *R. rutilus* (Figure S2a), negative regulation of response to cytokine stimulus (1/84) and erythrocyte differentiation (2/84) were revealed among the top 10 GO terms within the biological process category. Erythrocyte differentiation (2/28 genes in *R. rutilus*) within the biological process category was also among the top 10 enriched GO terms representing downregulated genes shared among fish lines (Figure 5 and Figure S2b).

From a total of 12,590 annotated genes, 55 genes were differently expressed in F1 hybrids with *A. brama* mtDNA (F1 *A. brama* × *R. rutilus*), including 33 downregulated and 22 upregulated genes (Figure S3a–c). The main contributions of the top enriched GO terms representing downregulated and upregulated genes in F1 hybrids were the same as those recognized for *A. brama* and *R. rutilus* (Figure 5 and Figure 6). Heme binding (3/33 genes) was recognized among the top 10 enriched GO terms representing downregulated genes found in F1 hybrids (Figure S3b). There were no obvious differences among the contributions of the top 10 enriched GO terms representing upregulated genes within any of three major GO functional categories (Figure S3c).

From a total of 12,590 annotated genes, 382 genes were differently expressed in the backcross generation resulting from the crossing of female F1 hybrids with *A. brama* mtDNA and male *R. rutilus*, including 92 downregulated and 290 upregulated genes (Figure S4a–c). The contributions of the main GO terms representing downregulated genes was the same as those recognized for *A. brama*, *R. rutilus* and F1 *A. brama* × *R. rutilus*. Using the classification of GO terms for downregulated genes in the backcross generation (F1 hybrid × *R. rutilus*), important contributions of immune response and erythrocyte differentiation (4/92 genes for each) within the biological process category and heme binding (8/92 genes) and chemokine activity (3/92 genes) in the molecular function category were also recognized (Figure S4b). Concerning GO terms representing upregulated genes in the backcross generation (F1 hybrid × *R. rutilus*), important contributions of signal transduction in the biological process category (19/290) and protein kinase activity (20/290), protein binding (111/290) and calcium ion binding (26/290) in the molecular function category were found (Figure S4c).

From a total of 12,590 annotated genes, 508 genes were differently expressed in the backcross generation resulting from the crossing of female F1 hybrids with *A. brama* mtDNA and male *A. brama*, including 167 downregulated and 336 upregulated genes (Figure S5a–c). The main GO terms representing downregulated genes were the same as those recognized for *A. brama*, *R. rutilus*, F1 *A. brama* × *R. rutilus* and the backcross with *R. rutilus* in the paternal position; however, the overall pattern in the top 10 GO terms was mostly similar between the two backcross hybrid generations (Figure 5 and Figure 6). On the basis of the top 10 enriched GO terms for backcrosses of F1 hybrid × *A. brama*, we evidenced the roles of immune response and erythrocyte differentiation (7/167 downregulated genes each) within the biological process category and chemokine activity (5/167 genes) within the molecular function category. Similarly, as reported for backcrosses of F1 hybrid × *R. rutilus*, protein binding (108/336 genes) and calcium ion binding (36/336 genes) within the molecular function category were among the top 10 enriched GO terms representing upregulated genes for backcrosses of F1 hybrid × *A. brama*.

On the basis of the heatmap showing the top 100 GO terms from all five fish lines studied (Figure 7 and Figure 8), we identified more similarities between *A. brama* and *R. rutilus* when compared to the profiles of hybrid generations. Five of the above-mentioned downregulated gene based enrichment profiles of GO terms expressed the same pattern for both backcross hybrid lines but were not annotated in the lines of *A. brama*, *R. rutilus* and F1 hybrids. On the basis of the heatmap showing the top 100 GO terms with upregulated genes (Figure 8), GO profiles were more similar between two backcross generations when compared to the profiles of other fish lines.

**Figure 9:**
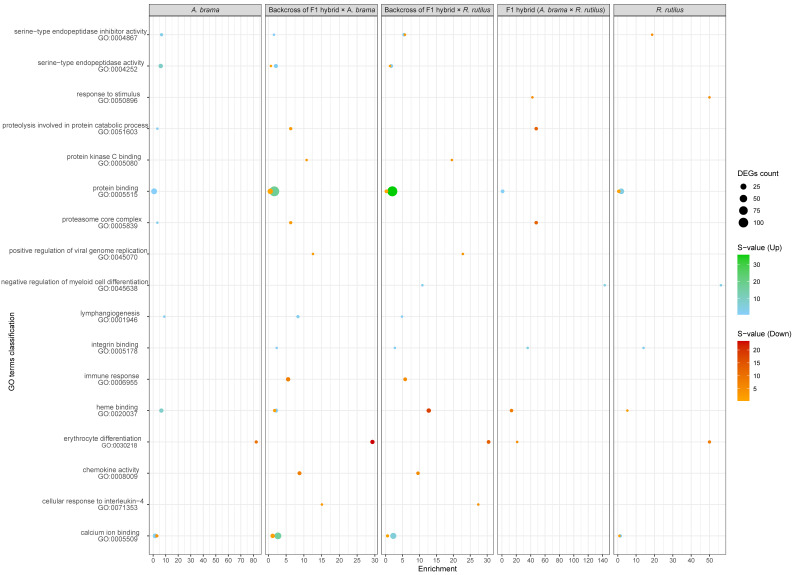
Selection of enriched GO terms potentially related to parasite infection. The plot shows enrichment scores based on upregulated (blue to green colours) and downregulated (orange to red colours) genes separately. The colours show statistical significance in terms of the s-value (−log2(q-value)), and the size shows the number of DE genes behind a particular GO annotation. The enrichment score is computed as ((number of DE genes with particular GO annotation divided by number of all DE genes)/(number of all genes with particular GO annotation divided by number of all genes)). DE genes filtered by FDR ≤ 0.05 and the absolute value of log2FC ≥ 1.

We identified potential parasite infection-related GO terms based on downregulated genes (Figure 7 and Figure 9): GO:0030218—erythrocyte differentiation, GO:0006955—immune response, GO:0050896—response to stimulus, GO:0071353—cellular response to interleukin−4, GO:0045070—positive regulation of viral genome replication and GO:0051603—proteolysis involved in protein catabolic process within the biological processes GO functional category; GO:0008009—chemokine activity, GO:0020037—heme binding, GO:0004867—serine−type endopeptidase inhibitor activity and GO:0005080—protein kinase C binding within the molecular function GO functional category and GO:0005839—proteasome core complex within the cellular component GO functional category. We identified GO terms based on upregulated genes potentially involved in infection by blood-feeding parasites (Figure 8 and Figure 9), specifically, GO:0045638—negative regulation of myeloid cell differentiation and GO:0001946—lymphangiogenesis (both within biological process GO category) and GO:0005509—calcium ion binding, GO:0005515—protein binding, GO:0005178—integrin binding, GO:0004252—serine−type endopeptidase activity within the molecular function GO category. Backcross generations exhibited obvious similarity in expression profiles, including protein kinase C binding, protein binding, positive regulation of viral genome replication, immune response, chemokine activity and cellular response to interleukin-4 (Figure 9).

**Figure 10:**
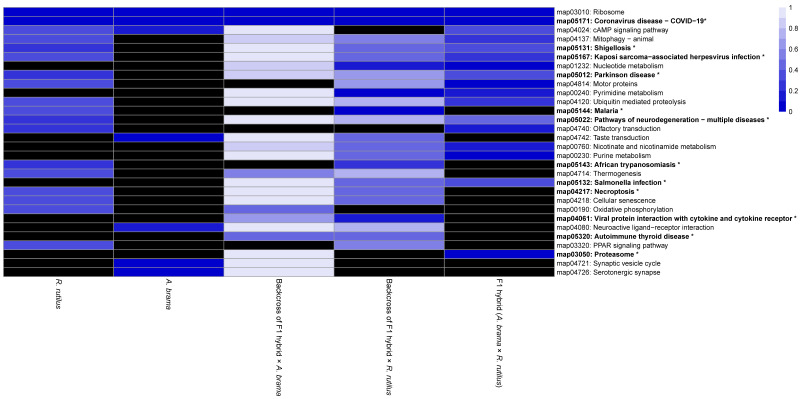
Heatmap of statistical significance of KEGG pathway enrichment analysis based on downregulated genes only for infected vs. control comparison. DE genes filtered by FDR ≤ 0.05 and log2FC ≤ −1. The plot shows the top 30 enriched KEGG pathways among all fish lines. Each cell corresponds to the q-value of enrichment (shown in different shades of blue). Black cells indicate missing annotation. Pathways related to disease are shown in bold with asterisk.

**Figure 11:**
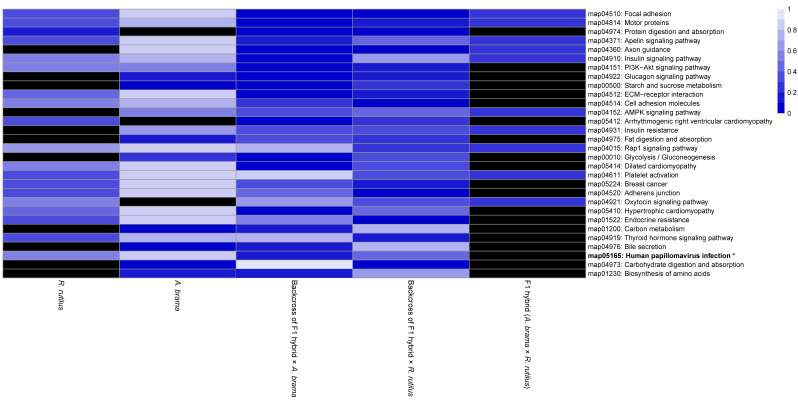
Heatmap of statistical significance of KEGG pathway enrichment analysis based on upregulated genes only for infected vs. control comparison. DE genes filtered by FDR ≤ 0.05 and log2FC ≥ 1. The plot shows the top 30 enriched KEGG pathways among all fish lines. Each cell corresponds to the q-value of enrichment (shown in different shades of blue). Black cells indicate missing annotation. Pathways related to disease are shown in bold with asterisk.

The heatmap from KEGG pathway enrichment analysis for significantly downregulated genes with the top 30 KEGG pathways enriched among all fish lines is shown in Figure 10. The majority of pathways were not identified in *A. brama*; 12 in *R. rutilus* and 13 in F1 hybrids were missing. The missing pathways were mainly shared between *A. brama* and F1 hybrids or between *A. brama* and *R. rutilus*. From this list of enriched KEGG pathways, 10 of them were associated with diseases and one with the potential immune response of hosts (Figure 10). The level of enrichment for these pathways differed between two backcross generations; all of these pathways were present only in backcross hybrids with *R. rutilus* in the paternal position. The heatmap from KEGG pathway enrichment analysis for significantly upregulated genes with the top 30 KEGG pathways enriched among all fish lines is shown in Figure 11. The majority of pathways were not identified in F1 hybrids; 11 in *R. rutilus* and 3 in *A. brama* were missing. A large difference in the level of enrichment was found between *A. brama* and other lines. In contrast, the most similar enrichment for the top 30 KEGG pathways representing upregulated genes was found between two backcross generations. Only one pathway was identified to be related to disease (Figure 11).

GEO project with accession number GSE240906, including gene counts, DE results and output tables of GO and KEGG enrichment is included at https://www.ncbi.nlm.nih.gov/geo/query/acc.cgi?acc=GSE240906.

Because of no obvious differences in differentially expressed genes between some pairs of fish lines and because of high intra-line variability in *P. homoion* infection, the correlation between the relative expression changes upon infection for each fish individual (gene density) and *P. homoion* infection was calculated for each gene. Using a correlation coefficient (r > 0.65 and *p* ≤ 0.001), 10 genes with a relevant biological function (checked using published studies [88,89,90,91,92,93,94,95,96,97,98,99,100,101]) were selected for qPCR validation, 8 of them exhibiting a positive correlation and 2 of them a negative correlation with *P. homoion* infection. Two of these selected genes (*YQAJ* and *MAM*) were finally removed from the analyses because of unspecific PCR amplification. The remaining eight genes were used for the confirmation of gene expression using qPCR (Table 1 and Table 2). Using gene expression data from qPCR, there was a positive significant correlation among differentially expressed genes and *P. homoion* infection for 5 genes (*HBB* (haemoglobin), *CCND1*, *ING*, *SCAMP5a* and *ENTH*). Even though insignificant, there was a trend of negative correlation among the gene expressions of *IL34* and *GSN* (gelsolin) and *P. homoion* infection.

## 4. Discussion

The present study focussed on changes in the transcriptome profile of head kidney (an important immune organ of fish) after infection by the blood-feeding monogenean parasite *P. homoion*. Using artificially prepared fish lines, we compared levels of parasite infection (measured at the end of experiment) and changes in the transcriptome profiles of head kidney between control and infected specimens in the lines of two leuciscid species, *A. brama* and *R. rutilus*, and their F1 and backcross hybrid lines. We expected differences in parasite load and transcriptome profile response (i.e., differential gene expression) between F1 hybrids and backcross hybrids, hypothesizing hybrid advantage for F1 hybrids and hybrid breakdown for backcross hybrids.

Our study revealed differences in the presence of larval (diporpae) and adult stages of *P. homoion* and differences in the numbers of parasites among fish lines, suggesting that potential of *P. homoion* to finalize its life cycle and/or parasite survival may vary among fish lines. No adults were documented on specimens of *A. brama* and specimens of F1 hybrids, likely indicating a more effective immune response in *A. brama* and a potential hybrid advantage for F1 hybrids when infected by *P. homoion*. The lower susceptibility of F1 hybrids to monogenean infection (reflected by lower numbers of parasites on fish) when compared to the susceptibility of parental fish species was previously documented [13,16,24,26] and interpreted in line with heterosis advantage, the hybrid resistance scenario (defined by Fritz et al. [102]) or host–parasite coadaptation.

The presence of adult specimens and their effective reproduction, evidenced by the presence of a new generation of diporpae, support the evidence that *R. rutilus* is a suitable host for *P. homoion* survival and reproduction. Some previous studies suggest the effect of host mtDNA for resistance to parasites [26,103,104]; however, our study was focussed on hybrid lines all of them expressing the mtDNA of less susceptible host, *A. brama*. We reported overall higher susceptibility of backcross hybrids when compared to the F1 generation of hybrids. This may indicate potential consequences of hybrid breakdown in backcross generations of hybrids, which is also supported by the highest numbers of *P. homoion* specimens in some backcross specimens, the highest proportion of infected host specimens within backcross lines and the highest potential to finalize life cycle of *P. homoion* on backcross hybrids (presence of the highest number of adults). *Rutilus rutilus* has been reported as the most common host of *P. homoion* (33 reports included in the Host–Parasite Database, Natural History Museum https://www.nhm.ac.uk/research-curation/scientific-resources/taxonomy-systematics/host-parasites/database/, accessed on 30 May 2023). In contrast, *A. brama* is less frequently parasitized by *P. homoion* (3 reports included in the Host–parasite Database, Natural History Museum); however, *A. brama* is a common host of another representative of Diplozoidae, i.e., *Diplozoon paradoxum* (41 reports). This may suggest some (even if not strong) potential coevolutionary associations in the case of generalist parasites, specifically among *P. homoion* and the most common host species, *R. rutilus*. The pattern of coevolutionary associations was previously suggested to affect the parasite load of host-specific parasites even in leuciscid hybrids [13,16,24,25,26]. Dedic et al. [16] investigated the susceptibility of F1 and backcross hybrids of *A. brama* and *R. rutilus* to monogenean parasites associated with *A. brama* or with *R. rutilus* and expected the high parasite abundance in post-F1 generations. However, they revealed that genetic incompatibilities do not play a significant role in the infection of backcross hybrids by host-specific parasites and, rather, that the level of infection by host-specific monogeneans in various backcross generations of hybrids seems to be driven by host–parasite coevolutionary interactions. The role of coadaptation between host genotype and host-specific parasites was also highlighted to explain the asymmetrical distribution of different species of parental-specific parasites in fish hybrids [13,16,26]. However, Krasnovyd et al. [13] suggested that parasite species infecting a wider range of host species (generalists), in contrast to host-specific parasites, may confer disadvantages to fish hybrids. As genetic host–parasite coadaptation is unexpected or is expected in less extent for generalist parasites (we highlighted some extent of potential coadaptation between *P. homoion* and *R. rutilus*), they may represent more useful candidates in the studies investigating the hybrid heterosis or hybrid breakdown. Our study revealed higher infection of generalist parasite *P. homoion* in backcross hybrids when compared to specimens of parental species and F1 hybrids.

We reported high variability in *P. homoion* numbers among fish specimens within fish lines, especially for the two backcross hybrid generations, indicating intra-line variability in host susceptibility to *P. homoion* infection (however, almost all backcross hybrid specimens were infected by *P. homoion*). Explanation of this observation would require further study. As we had no opportunity to perform the experiment with higher numbers of fish specimens within fish lines, i.e., to perform an experiment which may allow us to sample fish specimens at different time points from infection, we cannot exclude the possibility that other fish specimens were initially infected (or at least affected) by oncomiracidia attaching to fish skin. Therefore, we performed transcriptome profile analyses of head kidney, considering also the fact that some fish specimens were exposed to *P. homoion* and others were non-exposed to *P. homoion* (control group).

In our study, we focused on transcriptome profile analyses to reveal the DEGs involved in infection by *P. homoion*, a blood-feeding parasite, which was previously neglected when investigating fish immune response, likely because of its generally lower parasite load when compared to other monogeneans (i.e., *Gyrodactylus* or *Dactylogyrus* spp.) infecting cyprinoid fish. However, even diplozoids may negatively affect fish vigour; for example, hypochromic microcytic anaemia characterized by a high percentage of immature erythrocytes was reported in crucian carp (*Carassius carassius*) because of *Eudiplozoon nipponicum* infection (representative of Diplozooidae) [17]. The objective of our study was to focus on DEGs in the head kidney of fish hosts associated with *P. homoion* infection in species lines (two phylogenetically distant species, *A. brama* and *R. rutilus*) and hybrid lines (including F1 and backcross generations of hybrids) under controlled conditions in which fish received the same initial infection dose of parasites. We expected relatively large parasites exhibiting a blood-feeding strategy to have a noticeable effect on fish hosts. Even though we found relatively low parasite loads in fish specimens by the end of our experiment (with some exceptions), which can raise questions about the effect of *P. homoion* on the immune response of fish hosts, our previous experiment involving *Dactylogyrus* infection on gibel carp (*Carassius gibelio*) revealed changes in the expression of immunity-associated genes (measured by DEGs using the transcriptome profiles of spleen) after fish were exposed to oncomiracidia of monogeneans but without the presence of parasites on gills at the time of fish dissection (21 days post infection, dpi) [89]. Potrok et al. [89] explained the absence of monogeneans on the gills of experimental fish as a potential effect of fish immune system likely eliminating the parasite after 21 dpi or as a result of unsuitable conditions for parasite survival in experiments. Oncomiracidium is a short-living stage which has to reach the hosts within a few hours after its transfer to the water with suitable host. Since expression levels of selected immune-related genes differed between control (uninfected) and infected fish and different infection dose in the study of Potrok et al. [89], they presumed that infection was successfully established at some time point during experiment.

Blood-feeding parasites have developed biochemical mechanisms to control heme intake and detoxification and diverse strategies for detoxifying excess heme derived from the catabolism of host red cells (e.g., [105,106]). The mechanism of *P. homoion* digestion is predominantly intracellular and specialised cells like hematin cells degrade globin into particular amino acids and release the residues of metabolized heme in the form of hematin [12]. The haemoglobin levels of host fish decreased with increasing numbers of diplozoid parasites [17,18]. Vorel et al. [107], using transcriptome and secretome analyses of *Eudiplozoon nipponicum*, revealed that this parasite abundantly expresses several key peptidases, including cathepsins B, D, L1 and L3, which play a critical role in haemoglobin processing, and highlighted that ferritin proteins and GSTs play an important role in the life of adult *E. nipponicum*, particularly in iron and heme processing. In hosts themselves, heme binding proteins are important components for heme detoxification and for the recycling of heme and iron since they allow the targeting of heme to specific tissues such as the liver [108,109]. Therefore, we focused on the DEGs related to biological processes and molecular function associated with fish blood. We recognized heme binding among the top 25 enriched GO terms in connection with *P. homoion* infection. For some fish lines, we even found iron binding among the top 10 GO terms. However, DEGs in heme binding varied from upregulated (*A. brama*) to downregulated (*R. rutilus*, F1 hybrids and backcross hybrids with *R. rutilus* in paternal position), whilst both up- and downregulated genes were recognized in backcross hybrids with *A. brama* in paternal position in our study. This may suggest potential reciprocal molecular interactions between *P. homoion* and its common host *R. rutilus* (and, subsequently, F1 hybrids and backcross hybrids with *R. rutilus* in paternal position—two hybrid lines with higher proportions of *R. rutilus* genes when compared to backcross hybrids with *A. brama* in paternal position). Our analysis of DEGs also revealed the importance of erythrocyte differentiation in fish following *P. homoion* infection; this effect was less obvious in less infected F1 hybrids when compared to more infected backcross hybrid generations, potentially supporting heterosis advantage for F1 hybrids.

Overall transcriptome profile changes after *P. homoion* infection (using DEGs classified on the basis of GO terms and top 30 KEGG pathways in fish lines) were more similar between two backcross hybrid generations than between other pairs of fish lines. We also evidenced a higher number of DEGs (both downregulated and upregulated) in two backcross hybrid generations when compared to *A. brama*, *R. rutilus* and F1 hybrids, indicating that the transcriptome profiles of backcross generations were more affected by *P. homoion* (by larval stages throughout host invasion or later during ontogenetic development to blood-feeding large adult) compared to species lines and F1 hybrid line. We can hypothesize that the observed pattern is a potential consequence of hybrid breakdown; however, we highlight the necessity of future studies to investigate the molecular background of this phenomenon.

In the present study, the similarity in DEGs between two backcross hybrid generations with higher *P. homoion* infection was mostly evidenced for immunity-related biological processes and potential components of molecular host–parasite interactions (specifically, immune response, chemokine activity, cellular response to interleukin-4, the positive regulation of viral genome replication, serine-type endopeptidase activity, protein kinase C binding and protein binding). The role of immunity-associated pathways in fish defence against *P. homoion* infection was also confirmed using a few immune genes included in the set of genes selected on the basis of the correlation between RNA seq-gene expression and *P. homoion* infection and then validated by the qPCR approach. Elucidating the roles of cytokines (including interleukins participating in the regulation of immune processes and chemokines—small cytokines with a chemotactic effect) in the immune defence of fish against various pathogens (bacteria, protozoans and metazoans) using transcriptomic analysis and/or qPCR has been well documented recently (e.g., [41,110,111,112,113]). However, current knowledge on immune gene expression in fish as a response to monogenean infection is still limited despite the economic impacts of some monogenean species in aquacultures (e.g., [3,4,6]). To measure the expression of immunity-associated genes, the skin and gills (e.g., [43,44,114]) or spleen and head kidney (considered as the most important immune organs in fish), [40,41] have been used. In cyprinid fish, specifically, goldfish (*Carassius auratus*), the pro-inflammatory factors IL-1β2, TNFα1 and TNFα2 were shown to be upregulated in fish skin during infection by the viviparous monogenean *Gyrodactylus kobayashii* during first few days post-infection (7 dpi–14 dpi), though their expressions did not differ significantly between the infected and control groups at later stages of infection (21 dpi–28 dpi) [43]. In a recent study, Zhou et al. [42] investigated the immune mechanisms of goldfish against *G. kobayashii* using transcriptome profile analyses of fish skin and identified 14 pathways associated with immune response to *G. kobayashii*. Using the same host–parasite system, Tu et al. [41] documented higher expressions of the immunomodulators TGFβ and IL10 responsible for decreasing inflammation and damage to tissues caused by inflammation in later stages of *Gyrodactylus* infection. The expression of genes encoding toll-like receptors, i.e., receptors able to recognize a wide spectrum of antigens, was documented during infection by monogenean parasites. Goldfish during infection by the oviparous monogenean *Dactylogyrus intermedius* expressed high TLR4, TLR5, TLR20 and TLR22 in gills, head kidney, liver and spleen [115]. In the host *Seriola lalandi* infected by the monogenean *Neobenedenia melleni*, the expressions of TLR3, TLR9, TLR21 and TLR22 in spleen were significantly higher than in control fish [116].

The role of serine peptidases in fish host defence is well known, and was recently documented against monogenean parasites (e.g., *G. kobayashii* in goldfish [117]). Parasites regulate the activities of host serine peptidases for their own benefit, employing various inhibitors. In the case of blood-feeding *E. nipponicum*, the presence of potential anticoagulants, i.e., Kunitz protein or serpins—protease inhibitors impairing hemostasis, blood coagulation, fibrinolysis and the function of fish complement—was found [118,119]. In accordance with these studies, we found serine-type endopeptidase activity among the top enriched GO terms related to *P. homoion*; in particular, the two backcross generations shared similarity in this GO term. Protein kinases involved in fish host immune defence and protein kinase inhibitor activity involved in signal transduction pathways in fish parasites have previously been documented to play a role in virulence and pathogenesis (e.g., [120,121]); however, to our knowledge, no study of protein kinase activity in fish infected by monogeneans and/or protein kinase inhibitors in monogeneans has been performed.

KEGG pathway analyses performed in our study showed that *P. homoion* infection–regulated genes were related to a wide range of human disease pathways. Specifically, 10 human disease-associated pathways (linked to immunity disorder) among the 30 top pathways with downregulated genes and 1 human disease-associated pathway among the 30 top pathways with upregulated genes were identified (with some other pathways corresponding to non-parasitic diseases), almost all of them shared by the two backcross hybrid lines. Comparison of the zebrafish genome to the human reference genome revealed that approximately 70% of human genes have at least one obvious zebrafish orthologue [122]. Therefore, zebrafish mutations faithfully phenocopy many human disorders [123]. Recently, genes related to human diseases have been identified in the genome of zebrafish (e.g., [124]). Previous studies focussed on DEGs linked to pathogens in fish documented their associations to human diseases (e.g., [125]), and similar associations were also reported among DEGs and hypoxia stress in fish [126]. Our study indicates that even DEGs induced by monogenean infection in fish are linked to immune diseases reported in humans. In line with the hybrid vigour hypotheses, we evidenced such associations mostly in backcross hybrid generations, potentially reflecting hybrid breakdown.

## 5. Conclusions

We presented an original study focussed on changes in the transcriptome profile of head kidney (used for DEGs) in two freshwater fish species, *A. brama* and *R. rutilus*, and their F1 and backcross hybrid lines after infection by the blood-feeding generalist monogenean parasite *P. homoion*. The number of DEGs was lower in F1 hybrids and higher in backcross hybrids when compared to each of the parental lines. The main categories of DEGs were shared among lines; however, *A. brama* and *R. rutilus* differed in some of the top GO terms. DEG analyses highlighted the role of heme binding, erythrocyte differentiation and immunity-related processes after infection by blood-feeding *P. homoion*. KEGG analysis revealed disease-associated pathways, almost all of them shared by backcross hybrids. We revealed the most pronounced DEGs associated with blood-feeding *P. homoion* in backcross hybrids, potentially (but not exclusively) explainable by hybrid breakdown. The gene expression of F1 hybrids that were less infected by *P. homoion* when compared to backcross hybrids is in the line of hybrid advantage.

## Figures and Tables

**Table 2:** Correlation between gene expression and *P. homoion* abundance. Significant correlations (*p* < 0.05) are shown in bold.

	qPCR		RNA seq	
Gene Name	R	*p*	R	*p*
*HBB*	0.951	**<0.001**	0.651	**0.001**
*CCND1*	0.906	**<0.001**	0.851	**<0.001**
*ING*	0.709	**0.007**	0.845	**<0.001**
*IL34*	−0.342	0.253	−0.645	**0.001**
*ZK-C2H2*	0.172	0.575	0.787	**<0.001**
*GSN*	−0.515	0.072	−0.788	**<0.001**
*SCAMP5a*	0.747	**0.003**	0.792	**<0.001**
*ENTH*	0.806	**0.001**	0.781	**<0.001**

## Data Availability

The data used in this study have been deposited in NCBI’s Gene Expression Omnibus [127] and are accessible through GEO Series accession number GSE240906 (https://www.ncbi.nlm.nih.gov/geo/query/acc.cgi?acc=GSE240906).

## Supplementary Materials

The following supporting information can be downloaded at: https://www.mdpi.com/article/10.3390/biology12091199/s1. File S1: RNA-seq data primary analysis details; File S2: Custom rRNA database details; File S3: The transcriptome analysis details; Table S1: The results of the alignment to *Cyprinus carpio* genome; Table S2: The results of the alignment to *Carassius auratus* genome; Table S3: The results of the alignment to *Abramis brama* transcriptome; Table S4: The results of the alignment to *Rutilus rutilus* transcriptome; Table S5: rnaQUAST statistics of *R. rutilus* transcriptome assemblies; Table S6: rnaQUAST statistics of *A. brama* transcriptome assemblies; Table S7: BUSCO statistics of *R. rutilus* transcriptome assemblies; Table S8: BUSCO statistics of *A. brama* transcriptome assemblies; Script S1: GO enrichment analysis results postprocessing code in R language. Script S2: KEGG enrichment analysis results postprocessing code in R language. Figure S1: Top 10 enriched GO terms within three functional GO categories for *A. brama* based on: a—all differentially expressed genes, b—downregulated genes, c—upregulated genes; Figure S2: Top 10 enriched GO terms within three functional GO categories for *R. rutilus* based on: a—all differentially expressed genes, b—downregulated genes, c—upregulated genes; Figure S3: Top 10 enriched GO terms within three functional GO categories for F1 hybrids (*A. brama* × *R. rutilus*) based on: a—all differentially expressed genes, b—downregulated genes, c—upregulated genes, Figure S4: Top 10 enriched GO terms within three functional GO categories for backcross generation resulting from the crossing of female F1 hybrids with *A. brama* mtDNA and male *R. rutilus* based on: a—all differentially expressed genes, b—downregulated genes, c—upregulated genes, Figure S5: Top 10 enriched GO terms within three functional GO categories for female F1 hybrids with *A. brama* mtDNA and male *A. brama* based on: a—all differentially expressed genes, b—downregulated genes, c—upregulated genes.
