# Supplementary material for: Transcriptome Profile Analyses of Head Kidney in Roach (Rutilus rutilus), Common Bream (Abramis brama) and Their Hybrids: Does Infection by Monogenean Parasites in Freshwater Fish Reveal Differences in Fish Vigour among Parental Species and Their Hybrids?
Source: Biology (Basel). 2023 Sep 1;12(9):1199. doi: 10.3390/biology12091199 (PMC10525477; doi:10.3390/biology12091199)
Supplement: Supplementary file 1 [file biology-12-01199-s001.zip › biology-2509074-supplementary.pdf]

Figure S1a

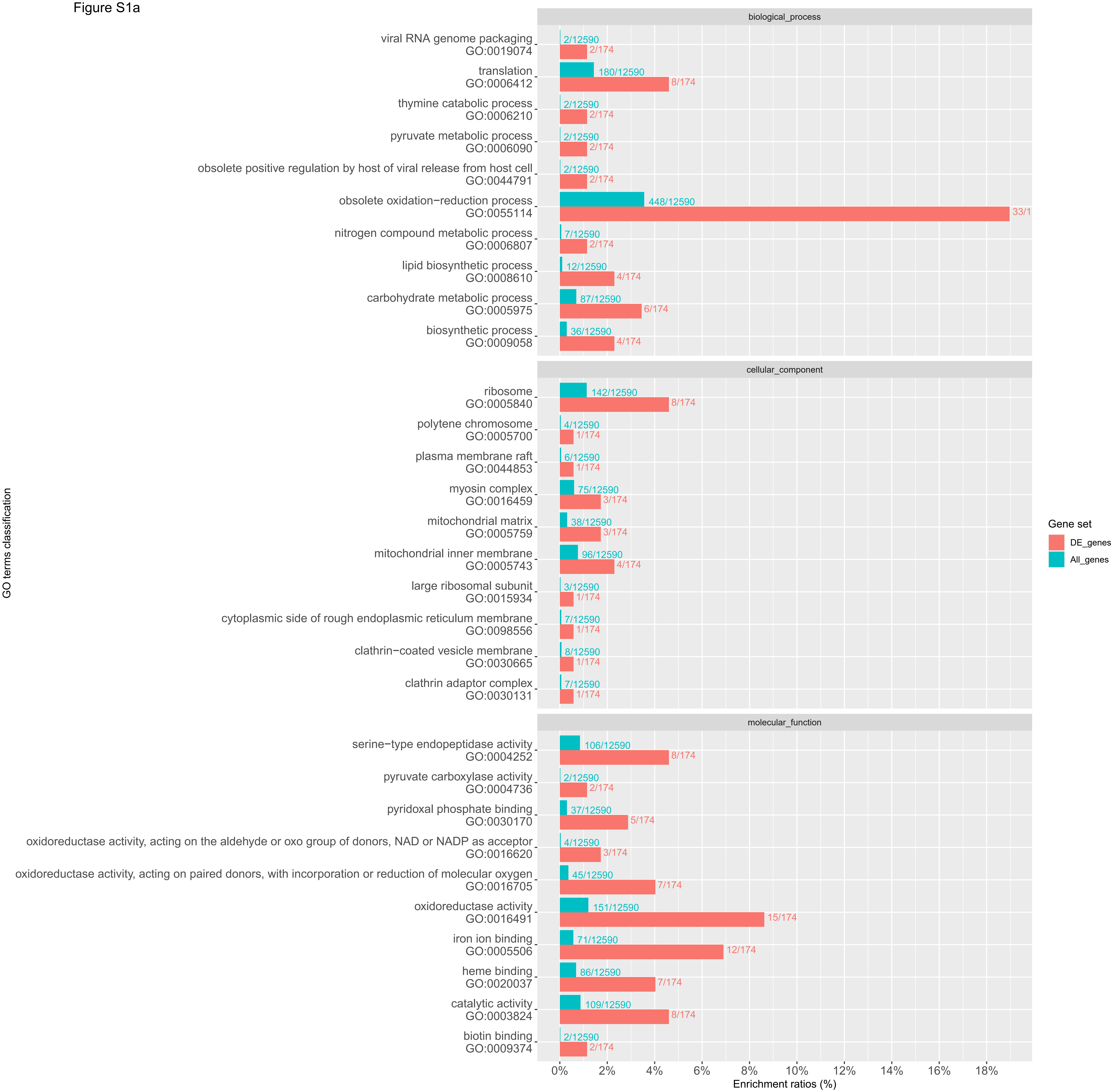

biological_process
viral RNA genome packaging GO:0019074
2/12590
2/174
translation GO:0006412
180/12590
8/174
thymine catabolic process GO:0006210
2/12590
2/174
pyruvate metabolic process GO:0006090
2/12590
2/174
obsolete positive regulation by host of viral release from host cell GO:0044791
2/12590
2/174
obsolete oxidation−reduction process GO:0055114
448/12590
33/1
nitrogen compound metabolic process GO:0006807
7/12590
2/174
lipid biosynthetic process GO:0008610
12/12590
4/174
carbohydrate metabolic process GO:0005975
87/12590
6/174
biosynthetic process GO:0009058
36/12590
4/174
cellular_component
ribosome GO:0005840
142/12590
8/174
polytene chromosome GO:0005700
4/12590
1/174
plasma membrane raft GO:0044853
6/12590
1/174
myosin complex GO:0016459
75/12590
3/174
mitochondrial matrix GO:0005759
38/12590
3/174
mitochondrial inner membrane GO:0005743
96/12590
4/174
large ribosomal subunit GO:0015934
3/12590
1/174
cytoplasmic side of rough endoplasmic reticulum membrane GO:0098556
7/12590
1/174
clathrin−coated vesicle membrane GO:0030665
8/12590
1/174
clathrin adaptor complex GO:0030131
7/12590
1/174
molecular_function
serine−type endopeptidase activity GO:0004252
106/12590
8/174
pyruvate carboxylase activity GO:0004736
2/12590
2/174
pyridoxal phosphate binding GO:0030170
37/12590
5/174
oxidoreductase activity, acting on the aldehyde or oxo group of donors, NAD or NADP as acceptor GO:0016620
4/12590
3/174
oxidoreductase activity, acting on paired donors, with incorporation or reduction of molecular oxygen GO:0016705
45/12590
7/174
oxidoreductase activity GO:0016491
151/12590
15/174
iron ion binding GO:0005506
71/12590
12/174
heme binding GO:0020037
86/12590
7/174
catalytic activity GO:0003824
109/12590
8/174
biotin binding GO:0009374
2/12590
2/174
0%
2%
4%
6%
8%
10%
12%
14%
16%
18%
Enrichment ratios (%)
GO terms classification
Gene set
DE_genes
All_genes

Figure S1b

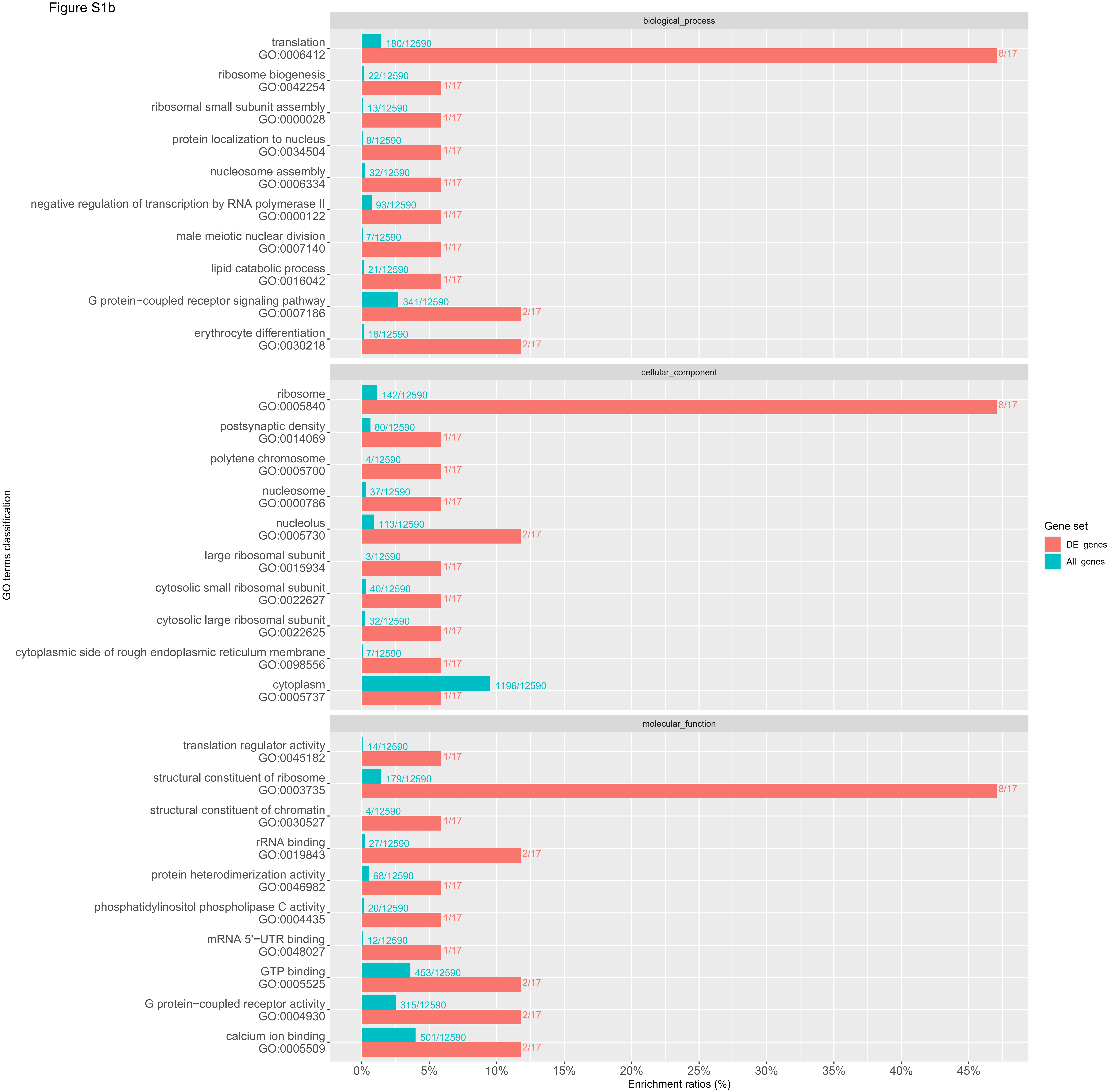
biological_process
translation GO:0006412
180/12590
8/17
ribosome biogenesis GO:0042254
22/12590
1/17
ribosomal small subunit assembly GO:0000028
13/12590
1/17
protein localization to nucleus GO:0034504
8/12590
1/17
nucleosome assembly GO:0006334
32/12590
1/17
negative regulation of transcription by RNA polymerase II GO:0000122
93/12590
1/17
male meiotic nuclear division GO:0007140
7/12590
1/17
lipid catabolic process GO:0016042
21/12590
1/17
G protein−coupled receptor signaling pathway GO:0007186
341/12590
2/17
erythrocyte differentiation GO:0030218
18/12590
2/17
cellular_component
ribosome GO:0005840
142/12590
8/17
postsynaptic density GO:0014069
80/12590
1/17
polytene chromosome GO:0005700
4/12590
1/17
nucleosome GO:0000786
37/12590
1/17
nucleolus GO:0005730
113/12590
2/17
large ribosomal subunit GO:0015934
3/12590
1/17
cytosolic small ribosomal subunit GO:0022627
40/12590
1/17
cytosolic large ribosomal subunit GO:0022625
32/12590
1/17
cytoplasmic side of rough endoplasmic reticulum membrane GO:0098556
7/12590
1/17
cytoplasm GO:0005737
1196/12590
1/17
molecular_function
translation regulator activity GO:0045182
14/12590
1/17
structural constituent of ribosome GO:0003735
179/12590
8/17
structural constituent of chromatin GO:0030527
4/12590
1/17
rRNA binding GO:0019843
27/12590
2/17
protein heterodimerization activity GO:0046982
68/12590
1/17
phosphatidylinositol phospholipase C activity GO:0004435
20/12590
1/17
mRNA 5'−UTR binding GO:0048027
12/12590
1/17
GTP binding GO:0005525
453/12590
2/17
G protein−coupled receptor activity GO:0004930
315/12590
2/17
calcium ion binding GO:0005509
501/12590
2/17
GO terms classification
0% 5% 10% 15% 20% 25% 30% 35% 40% 45%
Enrichment ratios (%)
Gene set
DE_genes
All_genes

Figure S1c

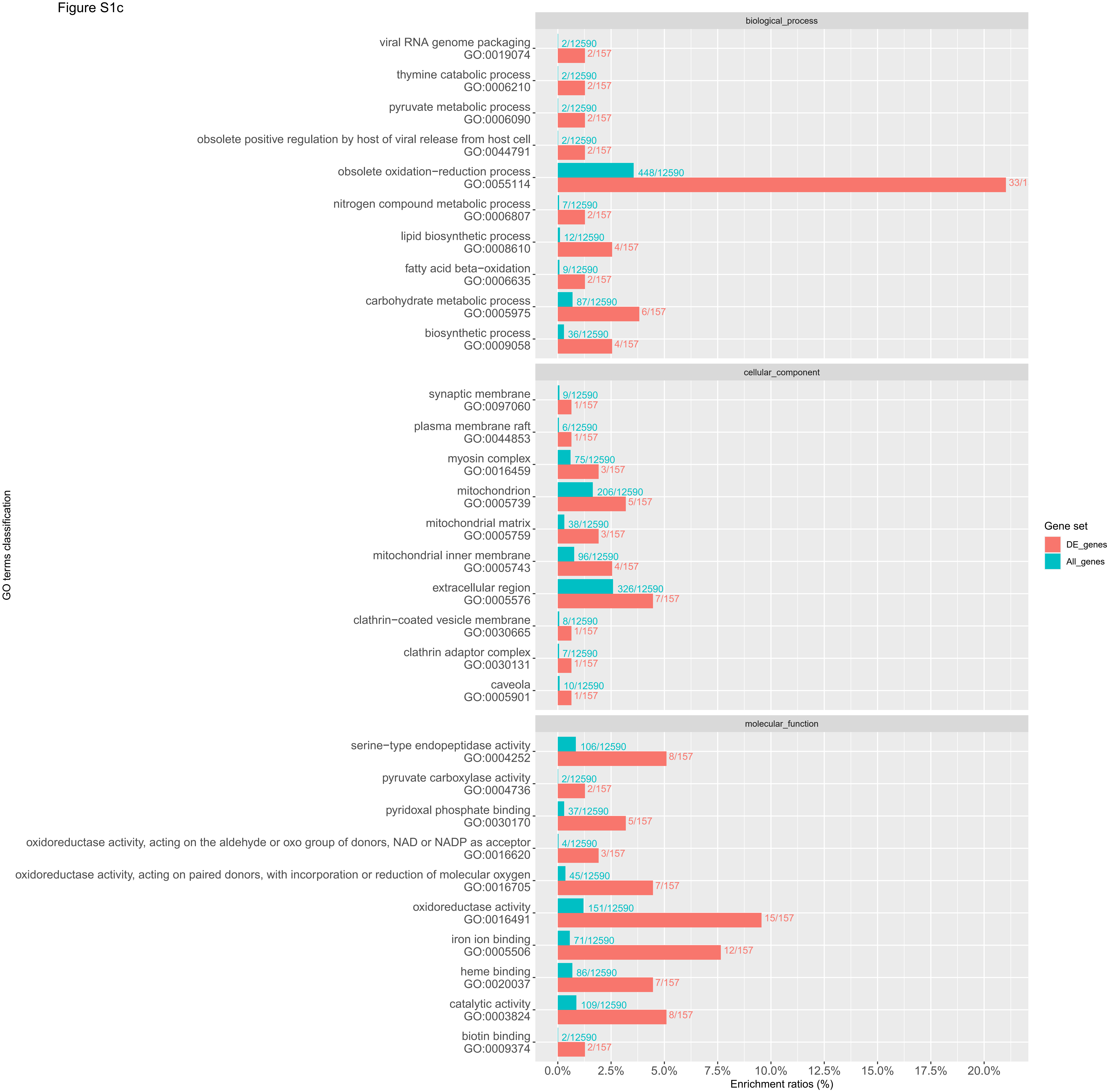
biological_process
viral RNA genome packaging GO:0019074
2/12590
2/157
thymine catabolic process GO:0006210
2/12590
2/157
pyruvate metabolic process GO:0006090
2/12590
2/157
obsolete positive regulation by host of viral release from host cell GO:0044791
2/12590
2/157
obsolete oxidation−reduction process GO:0055114
448/12590
33/1
nitrogen compound metabolic process GO:0006807
7/12590
2/157
lipid biosynthetic process GO:0008610
12/12590
4/157
fatty acid beta−oxidation GO:0006635
9/12590
2/157
carbohydrate metabolic process GO:0005975
87/12590
6/157
biosynthetic process GO:0009058
36/12590
4/157
cellular_component
synaptic membrane GO:0097060
9/12590
1/157
plasma membrane raft GO:0044853
6/12590
1/157
myosin complex GO:0016459
75/12590
3/157
mitochondrion GO:0005739
206/12590
5/157
mitochondrial matrix GO:0005759
38/12590
3/157
mitochondrial inner membrane GO:0005743
96/12590
4/157
extracellular region GO:0005576
326/12590
7/157
clathrin−coated vesicle membrane GO:0030665
8/12590
1/157
clathrin adaptor complex GO:0030131
7/12590
1/157
caveola GO:0005901
10/12590
1/157
molecular_function
serine−type endopeptidase activity GO:0004252
106/12590
8/157
pyruvate carboxylase activity GO:0004736
2/12590
2/157
pyridoxal phosphate binding GO:0030170
37/12590
5/157
oxidoreductase activity, acting on the aldehyde or oxo group of donors, NAD or NADP as acceptor GO:0016620
4/12590
3/157
oxidoreductase activity, acting on paired donors, with incorporation or reduction of molecular oxygen GO:0016705
45/12590
7/157
oxidoreductase activity GO:0016491
151/12590
15/157
iron ion binding GO:0005506
71/12590
12/157
heme binding GO:0020037
86/12590
7/157
catalytic activity GO:0003824
109/12590
8/157
biotin binding GO:0009374
2/12590
2/157
0.0% 2.5% 5.0% 7.5% 10.0% 12.5% 15.0% 17.5% 20.0%
Enrichment ratios (%)
GO terms classification
Gene set
DE_genes
All_genes

Figure S2a

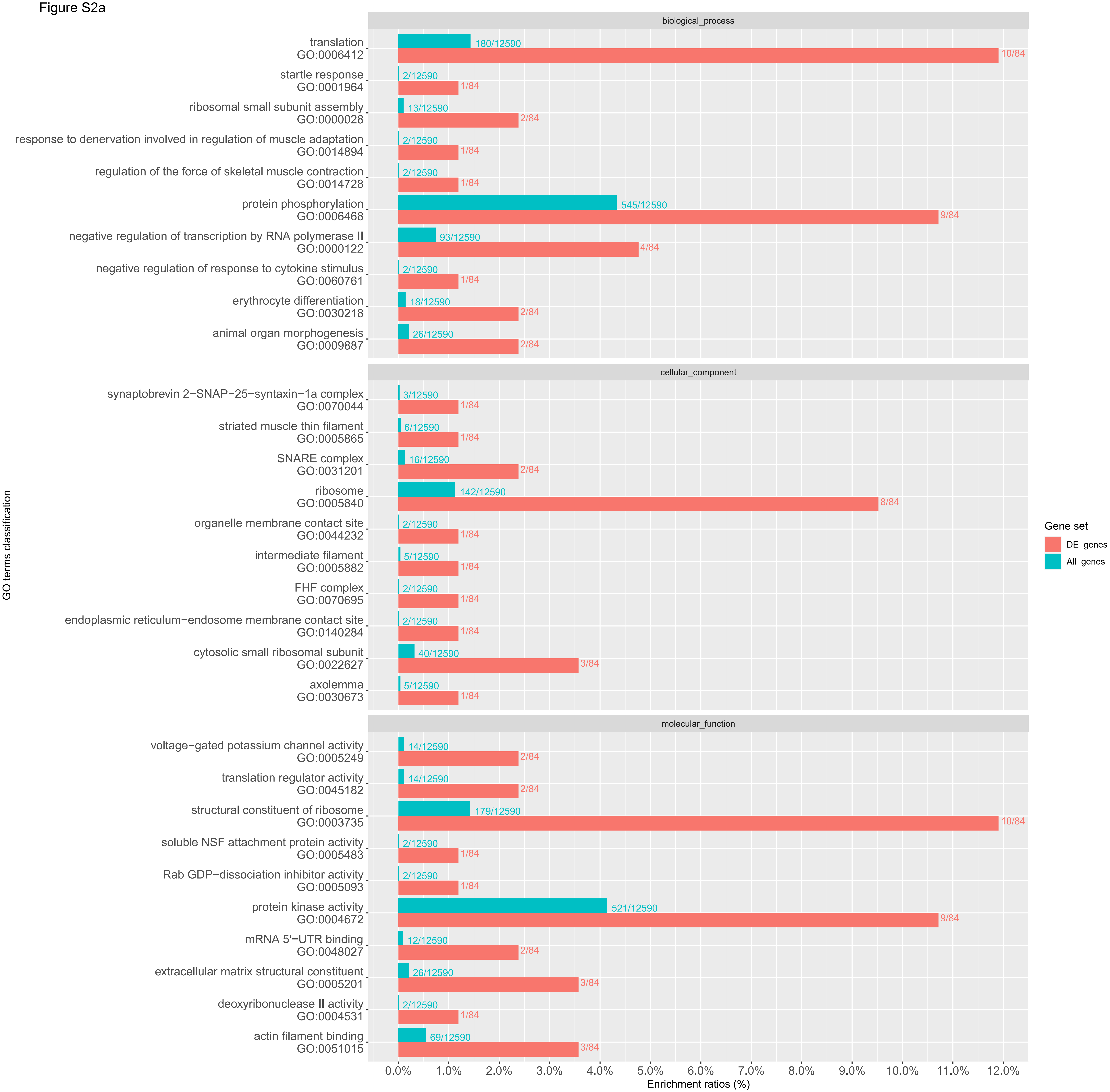
biological_process
translation
GO:0006412
180/12590
10/84
startle response
GO:0001964
2/12590
1/84
ribosomal small subunit assembly
GO:0000028
13/12590
2/84
response to denervation involved in regulation of muscle adaptation
GO:0014894
2/12590
1/84
regulation of the force of skeletal muscle contraction
GO:0014728
2/12590
1/84
protein phosphorylation
GO:0006468
545/12590
9/84
negative regulation of transcription by RNA polymerase II
GO:0000122
93/12590
4/84
negative regulation of response to cytokine stimulus
GO:0060761
2/12590
1/84
erythrocyte differentiation
GO:0030218
18/12590
2/84
animal organ morphogenesis
GO:0009887
26/12590
2/84
cellular_component
synaptobrevin 2−SNAP−25−syntaxin−1a complex
GO:0070044
3/12590
1/84
striated muscle thin filament
GO:0005865
6/12590
1/84
SNARE complex
GO:0031201
16/12590
2/84
ribosome
GO:0005840
142/12590
8/84
organelle membrane contact site
GO:0044232
2/12590
1/84
intermediate filament
GO:0005882
5/12590
1/84
FHF complex
GO:0070695
2/12590
1/84
endoplasmic reticulum−endosome membrane contact site
GO:0140284
2/12590
1/84
cytosolic small ribosomal subunit
GO:0022627
40/12590
3/84
axolemma
GO:0030673
5/12590
1/84
molecular_function
voltage−gated potassium channel activity
GO:0005249
14/12590
2/84
translation regulator activity
GO:0045182
14/12590
2/84
structural constituent of ribosome
GO:0003735
179/12590
10/84
soluble NSF attachment protein activity
GO:0005483
2/12590
1/84
Rab GDP−dissociation inhibitor activity
GO:0005093
2/12590
1/84
protein kinase activity
GO:0004672
521/12590
9/84
mRNA 5'−UTR binding
GO:0048027
12/12590
2/84
extracellular matrix structural constituent
GO:0005201
26/12590
3/84
deoxyribonuclease II activity
GO:0004531
2/12590
1/84
actin filament binding
GO:0051015
69/12590
3/84
0.0% 1.0% 2.0% 3.0% 4.0% 5.0% 6.0% 7.0% 8.0% 9.0% 10.0% 11.0% 12.0%
Enrichment ratios (%)
GO terms classification
Gene set
DE_genes
All_genes

Figure S2b

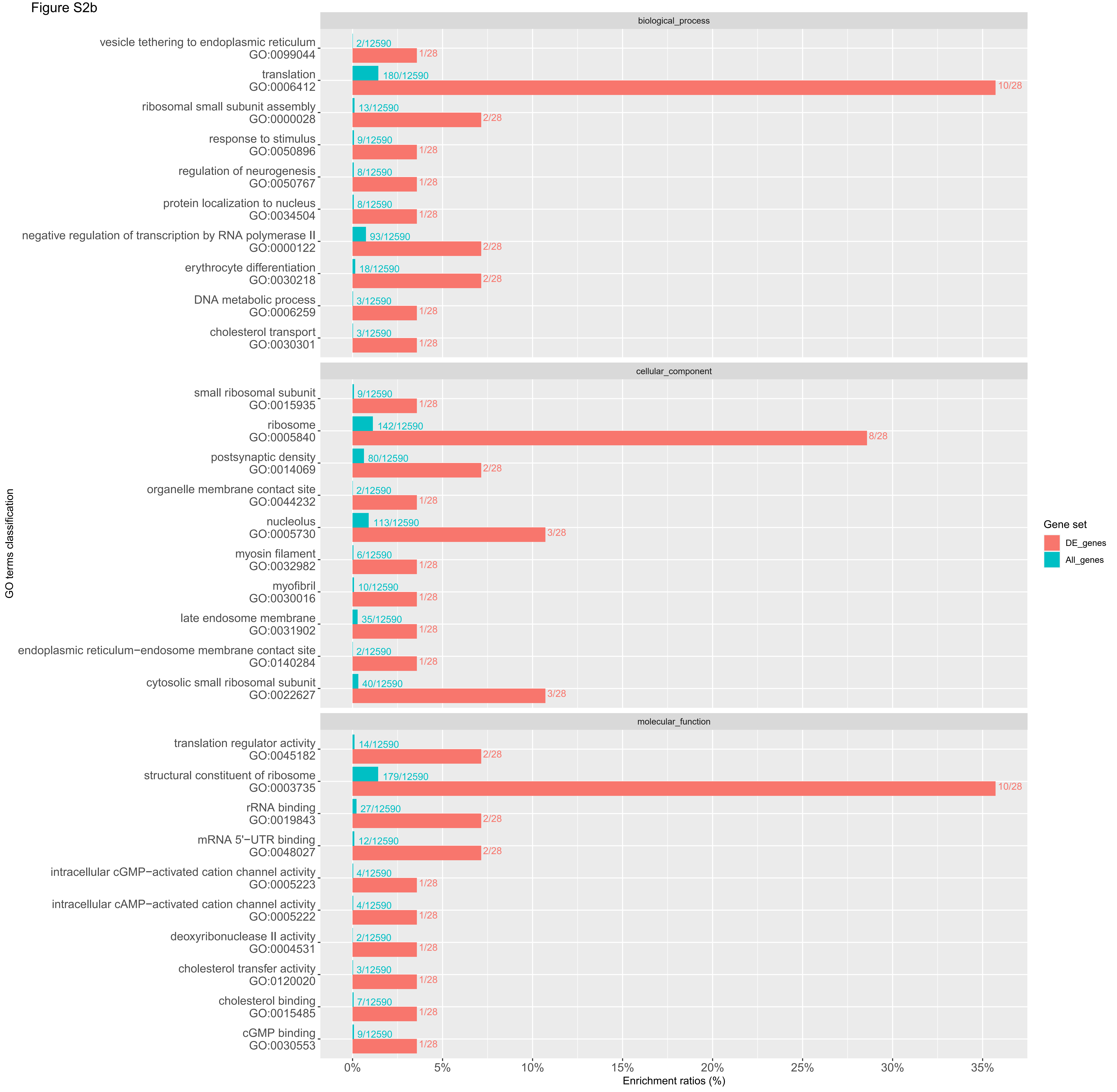

biological_process
vesicle tethering to endoplasmic reticulum GO:0099044
2/12590
1/28
translation GO:0006412
180/12590
10/28
ribosomal small subunit assembly GO:0000028
13/12590
2/28
response to stimulus GO:0050896
9/12590
1/28
regulation of neurogenesis GO:0050767
8/12590
1/28
protein localization to nucleus GO:0034504
8/12590
1/28
negative regulation of transcription by RNA polymerase II GO:0000122
93/12590
2/28
erythrocyte differentiation GO:0030218
18/12590
2/28
DNA metabolic process GO:0006259
3/12590
1/28
cholesterol transport GO:0030301
3/12590
1/28
cellular_component
small ribosomal subunit GO:0015935
9/12590
1/28
ribosome GO:0005840
142/12590
8/28
postsynaptic density GO:0014069
80/12590
2/28
organelle membrane contact site GO:0044232
2/12590
1/28
nucleolus GO:0005730
113/12590
3/28
myosin filament GO:0032982
6/12590
1/28
myofibril GO:0030016
10/12590
1/28
late endosome membrane GO:0031902
35/12590
1/28
endoplasmic reticulum−endosome membrane contact site GO:0140284
2/12590
1/28
cytosolic small ribosomal subunit GO:0022627
40/12590
3/28
molecular_function
translation regulator activity GO:0045182
14/12590
2/28
structural constituent of ribosome GO:0003735
179/12590
10/28
rRNA binding GO:0019843
27/12590
2/28
mRNA 5'−UTR binding GO:0048027
12/12590
2/28
intracellular cGMP−activated cation channel activity GO:0005223
4/12590
1/28
intracellular cAMP−activated cation channel activity GO:0005222
4/12590
1/28
deoxyribonuclease II activity GO:0004531
2/12590
1/28
cholesterol transfer activity GO:0120020
3/12590
1/28
cholesterol binding GO:0015485
7/12590
1/28
cGMP binding GO:0030553
9/12590
1/28
0%
5%
10%
15%
20%
25%
30%
35%
Enrichment ratios (%)
GO terms classification
Gene set
DE_genes
All_genes

Figure S2c

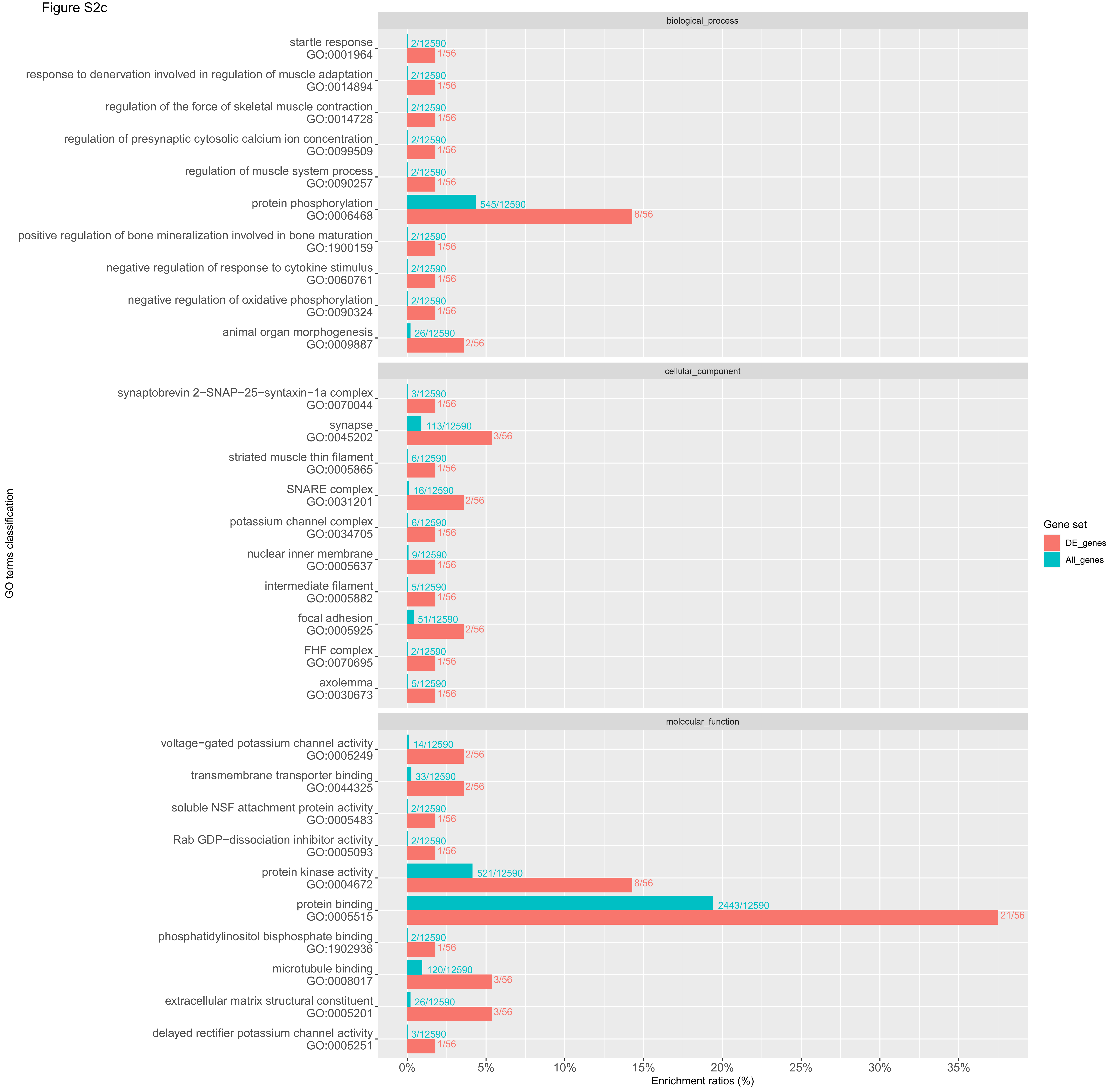
biological_process
startle response
GO:0001964
2/12590
1/56
response to denervation involved in regulation of muscle adaptation
GO:0014894
2/12590
1/56
regulation of the force of skeletal muscle contraction
GO:0014728
2/12590
1/56
regulation of presynaptic cytosolic calcium ion concentration
GO:0099509
2/12590
1/56
regulation of muscle system process
GO:0090257
2/12590
1/56
protein phosphorylation
GO:0006468
545/12590
8/56
positive regulation of bone mineralization involved in bone maturation
GO:1900159
2/12590
1/56
negative regulation of response to cytokine stimulus
GO:0060761
2/12590
1/56
negative regulation of oxidative phosphorylation
GO:0090324
2/12590
1/56
animal organ morphogenesis
GO:0009887
26/12590
2/56
cellular_component
synaptobrevin 2−SNAP−25−syntaxin−1a complex
GO:0070044
3/12590
1/56
synapse
GO:0045202
113/12590
3/56
striated muscle thin filament
GO:0005865
6/12590
1/56
SNARE complex
GO:0031201
16/12590
2/56
potassium channel complex
GO:0034705
6/12590
1/56
nuclear inner membrane
GO:0005637
9/12590
1/56
intermediate filament
GO:0005882
5/12590
1/56
focal adhesion
GO:0005925
51/12590
2/56
FHF complex
GO:0070695
2/12590
1/56
axolemma
GO:0030673
5/12590
1/56
molecular_function
voltage−gated potassium channel activity
GO:0005249
14/12590
2/56
transmembrane transporter binding
GO:0044325
33/12590
2/56
soluble NSF attachment protein activity
GO:0005483
2/12590
1/56
Rab GDP−dissociation inhibitor activity
GO:0005093
2/12590
1/56
protein kinase activity
GO:0004672
521/12590
8/56
protein binding
GO:0005515
2443/12590
21/56
phosphatidylinositol bisphosphate binding
GO:1902936
2/12590
1/56
microtubule binding
GO:0008017
120/12590
3/56
extracellular matrix structural constituent
GO:0005201
26/12590
3/56
delayed rectifier potassium channel activity
GO:0005251
3/12590
1/56
GO terms classification
0%
5%
10%
15%
20%
25%
30%
35%
Enrichment ratios (%)
Gene set
DE_genes
All_genes

Figure S3a

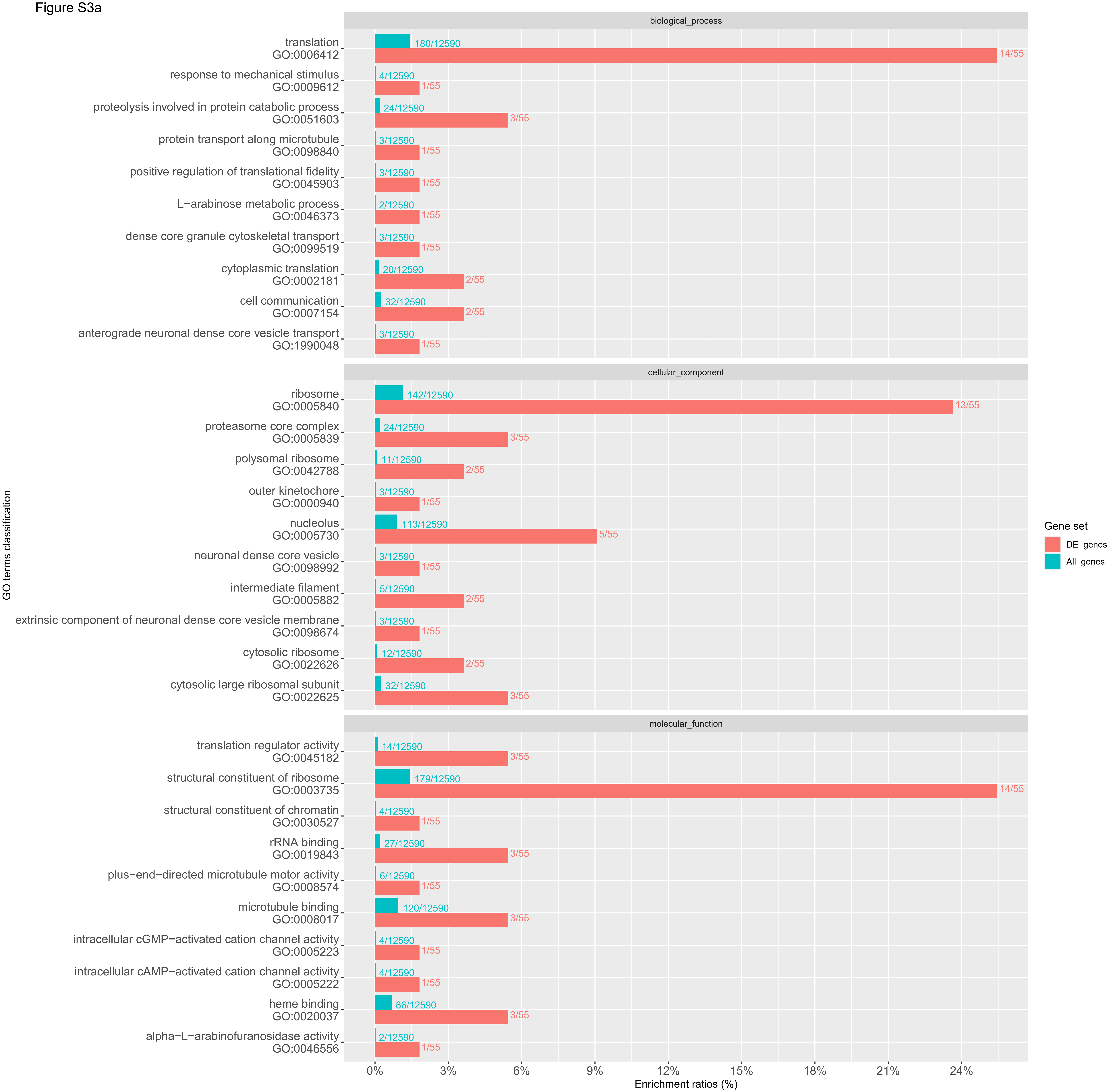

biological_process
translation
GO:0006412
180/12590
14/55
response to mechanical stimulus
GO:0009612
4/12590
1/55
proteolysis involved in protein catabolic process
GO:0051603
24/12590
3/55
protein transport along microtubule
GO:0098840
3/12590
1/55
positive regulation of translational fidelity
GO:0045903
3/12590
1/55
L−arabinose metabolic process
GO:0046373
2/12590
1/55
dense core granule cytoskeletal transport
GO:0099519
3/12590
1/55
cytoplasmic translation
GO:0002181
20/12590
2/55
cell communication
GO:0007154
32/12590
2/55
anterograde neuronal dense core vesicle transport
GO:1990048
3/12590
1/55
cellular_component
ribosome
GO:0005840
142/12590
13/55
proteasome core complex
GO:0005839
24/12590
3/55
polysomal ribosome
GO:0042788
11/12590
2/55
outer kinetochore
GO:0000940
3/12590
1/55
nucleolus
GO:0005730
113/12590
5/55
neuronal dense core vesicle
GO:0098992
3/12590
1/55
intermediate filament
GO:0005882
5/12590
2/55
extrinsic component of neuronal dense core vesicle membrane
GO:0098674
3/12590
1/55
cytosolic ribosome
GO:0022626
12/12590
2/55
cytosolic large ribosomal subunit
GO:0022625
32/12590
3/55
molecular_function
translation regulator activity
GO:0045182
14/12590
3/55
structural constituent of ribosome
GO:0003735
179/12590
14/55
structural constituent of chromatin
GO:0030527
4/12590
1/55
rRNA binding
GO:0019843
27/12590
3/55
plus−end−directed microtubule motor activity
GO:0008574
6/12590
1/55
microtubule binding
GO:0008017
120/12590
3/55
intracellular cGMP−activated cation channel activity
GO:0005223
4/12590
1/55
intracellular cAMP−activated cation channel activity
GO:0005222
4/12590
1/55
heme binding
GO:0020037
86/12590
3/55
alpha−L−arabinofuranosidase activity
GO:0046556
2/12590
1/55
GO terms classification
0%
3%
6%
9%
12%
15%
18%
21%
24%
Enrichment ratios (%)
Gene set
DE_genes
All_genes

Figure S3b

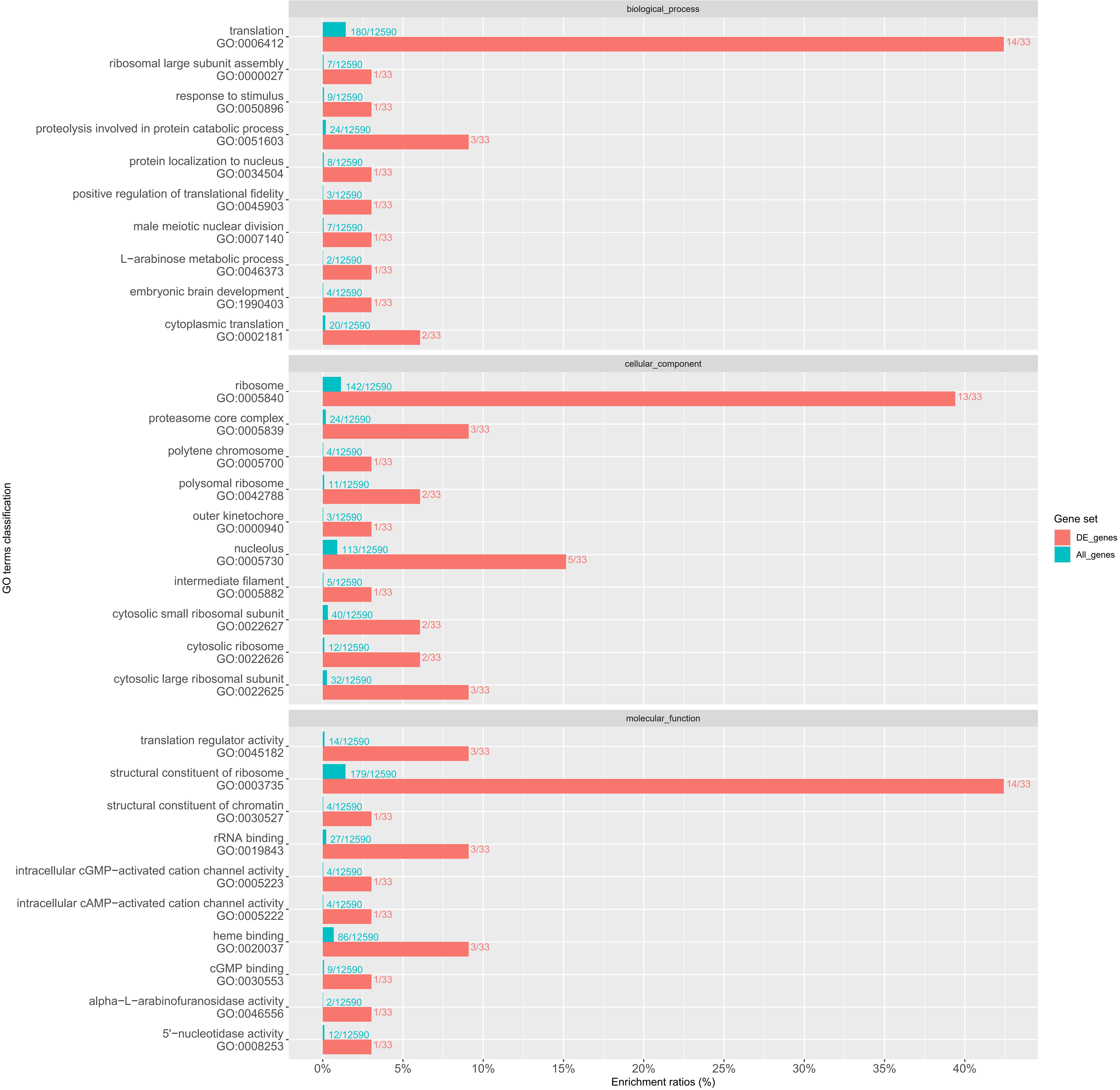
biological_process
translation
GO:0006412
180/12590
14/33
ribosomal large subunit assembly
GO:0000027
7/12590
1/33
response to stimulus
GO:0050896
9/12590
1/33
proteolysis involved in protein catabolic process
GO:0051603
24/12590
3/33
protein localization to nucleus
GO:0034504
8/12590
1/33
positive regulation of translational fidelity
GO:0045903
3/12590
1/33
male meiotic nuclear division
GO:0007140
7/12590
1/33
L−arabinose metabolic process
GO:0046373
2/12590
1/33
embryonic brain development
GO:1990403
4/12590
1/33
cytoplasmic translation
GO:0002181
20/12590
2/33
cellular_component
ribosome
GO:0005840
142/12590
13/33
proteasome core complex
GO:0005839
24/12590
3/33
polytene chromosome
GO:0005700
4/12590
1/33
polysomal ribosome
GO:0042788
11/12590
2/33
outer kinetochore
GO:0000940
3/12590
1/33
nucleolus
GO:0005730
113/12590
5/33
intermediate filament
GO:0005882
5/12590
1/33
cytosolic small ribosomal subunit
GO:0022627
40/12590
2/33
cytosolic ribosome
GO:0022626
12/12590
2/33
cytosolic large ribosomal subunit
GO:0022625
32/12590
3/33
molecular_function
translation regulator activity
GO:0045182
14/12590
3/33
structural constituent of ribosome
GO:0003735
179/12590
14/33
structural constituent of chromatin
GO:0030527
4/12590
1/33
rRNA binding
GO:0019843
27/12590
3/33
intracellular cGMP−activated cation channel activity
GO:0005223
4/12590
1/33
intracellular cAMP−activated cation channel activity
GO:0005222
4/12590
1/33
heme binding
GO:0020037
86/12590
3/33
cGMP binding
GO:0030553
9/12590
1/33
alpha−L−arabinofuranosidase activity
GO:0046556
2/12590
1/33
5'−nucleotidase activity
GO:0008253
12/12590
1/33
GO terms classification
0%
5%
10%
15%
20%
25%
30%
35%
40%
Enrichment ratios (%)
Gene set
DE_genes
All_genes

Figure S3c

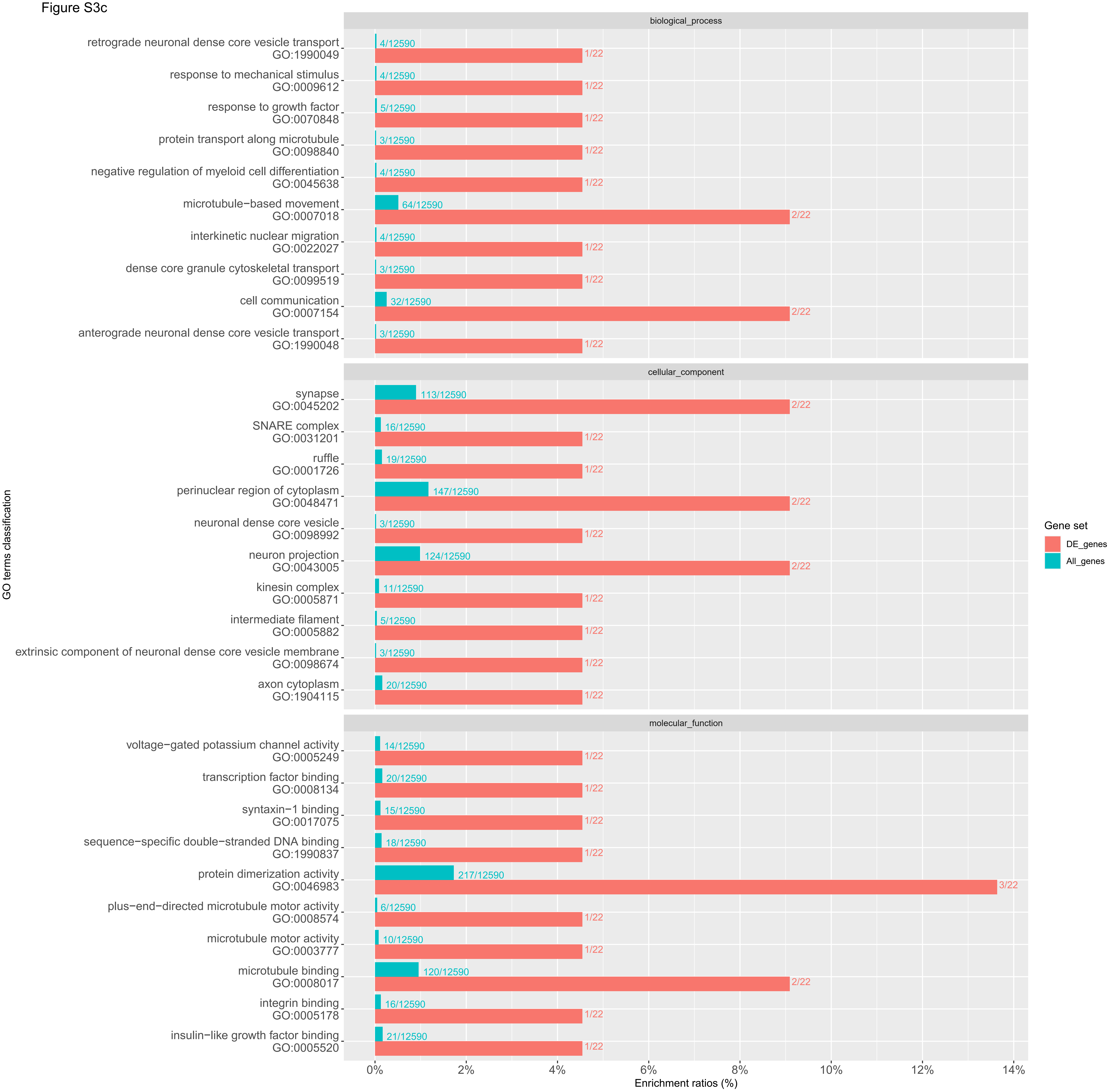
biological_process
retrograde neuronal dense core vesicle transport
GO:1990049
4/12590
1/22
response to mechanical stimulus
GO:0009612
4/12590
1/22
response to growth factor
GO:0070848
5/12590
1/22
protein transport along microtubule
GO:0098840
3/12590
1/22
negative regulation of myeloid cell differentiation
GO:0045638
4/12590
1/22
microtubule−based movement
GO:0007018
64/12590
2/22
interkinetic nuclear migration
GO:0022027
4/12590
1/22
dense core granule cytoskeletal transport
GO:0099519
3/12590
1/22
cell communication
GO:0007154
32/12590
2/22
anterograde neuronal dense core vesicle transport
GO:1990048
3/12590
1/22
cellular_component
synapse
GO:0045202
113/12590
2/22
SNARE complex
GO:0031201
16/12590
1/22
ruffle
GO:0001726
19/12590
1/22
perinuclear region of cytoplasm
GO:0048471
147/12590
2/22
neuronal dense core vesicle
GO:0098992
3/12590
1/22
neuron projection
GO:0043005
124/12590
2/22
kinesin complex
GO:0005871
11/12590
1/22
intermediate filament
GO:0005882
5/12590
1/22
extrinsic component of neuronal dense core vesicle membrane
GO:0098674
3/12590
1/22
axon cytoplasm
GO:1904115
20/12590
1/22
molecular_function
voltage−gated potassium channel activity
GO:0005249
14/12590
1/22
transcription factor binding
GO:0008134
20/12590
1/22
syntaxin−1 binding
GO:0017075
15/12590
1/22
sequence−specific double−stranded DNA binding
GO:1990837
18/12590
1/22
protein dimerization activity
GO:0046983
217/12590
3/22
plus−end−directed microtubule motor activity
GO:0008574
6/12590
1/22
microtubule motor activity
GO:0003777
10/12590
1/22
microtubule binding
GO:0008017
120/12590
2/22
integrin binding
GO:0005178
16/12590
1/22
insulin−like growth factor binding
GO:0005520
21/12590
1/22
GO terms classification
0%
2%
4%
6%
8%
10%
12%
14%
Enrichment ratios (%)
Gene set
DE_genes
All_genes

Figure S4a

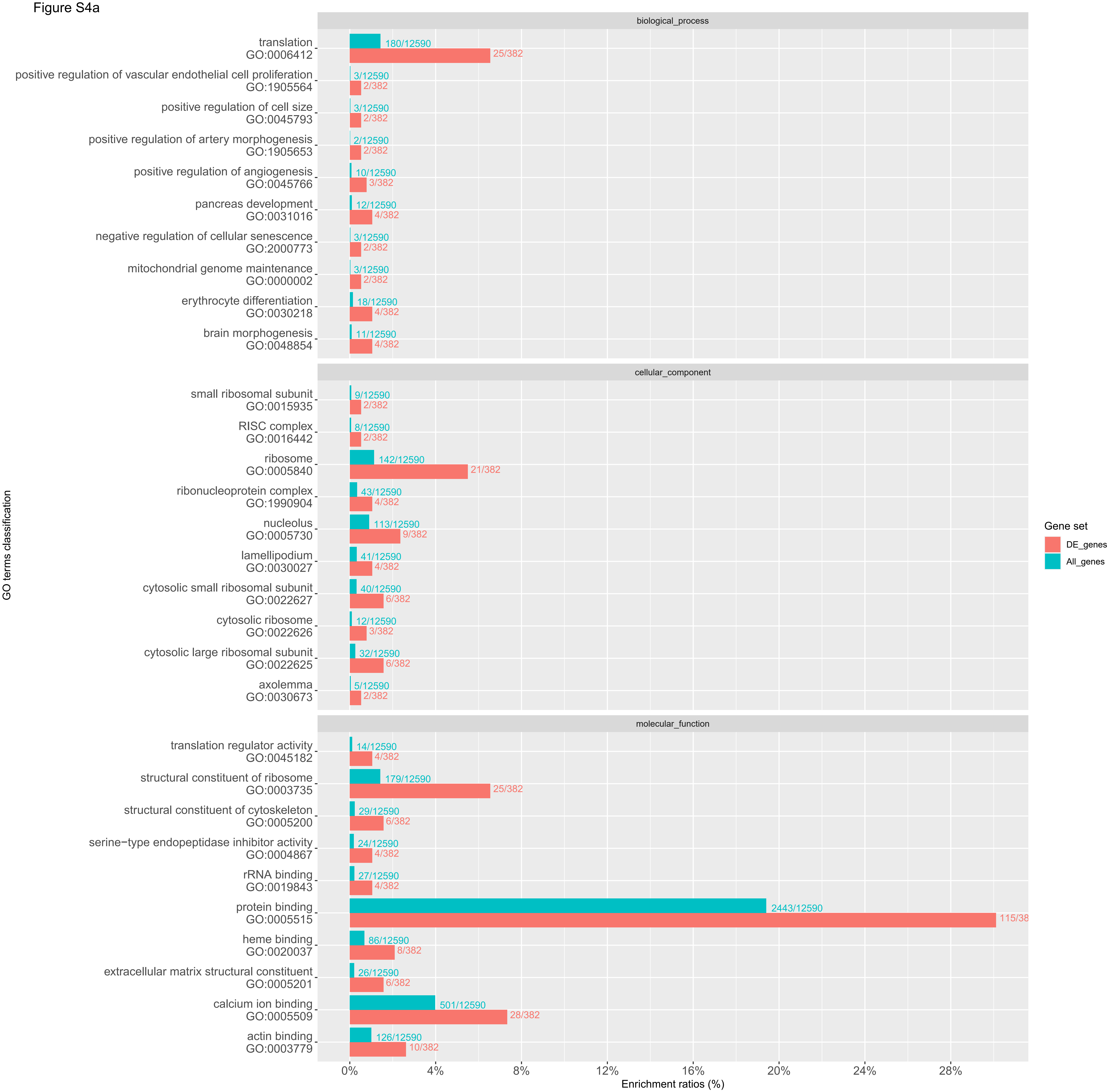

biological_process
translation
GO:0006412
180/12590
25/382
positive regulation of vascular endothelial cell proliferation
GO:1905564
3/12590
2/382
positive regulation of cell size
GO:0045793
3/12590
2/382
positive regulation of artery morphogenesis
GO:1905653
2/12590
2/382
positive regulation of angiogenesis
GO:0045766
10/12590
3/382
pancreas development
GO:0031016
12/12590
4/382
negative regulation of cellular senescence
GO:2000773
3/12590
2/382
mitochondrial genome maintenance
GO:0000002
3/12590
2/382
erythrocyte differentiation
GO:0030218
18/12590
4/382
brain morphogenesis
GO:0048854
11/12590
4/382
cellular_component
small ribosomal subunit
GO:0015935
9/12590
2/382
RISC complex
GO:0016442
8/12590
2/382
ribosome
GO:0005840
142/12590
21/382
ribonucleoprotein complex
GO:1990904
43/12590
4/382
nucleolus
GO:0005730
113/12590
9/382
lamellipodium
GO:0030027
41/12590
4/382
cytosolic small ribosomal subunit
GO:0022627
40/12590
6/382
cytosolic ribosome
GO:0022626
12/12590
3/382
cytosolic large ribosomal subunit
GO:0022625
32/12590
6/382
axolemma
GO:0030673
5/12590
2/382
molecular_function
translation regulator activity
GO:0045182
14/12590
4/382
structural constituent of ribosome
GO:0003735
179/12590
25/382
structural constituent of cytoskeleton
GO:0005200
29/12590
6/382
serine−type endopeptidase inhibitor activity
GO:0004867
24/12590
4/382
rRNA binding
GO:0019843
27/12590
4/382
protein binding
GO:0005515
2443/12590
115/38
heme binding
GO:0020037
86/12590
8/382
extracellular matrix structural constituent
GO:0005201
26/12590
6/382
calcium ion binding
GO:0005509
501/12590
28/382
actin binding
GO:0003779
126/12590
10/382
0%
4%
8%
12%
16%
20%
24%
28%
Enrichment ratios (%)
GO terms classification
Gene set
DE_genes
All_genes

Figure S4b

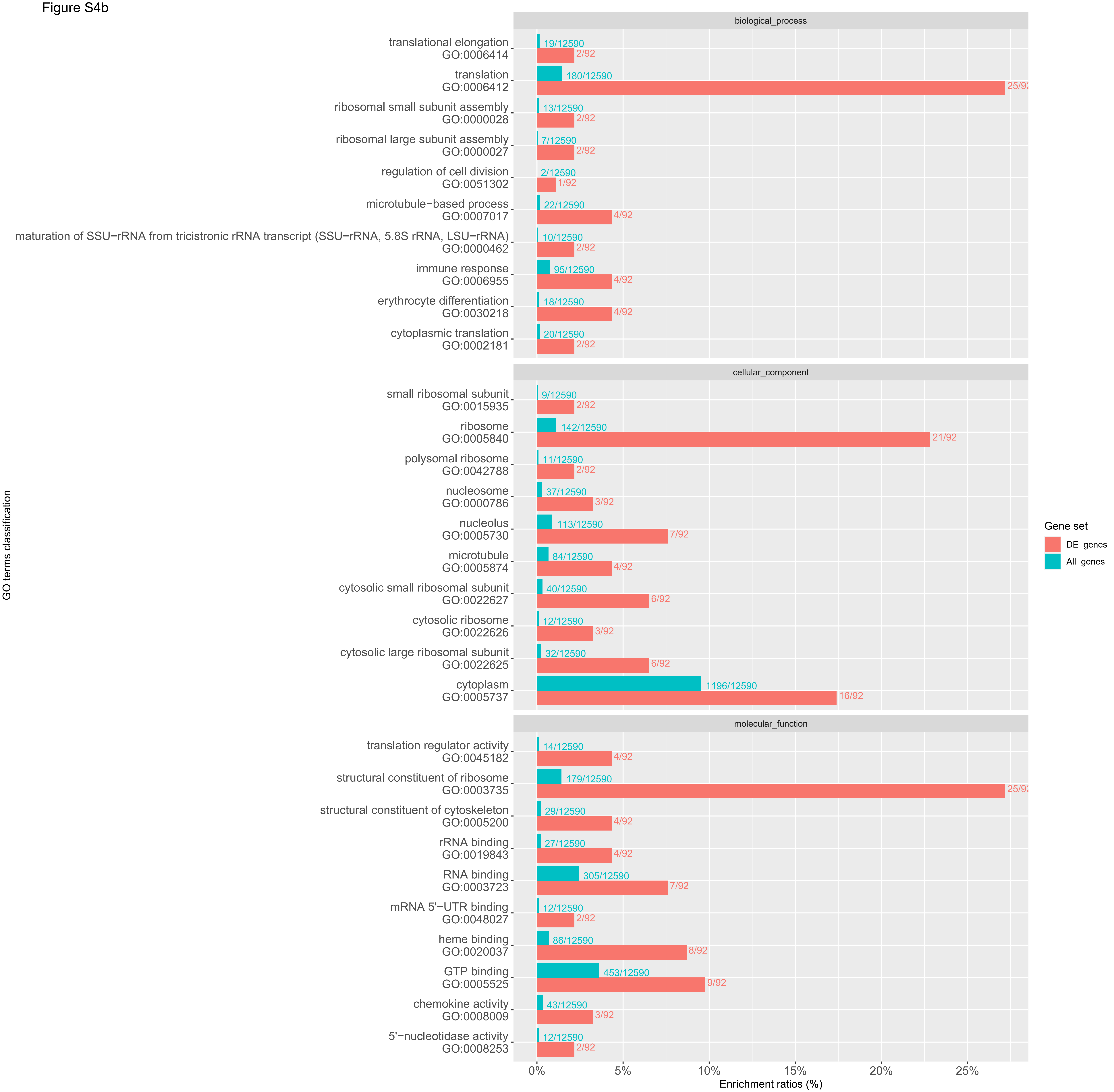

biological_process
translational elongation
GO:0006414
19/12590
2/92
translation
GO:0006412
180/12590
25/92
ribosomal small subunit assembly
GO:0000028
13/12590
2/92
ribosomal large subunit assembly
GO:0000027
7/12590
2/92
regulation of cell division
GO:0051302
2/12590
1/92
microtubule−based process
GO:0007017
22/12590
4/92
maturation of SSU−rRNA from tricistronic rRNA transcript (SSU−rRNA, 5.8S rRNA, LSU−rRNA)
GO:0000462
10/12590
2/92
immune response
GO:0006955
95/12590
4/92
erythrocyte differentiation
GO:0030218
18/12590
4/92
cytoplasmic translation
GO:0002181
20/12590
2/92
cellular_component
small ribosomal subunit
GO:0015935
9/12590
2/92
ribosome
GO:0005840
142/12590
21/92
polysomal ribosome
GO:0042788
11/12590
2/92
nucleosome
GO:0000786
37/12590
3/92
nucleolus
GO:0005730
113/12590
7/92
microtubule
GO:0005874
84/12590
4/92
cytosolic small ribosomal subunit
GO:0022627
40/12590
6/92
cytosolic ribosome
GO:0022626
12/12590
3/92
cytosolic large ribosomal subunit
GO:0022625
32/12590
6/92
cytoplasm
GO:0005737
1196/12590
16/92
molecular_function
translation regulator activity
GO:0045182
14/12590
4/92
structural constituent of ribosome
GO:0003735
179/12590
25/92
structural constituent of cytoskeleton
GO:0005200
29/12590
4/92
rRNA binding
GO:0019843
27/12590
4/92
RNA binding
GO:0003723
305/12590
7/92
mRNA 5'−UTR binding
GO:0048027
12/12590
2/92
heme binding
GO:0020037
86/12590
8/92
GTP binding
GO:0005525
453/12590
9/92
chemokine activity
GO:0008009
43/12590
3/92
5'−nucleotidase activity
GO:0008253
12/12590
2/92
GO terms classification
0%
5%
10%
15%
20%
25%
Enrichment ratios (%)
Gene set
DE_genes
All_genes

Figure S4c

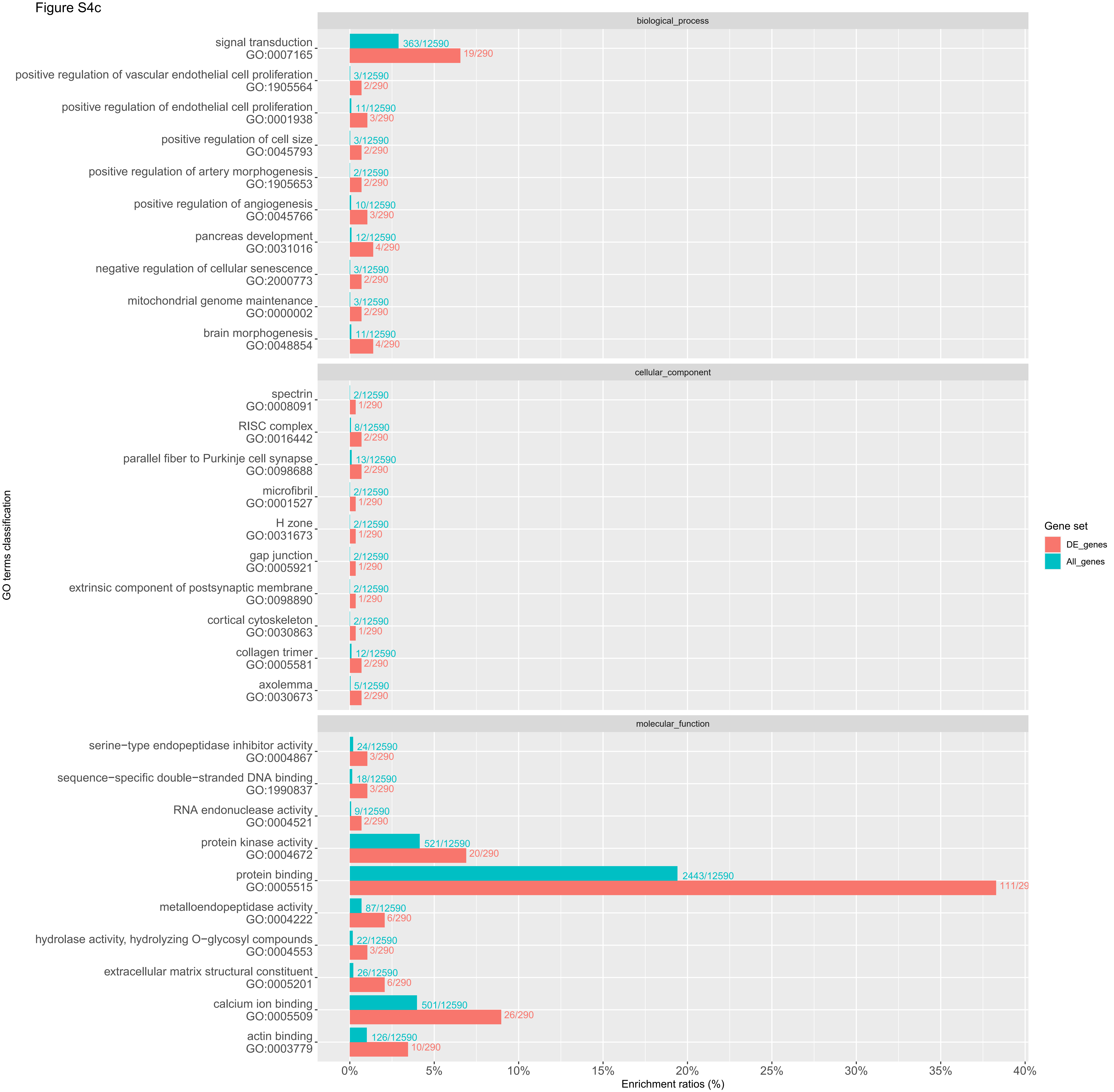

biological_process
signal transduction GO:0007165
363/12590
19/290
positive regulation of vascular endothelial cell proliferation GO:1905564
3/12590
2/290
positive regulation of endothelial cell proliferation GO:0001938
11/12590
3/290
positive regulation of cell size GO:0045793
3/12590
2/290
positive regulation of artery morphogenesis GO:1905653
2/12590
2/290
positive regulation of angiogenesis GO:0045766
10/12590
3/290
pancreas development GO:0031016
12/12590
4/290
negative regulation of cellular senescence GO:2000773
3/12590
2/290
mitochondrial genome maintenance GO:0000002
3/12590
2/290
brain morphogenesis GO:0048854
11/12590
4/290
cellular_component
spectrin GO:0008091
2/12590
1/290
RISC complex GO:0016442
8/12590
2/290
parallel fiber to Purkinje cell synapse GO:0098688
13/12590
2/290
microfibril GO:0001527
2/12590
1/290
H zone GO:0031673
2/12590
1/290
gap junction GO:0005921
2/12590
1/290
extrinsic component of postsynaptic membrane GO:0098890
2/12590
1/290
cortical cytoskeleton GO:0030863
2/12590
1/290
collagen trimer GO:0005581
12/12590
2/290
axolemma GO:0030673
5/12590
2/290
molecular_function
serine−type endopeptidase inhibitor activity GO:0004867
24/12590
3/290
sequence−specific double−stranded DNA binding GO:1990837
18/12590
3/290
RNA endonuclease activity GO:0004521
9/12590
2/290
protein kinase activity GO:0004672
521/12590
20/290
protein binding GO:0005515
2443/12590
111/29
metalloendopeptidase activity GO:0004222
87/12590
6/290
hydrolase activity, hydrolyzing O−glycosyl compounds GO:0004553
22/12590
3/290
extracellular matrix structural constituent GO:0005201
26/12590
6/290
calcium ion binding GO:0005509
501/12590
26/290
actin binding GO:0003779
126/12590
10/290
GO terms classification
0%
5%
10%
15%
20%
25%
30%
35%
40%
Enrichment ratios (%)
Gene set
DE_genes
All_genes

Figure S5a

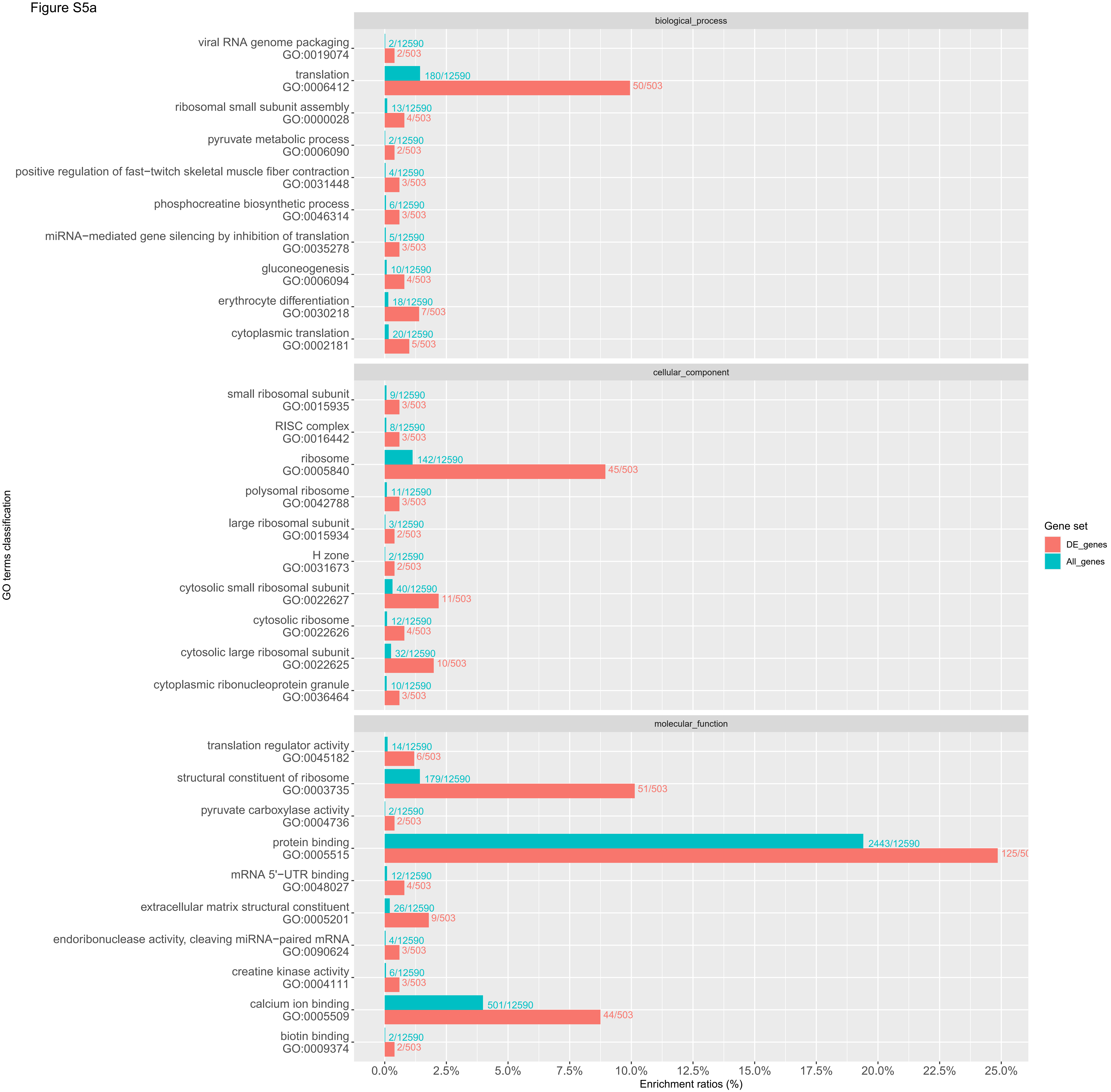

biological_process
viral RNA genome packaging GO:0019074
2/12590
2/503
translation GO:0006412
180/12590
50/503
ribosomal small subunit assembly GO:0000028
13/12590
4/503
pyruvate metabolic process GO:0006090
2/12590
2/503
positive regulation of fast−twitch skeletal muscle fiber contraction GO:0031448
4/12590
3/503
phosphocreatine biosynthetic process GO:0046314
6/12590
3/503
miRNA−mediated gene silencing by inhibition of translation GO:0035278
5/12590
3/503
gluconeogenesis GO:0006094
10/12590
4/503
erythrocyte differentiation GO:0030218
18/12590
7/503
cytoplasmic translation GO:0002181
20/12590
5/503
cellular_component
small ribosomal subunit GO:0015935
9/12590
3/503
RISC complex GO:0016442
8/12590
3/503
ribosome GO:0005840
142/12590
45/503
polysomal ribosome GO:0042788
11/12590
3/503
large ribosomal subunit GO:0015934
3/12590
2/503
H zone GO:0031673
2/12590
2/503
cytosolic small ribosomal subunit GO:0022627
40/12590
11/503
cytosolic ribosome GO:0022626
12/12590
4/503
cytosolic large ribosomal subunit GO:0022625
32/12590
10/503
cytoplasmic ribonucleoprotein granule GO:0036464
10/12590
3/503
molecular_function
translation regulator activity GO:0045182
14/12590
6/503
structural constituent of ribosome GO:0003735
179/12590
51/503
pyruvate carboxylase activity GO:0004736
2/12590
2/503
protein binding GO:0005515
2443/12590
125/50
mRNA 5'−UTR binding GO:0048027
12/12590
4/503
extracellular matrix structural constituent GO:0005201
26/12590
9/503
endoribonuclease activity, cleaving miRNA−paired mRNA GO:0090624
4/12590
3/503
creatine kinase activity GO:0004111
6/12590
3/503
calcium ion binding GO:0005509
501/12590
44/503
biotin binding GO:0009374
2/12590
2/503
GO terms classification
0.0% 2.5% 5.0% 7.5% 10.0% 12.5% 15.0% 17.5% 20.0% 22.5% 25.0%
Enrichment ratios (%)
Gene set
DE_genes
All_genes

Figure S5b

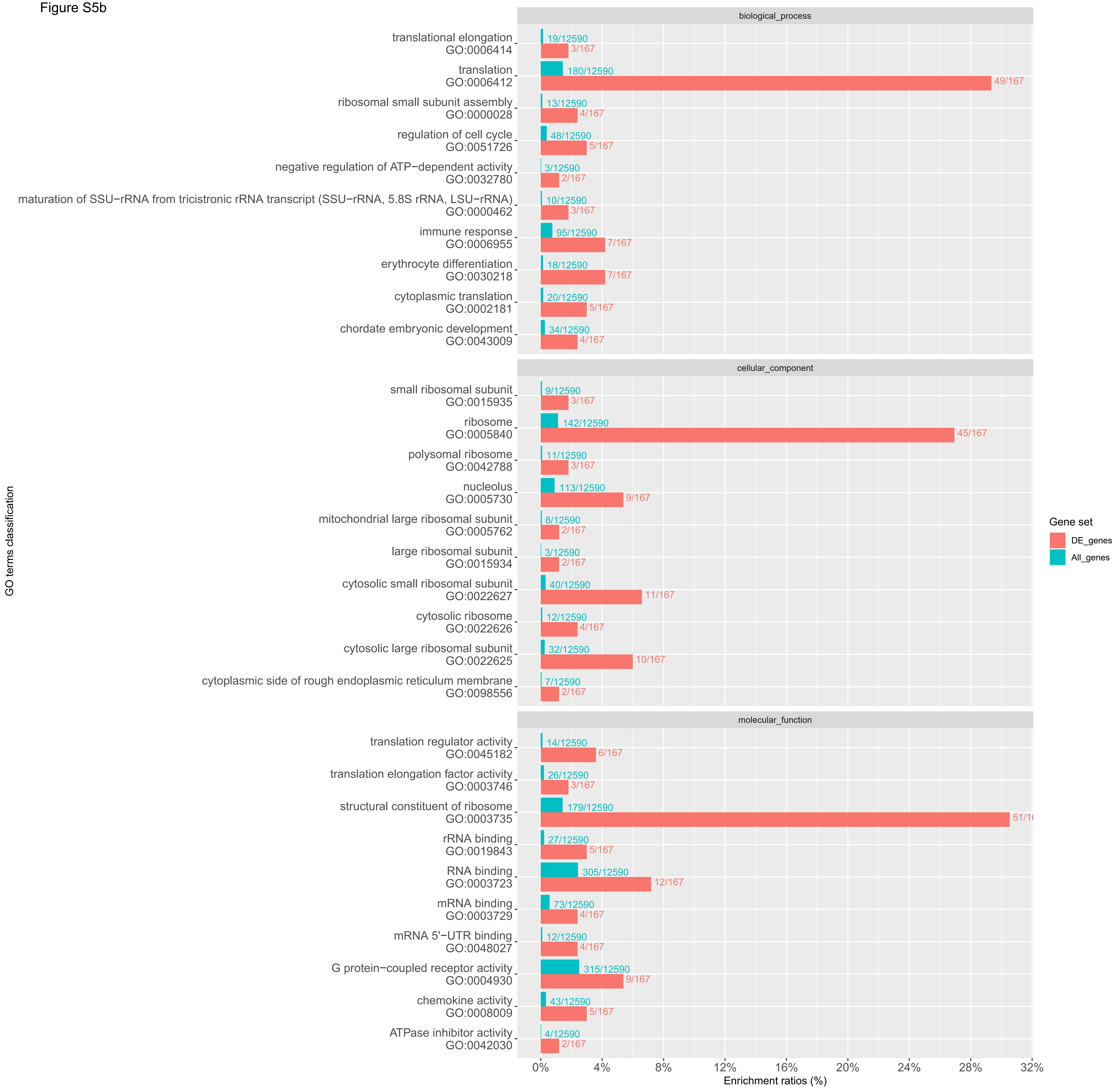
biological_process
translational elongation GO:0006414
19/12590
3/167
translation GO:0006412
180/12590
49/167
ribosomal small subunit assembly GO:0000028
13/12590
4/167
regulation of cell cycle GO:0051726
48/12590
5/167
negative regulation of ATP−dependent activity GO:0032780
3/12590
2/167
maturation of SSU−rRNA from tricistronic rRNA transcript (SSU−rRNA, 5.8S rRNA, LSU−rRNA) GO:0000462
10/12590
3/167
immune response GO:0006955
95/12590
7/167
erythrocyte differentiation GO:0030218
18/12590
7/167
cytoplasmic translation GO:0002181
20/12590
5/167
chordate embryonic development GO:0043009
34/12590
4/167
cellular_component
small ribosomal subunit GO:0015935
9/12590
3/167
ribosome GO:0005840
142/12590
45/167
polysomal ribosome GO:0042788
11/12590
3/167
nucleolus GO:0005730
113/12590
9/167
mitochondrial large ribosomal subunit GO:0005762
8/12590
2/167
large ribosomal subunit GO:0015934
3/12590
2/167
cytosolic small ribosomal subunit GO:0022627
40/12590
11/167
cytosolic ribosome GO:0022626
12/12590
4/167
cytosolic large ribosomal subunit GO:0022625
32/12590
10/167
cytoplasmic side of rough endoplasmic reticulum membrane GO:0098556
7/12590
2/167
molecular_function
translation regulator activity GO:0045182
14/12590
6/167
translation elongation factor activity GO:0003746
26/12590
3/167
structural constituent of ribosome GO:0003735
179/12590
51/1
rRNA binding GO:0019843
27/12590
5/167
RNA binding GO:0003723
305/12590
12/167
mRNA binding GO:0003729
73/12590
4/167
mRNA 5'−UTR binding GO:0048027
12/12590
4/167
G protein−coupled receptor activity GO:0004930
315/12590
9/167
chemokine activity GO:0008009
43/12590
5/167
ATPase inhibitor activity GO:0042030
4/12590
2/167
GO terms classification
0%
4%
8%
12%
16%
20%
24%
28%
32%
Enrichment ratios (%)
Gene set
DE_genes
All_genes

Figure S5c

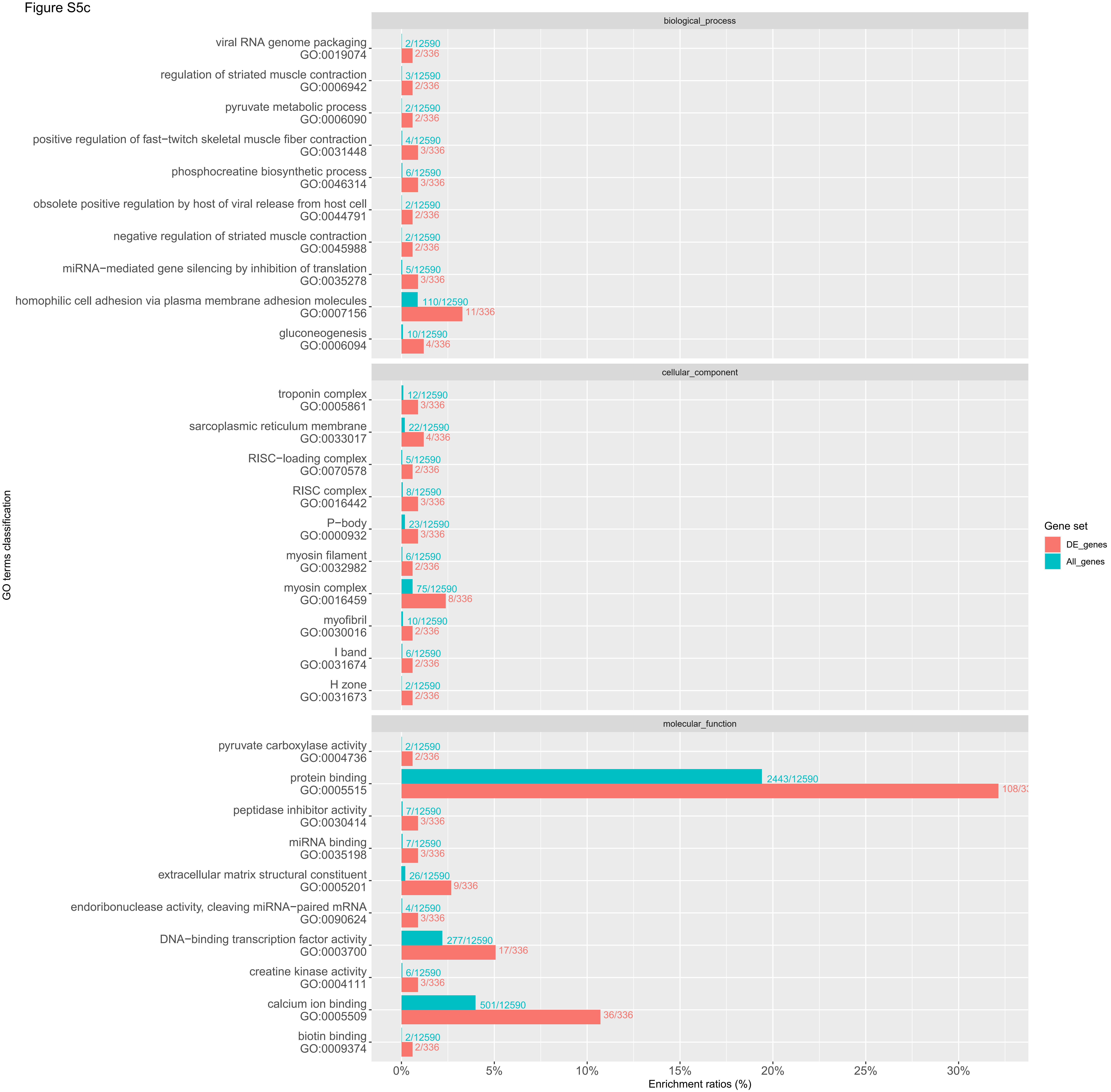

biological_process
viral RNA genome packaging GO:0019074
2/12590
2/336
regulation of striated muscle contraction GO:0006942
3/12590
2/336
pyruvate metabolic process GO:0006090
2/12590
2/336
positive regulation of fast−twitch skeletal muscle fiber contraction GO:0031448
4/12590
3/336
phosphocreatine biosynthetic process GO:0046314
6/12590
3/336
obsolete positive regulation by host of viral release from host cell GO:0044791
2/12590
2/336
negative regulation of striated muscle contraction GO:0045988
2/12590
2/336
miRNA−mediated gene silencing by inhibition of translation GO:0035278
5/12590
3/336
homophilic cell adhesion via plasma membrane adhesion molecules GO:0007156
110/12590
11/336
gluconeogenesis GO:0006094
10/12590
4/336
cellular_component
troponin complex GO:0005861
12/12590
3/336
sarcoplasmic reticulum membrane GO:0033017
22/12590
4/336
RISC−loading complex GO:0070578
5/12590
2/336
RISC complex GO:0016442
8/12590
3/336
P−body GO:0000932
23/12590
3/336
myosin filament GO:0032982
6/12590
2/336
myosin complex GO:0016459
75/12590
8/336
myofibril GO:0030016
10/12590
2/336
I band GO:0031674
6/12590
2/336
H zone GO:0031673
2/12590
2/336
molecular_function
pyruvate carboxylase activity GO:0004736
2/12590
2/336
protein binding GO:0005515
2443/12590
108/33
peptidase inhibitor activity GO:0030414
7/12590
3/336
miRNA binding GO:0035198
7/12590
3/336
extracellular matrix structural constituent GO:0005201
26/12590
9/336
endoribonuclease activity, cleaving miRNA−paired mRNA GO:0090624
4/12590
3/336
DNA−binding transcription factor activity GO:0003700
277/12590
17/336
creatine kinase activity GO:0004111
6/12590
3/336
calcium ion binding GO:0005509
501/12590
36/336
biotin binding GO:0009374
2/12590
2/336
0%
5%
10%
15%
20%
25%
30%
Enrichment ratios (%)
GO terms classification
Gene set
DE_genes
All_genes

Supplementary material

File S1: RNA-seq data primary analysis details

A quality check of raw paired-end fastq reads was carried out by FastQC, and a contamination check against human (GRCh38), mouse (GRCm38), yeast (S. cerevisiae R64-1-1), E. coli BL21(DE3) and other organisms by BioBloom tools.

The quality and Illumina adapter trimming of raw reads was performed using Trimmomatic v0.39 (options: PE CROP:250 LEADING:3 TRAILING:3 SLIDINGWINDOW:4:5 ILLUMINACLIP:adapters.fa:2:30:10:3:true MINLEN:35).

Trimmed reads were aligned to the reference transcriptome using STAR v2.7.3a with options: --runMode alignReads --sjdbOverhang 100 --outFilterMultimapNmax 20 --alignSJoverhangMin 8 --alignSJDBoverhangMin 1 --outFilterMismatchNmax 999 --outFilterMismatchNoverReadLmax 1.0 --outFilterMismatchNoverLmax 0.1 --alignIntronMin 20 --alignIntronMax 1000000 --alignMatesGapMax 1000000 --outFilterMatchNmin 0 --outFilterScoreMinOverLread 0.66 --outFilterMatchNminOverLread 0.66 --outSAMheaderHD @HD VN:1.4 SO:coordinate --peOverlapMMp 0.1 --chimOutJunctionFormat 1 --chimSegmentMin 12 --chimJunctionOverhangMin 12 --chimOutType Junctions SeparateSAMold --outSAMunmapped Within --outFilterType BySJout --outSAMattributes All --quantMode GeneCounts TranscriptomeSAM --sjdbScore 1 --twopassMode Basic --outMultimapperOrder Random --outSAMtype BAM SortedByCoordinate

The mapped reads were deduplicated by Picard’s MarkDuplicates v2.27.1 with options: -REMOVE_DUPLICATES False -ASSUME_SORTED true -PROGRAM_RECORD_ID null -VALIDATION_STRINGENCY LENIENT

The quantification of gene expression was performed by Subread's featureCounts v1.6.4 with options: -t exon -g gene_id -p -P -C -s 0 -T 10 -F GTF -Q 0 -d 1 -D 25000

Table S1: The results of the alignment to *Cyprinus carpio* genome

Number of reads per sample categorized right after the alignment. The amount of reads overlapping genes ranges from 23.69% to 27.54%, with 26.12% as the average.

Category	Overlapping Genes	Overlapping Genes (%)	No Feature	Ambiguous Features	Multimapping	Unmapped	Total
K_AB_rep1	3630742	25.95	533900	306115	1714729	7805011	13990497
K_AB_rep2	3043134	26.14	422677	238237	1402555	6535603	11642206
K_AB_rep3	3452960	25.54	456671	275394	1639246	7692976	13517247
K_AB_rep4	3810051	25.01	581413	281300	1793953	8765369	15232086
K_AB_rep5	3134729	26.14	440285	255483	1489006	6674801	11994304
K_BK_AB_rep1	3635583	25.56	551249	272857	1690170	8072263	14222122
K_BK_AB_rep2	3716263	25.43	561404	294690	1855488	8187154	14614999
K_BK_AB_rep3	3145382	23.69	500342	247010	1516138	7869292	13278164
K_BK_AB_rep4	3297972	24.83	550851	247992	1522023	7665552	13284390
K_BK_AB_rep5	3337701	26.04	418814	271747	1686293	7104532	12819087
K_BK_RR_rep1	4385599	27.09	523804	329587	2044963	8907220	16191173
K_BK_RR_rep2	3515665	27.23	449765	253996	1584764	7105764	12909954
K_BK_RR_rep3	3664553	26.03	519957	271031	1765637	7858668	14079846
K_BK_RR_rep4	3089336	26.62	360546	237770	1427951	6488558	11604161
K_BK_RR_rep5	3949860	27.04	551801	279336	1777960	8049053	14608010
K_F1_ABRR_rep1	3036489	25.95	384041	232631	1524444	6522648	11700253
K_F1_ABRR_rep2	3094838	26.60	371974	242962	1530329	6395630	11635733
K_F1_ABRR_rep3	3264895	25.26	452746	258122	1606796	7344013	12926572
K_F1_ABRR_rep4	3710930	24.02	690328	243062	1738396	9063657	15446373
K_F1_ABRR_rep5	3651039	24.54	600781	253082	1697621	8676905	14879428
K_RR_rep1	4025318	26.71	483328	297503	1960433	8304226	15070808
K_RR_rep3	3408449	25.00	500522	237293	1546298	7939958	13632520
K_RR_rep4	4147983	26.81	501990	330527	2004806	8487899	15473205
K_RR_rep5	3121290	27.47	360858	240776	1456454	6184263	11363641
OK_AB_rep1	3725697	26.98	458845	303617	1770272	7552752	13811183
OK_AB_rep2	3191201	25.85	399682	249454	1526670	6976418	12343425
OK_AB_rep3	3525349	27.09	408170	281184	1681964	7115959	13012626
OK_AB_rep4	3195724	25.21	400117	220567	1435409	7422906	12674723
OK_AB_rep5	3194009	26.12	406686	243843	1535177	6846655	12226370

OK_BK_AB_rep1	3492896	26.52	436931	252087	1559707	7429400	13171021
OK_BK_AB_rep2	3308444	27.01	415792	258006	1549048	6718342	12249632
OK_BK_AB_rep3	3430499	27.24	412780	291188	1852807	6605557	12592831
OK_BK_AB_rep4	3345079	26.33	437439	256330	1569425	7097906	12706179
OK_BK_AB_rep5	3364282	27.04	435376	265243	1610469	6766073	12441443
OK_BK_RR_rep1	3392363	25.28	382605	266571	1625572	7754650	13421761
OK_BK_RR_rep2	3559744	27.54	435727	291859	1723350	6916951	12927631
OK_BK_RR_rep3	3369884	26.48	472846	229481	1495847	7159729	12727787
OK_BK_RR_rep5	2939879	25.53	398358	221613	1418468	6534857	11513175
OK_F1_ABRR_rep1	2017541	25.92	232801	168482	1001309	4362396	7782529
OK_F1_ABRR_rep3	2925433	26.12	335952	234174	1452050	6251745	11199354
OK_F1_ABRR_rep4	2921465	25.91	356132	218383	1374372	6405219	11275571
OK_F1_ABRR_rep5	3570497	25.71	481380	265330	1615345	7952644	13885196
OK_RR_rep1	2999324	26.35	343987	253084	1496887	6287882	11381164
OK_RR_rep3	3576259	27.12	416828	303130	1769300	7121811	13187328
OK_RR_rep4	3743058	27.27	468135	275167	1692894	7547508	13726762
OK_RR_rep5	3515604	26.13	471657	258683	1629535	7577361	13452840

Table S2: The results of the alignment to *Carassius auratus* genome

Number of reads per sample categorized right after the alignment. The amount of reads overlapping genes ranges from 29.98% to 40.46%, with 35.2% as the average.

Category	Overlapping Genes	Overlapping Genes (%)	No Feature	Ambiguous Features	Multimapping	Unmapped	Total
K_AB_rep1	4824460	34.48	278059	131150	1225709	7531117	13990495
K_AB_rep2	4038761	34.69	205557	117097	1003298	6277488	11642201
K_AB_rep3	4596836	34.01	236437	134856	1151567	7397552	13517248
K_AB_rep4	5023207	32.98	308225	139503	1308552	8452594	15232081
K_AB_rep5	4182012	34.87	219554	114629	1055381	6422728	11994304
K_BK_AB_rep1	4649613	32.69	287885	128758	1316429	7839436	14222121
K_BK_AB_rep2	4758723	32.56	333882	121839	1402273	7998282	14614999
K_BK_AB_rep3	3981304	29.98	325855	161493	1230516	7578995	13278163
K_BK_AB_rep4	4181180	31.47	330483	124227	1219881	7428621	13284392
K_BK_AB_rep5	4628861	36.11	199868	120196	1082518	6787643	12819086
K_BK_RR_rep1	5813417	35.90	303328	174479	1449639	8450309	16191172
K_BK_RR_rep2	4586315	35.53	262473	140388	1145409	6775366	12909951
K_BK_RR_rep3	4871138	34.60	284550	132641	1286844	7504676	14079849
K_BK_RR_rep4	4130405	35.59	188442	122563	1000019	6162728	11604157
K_BK_RR_rep5	5124146	35.08	301654	148586	1367042	7666582	14608010
K_F1_ABRR_rep1	4198931	35.89	213548	117021	971920	6198833	11700253
K_F1_ABRR_rep2	4265436	36.66	198099	121581	995296	6055319	11635731
K_F1_ABRR_rep3	4396630	34.01	253067	116554	1111795	7048524	12926570
K_F1_ABRR_rep4	4640735	30.04	421693	131300	1410939	8841706	15446373
K_F1_ABRR_rep5	4666873	31.36	348310	126273	1294581	8443390	14879427
K_RR_rep1	5456978	36.21	295079	163096	1341996	7813653	15070802
K_RR_rep3	4487183	32.92	275168	124421	1126536	7619208	13632516
K_RR_rep4	5647556	36.50	272335	144083	1334531	8074701	15473206
K_RR_rep5	4222996	37.16	185509	115341	1013631	5826164	11363641
OK_AB_rep1	5105381	36.97	221283	144395	1183692	7156429	13811180
OK_AB_rep2	4350096	35.24	189396	122835	1015522	6665572	12343421
OK_AB_rep3	4833452	37.14	187954	128010	1100657	6762550	13012623
OK_AB_rep4	4224550	33.33	178736	131479	1033269	7106681	12674715
OK_AB_rep5	4304723	35.21	196082	129397	1083426	6512741	12226369

OK_BK_AB_rep1	4663822	35.41	204248	123797	1093841	7085311	13171019
OK_BK_AB_rep2	4472804	36.51	206042	117557	1034923	6418299	12249625
OK_BK_AB_rep3	5095133	40.46	179993	113012	1066116	6138576	12592830
OK_BK_AB_rep4	4567828	35.95	204854	119038	1049097	6765359	12706176
OK_BK_AB_rep5	4596562	36.95	199331	135453	1105670	6404423	12441439
OK_BK_RR_rep1	4659000	34.71	197378	147978	1094643	7322759	13421758
OK_BK_RR_rep2	4942964	38.24	200899	130808	1098121	6554838	12927630
OK_BK_RR_rep3	4465329	35.08	217627	113596	1129146	6802090	12727788
OK_BK_RR_rep5	4086989	35.50	203707	106443	888519	6227507	11513165
OK_F1_ABRR_rep1	2827108	36.33	114717	76653	635092	4128959	7782529
OK_F1_ABRR_rep3	4103678	36.64	166263	125584	943686	5860140	11199351
OK_F1_ABRR_rep4	4022270	35.67	169481	114904	899836	6069084	11275575
OK_F1_ABRR_rep5	4800887	34.58	230958	130038	1111926	7611387	13885196
OK_RR_rep1	4267011	37.49	163312	105362	948367	5897108	11381160
OK_RR_rep3	5039456	38.21	194141	125498	1135267	6692961	13187323
OK_RR_rep4	5011368	36.51	227043	135356	1169590	7183406	13726763
OK_RR_rep5	4789517	35.60	218628	128612	1108200	7207882	13452839

File S2: Custom rRNA database details

Folowing this guide https://informatics.fas.harvard.edu/best-practices-for-de-novo-transcriptome-assembly-with-trinity.html, to increase the mRNA transcript yield in the process of transcriptome assembly we mapped trimmed reads against our custom rRNA database consisting of following data:

- RDP database datasets (source: https://rdp.cme.msu.edu/misc/resources.jsp):
  - current_Archaea_unaligned.fa.gz
  - current_Bacteria_unaligned.fa.gz
  - current_Fungi_unaligned.fa.gz
- rrnDB - The ribosomal RNA database dataset (source: https://rrndb.umms.med.umich.edu):
  - rrnDB-5.7_16S_rRNA.fasta
- µgreen database dataset (source: http://microgreen-23sdatabase.ea.inra.fr/):
  - microgreen_ncbi_biocomPipe.fasta
- SILVA rRNA database datasets (source: https://www.arb-silva.de/):
  - SILVA_138.1_LSURef_NR99_tax_silva.fasta.gz
  - SILVA_138.1_SSURef_NR99_tax_silva.fasta.gz

Table S3: The results of the alignment to *Abramis brama* transcriptome

Number of reads per sample categorized right after the alignment. The amount of reads overlapping genes ranges from 43.47% to 62.31%, with 54.95% as the average.

Category	Overlapping Genes	Overlapping Genes (%)	No Feature	Multimapping	Unmapped	Total
K_AB_rep1	7698845	55.03	2825912	1419807	2046601	13991165
K_AB_rep2	6430633	55.24	2267217	1151966	1792428	11642244
K_AB_rep3	7514158	55.59	2575509	1354267	2073364	13517298
K_AB_rep4	7935968	52.10	3170119	1653911	2472119	15232117
K_AB_rep5	6580186	54.86	2408040	1179814	1826290	11994330
K_BK_AB_rep1	7330579	51.53	2812779	1698059	2385224	14226641
K_BK_AB_rep2	7295934	49.92	2898643	1799990	2620448	14615015
K_BK_AB_rep3	5772111	43.47	3008889	2049223	2447959	13278182
K_BK_AB_rep4	6249593	47.04	2796313	1686355	2552150	13284411
K_BK_AB_rep5	7357743	57.40	2212328	1238595	2010467	12819133
K_BK_RR_rep1	8444071	52.15	2995115	1971874	2780129	16191189
K_BK_RR_rep2	6650875	51.52	2378995	1638520	2241583	12909973
K_BK_RR_rep3	7150748	50.79	2598598	1738756	2591763	14079865
K_BK_RR_rep4	6290064	54.21	2163640	1316310	1832869	11602883
K_BK_RR_rep5	7596626	52.00	2699188	1682257	2629950	14608021
K_F1_ABRR_rep1	6413690	54.81	2142563	1308812	1836261	11701326
K_F1_ABRR_rep2	6568258	56.45	1978142	1228977	1860400	11635777
K_F1_ABRR_rep3	6694459	51.79	2468355	1498099	2265676	12926589

K_F1_ABRR_rep4	6997365	45.30	3327524	2063266	3058250	15446405
K_F1_ABRR_rep5	7190246	48.32	3020797	1767874	2900535	14879452
K_RR_rep1	7969434	52.87	2682055	1802724	2618457	15072670
K_RR_rep3	6907461	50.67	2475172	1485144	2764767	13632544
K_RR_rep4	8511770	55.01	2519315	1646630	2795526	15473241
K_RR_rep5	6406952	56.38	1812591	1189643	1954472	11363658
OK_AB_rep1	8145222	58.98	2495840	1274366	1895793	13811221
OK_AB_rep2	7262081	58.83	2229851	1067935	1783606	12343473
OK_AB_rep3	7909319	60.78	2214243	1137938	1751187	13012687
OK_AB_rep4	7663575	60.47	2074946	1060206	1875425	12674152
OK_AB_rep5	7107533	58.13	2250968	1093862	1774056	12226419
OK_BK_AB_rep1	7746022	58.81	2240554	1196200	1988270	13171046
OK_BK_AB_rep2	7067796	57.70	2193082	1155442	1833327	12249647
OK_BK_AB_rep3	7847088	62.31	1949477	1160255	1636045	12592865
OK_BK_AB_rep4	7344288	57.80	2253798	1193225	1914919	12706230
OK_BK_AB_rep5	7135462	57.35	2242858	1183542	1879600	12441462
OK_BK_RR_rep1	7441547	55.44	2297848	1335275	2347142	13421812
OK_BK_RR_rep2	7526842	58.22	2035628	1254039	2111139	12927648
OK_BK_RR_rep3	7008153	55.06	2239854	1243894	2235262	12727163
OK_BK_RR_rep5	6311845	54.82	1900021	1154030	2147288	11513184
OK_F1_ABRR_rep1	4496585	57.78	1321314	744308	1219545	7781752
OK_F1_ABRR_rep3	6392471	57.08	1906191	1141299	1759423	11199384

OK_F1_ABRR_rep4	6474427	57.42	1909622	1060572	1830983	11275604
OK_F1_ABRR_rep5	7747019	55.79	2456054	1309859	2372293	13885225
OK_RR_rep1	6692363	58.81	1667566	1064922	1955259	11380110
OK_RR_rep3	7775682	58.96	1918601	1197384	2295687	13187354
OK_RR_rep4	7750338	56.46	2171433	1328227	2476800	13726798
OK_RR_rep5	7574802	56.31	2102767	1231484	2543815	13452868

Table S4: The results of the alignment to *Rutilus rutilus* transcriptome

Number of reads per sample categorized right after the alignment. The amount of reads overlapping genes ranges from 44.41% to 60.17%, with 54.17% as the average.

Category	Overlapping Genes	Overlapping Genes (%)	No Feature	Multimapping	Unmapped	Total
K_AB_rep1	7533355	53.84	2455337	1233882	2768917	13991491
K_AB_rep2	6341781	54.47	1977549	975298	2347600	11642228
K_AB_rep3	7371584	54.53	2273167	1124804	2747726	13517281
K_AB_rep4	7754712	50.91	2808281	1437048	3232064	15232105
K_AB_rep5	6439631	53.69	2105317	1053368	2396009	11994325
K_BK_AB_rep1	7263712	51.06	2737191	1491080	2734943	14226926
K_BK_AB_rep2	6951901	47.57	2873295	1810317	2979492	14615005
K_BK_AB_rep3	6000170	45.19	2884696	1630412	2762902	13278180
K_BK_AB_rep4	6208921	46.74	2724820	1510705	2839961	13284407
K_BK_AB_rep5	7063542	55.10	2302140	1184592	2268845	12819119
K_BK_RR_rep1	8266014	51.05	3401839	1864335	2658992	16191180
K_BK_RR_rep2	6573257	50.92	2723955	1459552	2153205	12909969
K_BK_RR_rep3	6988573	49.64	3018499	1604264	2468529	14079865
K_BK_RR_rep4	6374527	54.94	2426063	1099467	1703287	11603344
K_BK_RR_rep5	7585586	51.93	2984817	1549998	2487622	14608023
K_F1_ABRR_rep1	6161087	52.65	2379394	1232529	1928559	11701569
K_F1_ABRR_rep2	6344772	54.53	2201673	1157495	1931834	11635774
K_F1_ABRR_rep3	6486246	50.18	2652164	1386323	2401855	12926588

K_F1_ABRR_rep4	6859406	44.41	3456258	1919940	3210796	15446400
K_F1_ABRR_rep5	7031523	47.26	3184675	1684894	2978359	14879451
K_RR_rep1	7897900	52.40	3355974	1729879	2089305	15073058
K_RR_rep3	6967065	51.11	3047409	1371459	2246622	13632555
K_RR_rep4	8310971	53.71	3242792	1644090	2275390	15473243
K_RR_rep5	6365627	56.02	2249111	1139152	1609777	11363667
OK_AB_rep1	7870316	56.99	2248592	1136466	2555832	13811206
OK_AB_rep2	7019299	56.87	1947060	915131	2461964	12343454
OK_AB_rep3	7652611	58.81	2007468	995313	2357271	13012663
OK_AB_rep4	7626614	60.17	1776798	849572	2421343	12674327
OK_AB_rep5	6910112	56.52	1955626	936444	2424213	12226395
OK_BK_AB_rep1	7718136	58.60	2122282	1006025	2324597	13171040
OK_BK_AB_rep2	6956522	56.79	2141237	1014850	2137037	12249646
OK_BK_AB_rep3	7252804	57.59	2183369	1184842	1971845	12592860
OK_BK_AB_rep4	7196065	56.63	2152630	1026939	2330580	12706214
OK_BK_AB_rep5	7047707	56.65	2123409	1052276	2218072	12441464
OK_BK_RR_rep1	7576461	56.45	2514572	1079214	2251568	13421815
OK_BK_RR_rep2	7443413	57.58	2389754	1105788	1988710	12927665
OK_BK_RR_rep3	7120331	55.94	2518963	1049708	2038434	12727436
OK_BK_RR_rep5	6293021	54.66	2305628	979388	1935154	11513191
OK_F1_ABRR_rep1	4363806	56.07	1463118	682450	1272723	7782097
OK_F1_ABRR_rep3	6318359	56.42	2049921	974193	1856914	11199387

OK_F1_ABRR_rep4	6449729	57.20	2007987	865834	1952047	11275597
OK_F1_ABRR_rep5	7810888	56.25	2520625	1051354	2502366	13885233
OK_RR_rep1	6682473	58.72	2140610	959922	1597528	11380533
OK_RR_rep3	7754892	58.81	2451111	1102947	1878413	13187363
OK_RR_rep4	7791207	56.76	2705790	1181214	2048590	13726801
OK_RR_rep5	7718928	57.38	2598267	1062619	2073064	13452878

File S3: The transcriptome analysis details

We produced two transcriptome assemblies for this study, one for *Abramis brama* and the other one for *Rutilus rutilus*. The approach was identical, and the results were very similar.

Transcriptome assembly was carried out by three different tools with multiple k-mer length values (Trinity: 21, 25, 31; rnaSPAdes: 29, 47, 69, 89, 107, 127; MEGAHIT: 21, 29, 39, 59, 79, 99, 119, 141) according to suggestion in http://arthropods.eugenes.org/EvidentialGene/about/EvidentialGene_trassembly_pipe.html that too many transcript assemblies is much better than too few. It allows one then to apply biological criteria to pick out the best ones. Most assembling tools provide the option to set the k-mer length. Some use multiple k-mer values by default (namely MEGAHIT, rnaSPAdes), and some do not (namely Trinity). Moreover, different tools provide different algorithms. Thus, a combination of multiple approaches can provide the best way to utilize their strongest features and produce the most fruitful set of transcripts. All resulting transcripts are then merged together using EvidentialGene's tr2aacds on the basis of CDS-DNA local alignment identity classification. This approach narrows down the number of produced transcripts significantly while keeping the variability of assembled transcripts because the best transcriptome assembly is not about the highest number of the transcripts or longest transcripts but more likely about the highest ratio and completeness of useful information in the transcripts, which in most cases means the protein-coding sequences. The transcriptome assemblies were subjected to various quality control tools: TransRate, rnaQUAST, and BUSCO with its metazoa, vertebrata, and actinopterygian lineages of version odb10. This excludes the various k-mer length-based MEGAHIT assemblies as this tool uses a similar approach on a smaller scale on its own. Next, the annotation step was performed using TransDecoder and Trinotate as primary tools taking advantage of the following tools and databases: UniProtKB/Swiss-Prot database, MEROPS database, RefSeq database, NCBI Nucleotide database in combination with BLAST+; Pfam database in combination with HMMER; and SignalP as a standalone tool. Finally, the abundance of the resulting transcripts was quantified using Salmon. All compatible results and statistics were processed by MultiQC.

As one can see in both transcriptome assemblies results (Table S5-S8), the merged and MEGAHIT assemblies do not have the highest number of transcripts, the highest average length of assembled transcripts, the best N50, nor even the best rate of predicted genes in transcripts (according to the rnaQUAST statistics), however, the other assemblies provide a great portion of duplicity in the transcripts according to the results from BUSCO. That could lead to multi mapping of reads when using transcriptome as a reference. Since the merged assembly achieved the same with lesser transcripts and a better ratio of predicted genes, we decided to use the merged assembly as the main assembly in both cases.

Table S5: rnaQUAST statistics of *R. rutilus* transcriptome assemblies

METRICS/ TRANSCRIPTS	Transcripts	Transcripts > 500 bp	Predicted genes	Average length of assembled transcripts	Longest transcript	Total length	Transcript N50
trinity_21	296745	132332	69781	870.1	24383	258188034	1596
trinity_25	401094	181425	98748	974.3	27554	390794299	2009
trinity_31	406672	192963	109721	1097.7	29300	446415011	2435
spades_29	229111	149311	72793	1261.4	29414	289011564	2096
spades_47	233403	150275	76536	1372.2	24903	320264199	2425
spades_69	205064	123280	67515	1382.6	25469	283522179	2628
spades_89	204093	106516	60819	1205.5	25795	246038857	2467
spades_107	196482	97200	56801	1104.6	25795	217039306	2244
spades_127	182802	86408	53793	1001.8	24853	183122083	1954
megahit	223868	104537	35498	832.3	26698	186324336	1261
merged	182019	62405	33332	692.1	27101	125970660	1152

Table S6: rnaQUAST statistics of *A. brama* transcriptome assemblies

METRICS/ TRANSCRIPTS	Transcripts	Transcripts > 500 bp	Predicted genes	Average length of assembled transcripts	Longest transcript	Total length	Transcript N50
trinity_21	287333	127549	65530	870.7	27565	250177130	1595
trinity_25	360224	157447	84962	962	30511	346549893	2033
trinity_31	358995	165155	92496	1080.2	29985	387788366	2443
spades_29	222190	146018	70044	1275.5	26948	283400283	2126
spades_47	219961	142274	72465	1422	23659	312788151	2562
spades_69	188681	115265	62350	1482.7	20774	279755950	2893
spades_89	184516	99643	55867	1317	29496	243010930	2772
spades_107	174934	90518	51521	1227.9	27173	214805195	2580
spades_127	159414	79606	46847	1120.5	27179	178619966	2299
megahit	192618	98671	33189	929.6	28643	179049008	1502
merged	172630	62277	32380	724.2	28643	125013350	1232

Table S7: BUSCO statistics of *R. rutilus* transcriptome assemblies

Category	Complete and single-copy BUSCOs	Complete and duplicated BUSCOs	Fragmented BUSCOs	Missing BUSCOs
megahit.vertebrata	2932	65	184	173
merged.vertebrata	3047	61	56	190
spades_107.vertebrata	1867	1234	74	179
spades_127.vertebrata	1962	1089	87	216
spades_29.vertebrata	1658	1440	112	144
spades_47.vertebrata	1511	1620	79	144
spades_69.vertebrata	1506	1620	81	147
spades_89.vertebrata	1707	1419	75	153
trinity_21.vertebrata	1516	1543	114	181
trinity_25.vertebrata	1027	2086	82	159
trinity_31.vertebrata	938	2191	67	158
megahit.actinopterygii	3033	59	156	392
merged.actinopterygii	3142	91	49	358
spades_107.actinopterygii	1949	1232	82	377
spades_127.actinopterygii	2010	1082	88	460
spades_29.actinopterygii	1737	1463	101	339
spades_47.actinopterygii	1594	1640	74	332
spades_69.actinopterygii	1596	1636	69	339
spades_89.actinopterygii	1787	1432	76	345
trinity_21.actinopterygii	1609	1559	87	385
trinity_25.actinopterygii	1121	2098	59	362
trinity_31.actinopterygii	1037	2199	52	352
megahit.metazoa	888	37	26	3
merged.metazoa	876	42	11	25
spades_107.metazoa	566	382	3	3
spades_127.metazoa	610	338	4	2
spades_29.metazoa	454	490	8	2
spades_47.metazoa	418	533	1	2
spades_69.metazoa	426	521	5	2
spades_89.metazoa	493	455	4	2
trinity_21.metazoa	473	468	7	6
trinity_25.metazoa	352	592	4	6
trinity_31.metazoa	337	614	1	2

Table S8: BUSCO statistics of *A. brama* transcriptome assemblies

Category	Complete and single-copy BUSCOs	Complete and duplicated BUSCOs	Fragmented BUSCOs	Missing BUSCOs
megahit.actinopterygii	2978	78	197	387
merged.actinopterygii	3140	72	50	378
spades_107.actinopterygii	1973	1217	55	395
spades_127.actinopterygii	2184	946	74	436
spades_29.actinopterygii	1790	1400	102	348
spades_47.actinopterygii	1657	1563	77	343
spades_69.actinopterygii	1698	1534	60	348
spades_89.actinopterygii	1841	1375	60	364
trinity_21.actinopterygii	1678	1464	88	410
trinity_25.actinopterygii	1340	1859	53	388
trinity_31.actinopterygii	1271	1939	42	388
megahit.vertebrata	2898	65	216	175
merged.vertebrata	3043	54	62	195
spades_107.vertebrata	1838	1255	72	189
spades_127.vertebrata	2078	988	66	222
spades_29.vertebrata	1679	1413	104	158
spades_47.vertebrata	1543	1578	79	154
spades_69.vertebrata	1572	1551	73	158
spades_89.vertebrata	1703	1396	85	170
trinity_21.vertebrata	1581	1464	117	192
trinity_25.vertebrata	1243	1856	70	185
trinity_31.vertebrata	1150	1961	68	175
megahit.metazoa	889	37	25	3
merged.metazoa	894	34	9	17
spades_107.metazoa	556	393	3	2
spades_127.metazoa	650	300	2	2
spades_29.metazoa	474	473	4	3
spades_47.metazoa	411	538	3	2
spades_69.metazoa	451	498	1	4
spades_89.metazoa	496	453	3	2
trinity_21.metazoa	519	422	9	4
trinity_25.metazoa	417	531	1	5
trinity_31.metazoa	391	560	0	3

Script S1: GO enrichment analysis results postprocessing code in R language

```
library("data.table")
library("clusterProfiler")
library("ontologyIndex")
library("splitstackshape")
library("pheatmap")
library("ggplot2")

wdir = "/mnt/ssd/ssd_1/workspace/martin/abramis_rutilus_DE"
go_dir = "/mnt/nfs/shared/CFBioinformatics/references_backup/general/GO/"
go_tab = fread(file =
"/mnt/nfs/shared/CFBioinformatics/references_backup/rutilus_rutilus/rutilus_rutilus_TA/annot/g
o_annotations.txt", header = F, fill = T, sep = '\t', col.names = c("gene_ID","GO_ID"))
go_tab = cSplit(go_tab, "GO_ID", sep=",", direction = "long")
go_db  <- get_ontology(paste0(go_dir,"go-basic.obo"), extract_tags = "everything")
go_tab <- data.table(GO_ID=go_db$id, GO_Name=go_db$name, GO_Category=unlist(go_db$namespace),
GO_Obsolete=go_db$obsolete)[go_tab, on="GO_ID"]
rm(go_db)

FC = 2 # FC cutoff
FC = log(FC, 2) # Get log2FC
FDR = 0.05 # Adj. p-val. cutoff
top_class = 10

inpdir = "/mnt/ssd/ssd_1/workspace/martin/abramis_rutilus_DE/mRNA_DE_featureCount"
conds = dir(inpdir, pattern = "_vs_")
for(cond in conds) {
  DE = "DESeq2.tsv"
  REGUL = "up"
  for(REGUL in c("up", "down", "both")){
        print(paste0("Working with ", REGUL, "-regulated genes of condition ",cond))

        outdir <-
paste0(wdir,"/GO_enrichment.using_data_from_trinotate/",cond,"/FDR_",FDR,".l2FC_",FC,"/",REGUL
)
        dir.create(outdir, recursive = T)
        setwd(outdir)

        de_tab = fread(paste0(inpdir, "/", cond,"/all/",DE), header = T, sep = "\t", select =
1:9)
        setnames(de_tab,"V1","gene_id")
        de_tab = de_tab[go_tab, on="gene_id==gene_ID"]

        if(REGUL == "up"){
        signif = de_tab[!is.na(GO_ID) & GO_ID != "" & GO_Obsolete == FALSE & !is.na(GO_Name) &
!is.na(GO_Category) & !is.na(padj) & padj <= FDR & log2FoldChange >= FC,]
        }else if(REGUL == "down"){
        signif = de_tab[!is.na(GO_ID) & GO_ID != "" & GO_Obsolete == FALSE & !is.na(GO_Name) &
!is.na(GO_Category) & !is.na(padj) & padj <= FDR & -log2FoldChange >= FC,]
        }else if(REGUL == "both"){
        signif = de_tab[!is.na(GO_ID) & GO_ID != "" & GO_Obsolete == FALSE & !is.na(GO_Name) &
!is.na(GO_Category) & !is.na(padj) & padj <= FDR & abs(log2FoldChange) >= FC,]
        }else{
        "Unknown expression change."
        }

        if(signif[, .N] != 0) {
        res <- enricher(gene = signif$gene_id,
                        TERM2GENE =
de_tab[!is.na(GO_ID),.N,by=.(GO_ID,gene_id)][,.(term=GO_ID,gene=gene_id)],
                        TERM2NAME =
de_tab[!is.na(GO_ID),.N,by=.(GO_ID,GO_Name)][,.(term=GO_ID,name=GO_Name)],
                        minGSSize      = 2,
                        maxGSSize      = 30000,
                        pAdjustMethod = "BH",
```

```
                        pvalueCutoff  = 1,
                        qvalueCutoff  = 1)
        res@result <- unique(go_tab[,.(GO_ID, GO_Category)][data.table(res@result),
on="GO_ID==ID"])
        fwrite(as.data.table(res@result), "enricher_GO.tsv", sep = "\t", quote = F, row.names
= F, col.names = T)

        wtab =
res@result[GO_Category!="",.SD[order(qvalue)][1:top_class,.SD,.SDcols=patterns("Ratio|^GO_|Des
cription")],.(GO_Category)]
        setnames(wtab,c("Description","GeneRatio","BgRatio"),
c("GO_Description","DE_genes","All_genes"))
        wtab = melt.data.table(wtab, measure.vars = patterns("_genes"))
        wtab[,fvalue:=sapply(value, function(x) as.double(eval(parse(text=x))))]
        plot_name_prefix =
paste0("GO_enrich.",cond,".FDR_",FDR,".l2FC_",FC,".",REGUL,".top_",top_class,"_by_groups")
        # horizontal
        hplot = ggplot(wtab, aes(x=paste0(GO_Description,"\n",GO_ID), y=fvalue)) +
                geom_bar(aes(fill=variable), stat="identity", position=position_dodge()) +
                geom_text(aes(label=value, color=variable), hjust=0.5, vjust=-0.3, size=2.8)+
                facet_wrap(~ GO_Category, scales = "free_x") +
                theme(axis.text.x = element_text(angle = 60, hjust = 1)) +
                scale_y_continuous(labels = percent, breaks = scales::extended_breaks(n=10)) +
                labs(x = "GO terms classification",
                        y = "Enrichment ratios (%)",
                        title = paste0("GO enrichment analysis for comparison ",cond),
                        subtitle = paste0("DE genes filtered by FDR <= ",FDR," and
",ifelse(REGUL=="both","abs(log2FC) >= ",paste0("log2FC ",ifelse(REGUL=="up",">= ","<= -
"))),FC),
                        fill = "Gene set",
                        color = "Gene set")
        ggsave(paste0(plot_name_prefix,".horz.png"), hplot, width = 16, height = 9, units =
"in")
        ggsave(paste0(plot_name_prefix,".horz.pdf"), hplot, width = 16, height = 9, units =
"in")
        # vertical
        vplot = ggplot(wtab, aes(y=paste0(GO_Description,"\n",GO_ID), x=fvalue)) +
                geom_bar(aes(fill=variable), stat="identity", position=position_dodge()) +
                geom_text(aes(label=value, color=variable,
vjust=fifelse(variable=="DE_genes",1.2,-0.2)), hjust=-0.1, size=3.5)+
                facet_wrap(~ GO_Category, scales = "free_y", ncol = 1) +
                theme(axis.text.y = element_text(size = 12),
                        axis.text.x = element_text(size = 12)) +
                scale_x_continuous(labels = percent, breaks = scales::extended_breaks(n=10)) +
                labs(y = "GO terms classification",
                        x = "Enrichment ratios (%)",
                        title = paste0("GO enrichment analysis for comparison ",cond),
                        subtitle = paste0("DE genes filtered by FDR <= ",FDR," and
",ifelse(REGUL=="both","abs(log2FC) >= ",paste0("log2FC ",ifelse(REGUL=="up",">= ","<= -
"))),FC),
                        fill = "Gene set",
                        color = "Gene set")
        ggsave(paste0(plot_name_prefix,".vert.png"), vplot, width = 16, height = 16, units =
"in")
        ggsave(paste0(plot_name_prefix,".vert.pdf"), vplot, width = 16, height = 16, units =
"in")
        }
  }
}

##############################
library(pheatmap)
library(openxlsx)

wdir = "/mnt/ssd/ssd_1/workspace/martin/abramis_rutilus_DE/"

FC = 2 # FC cutoff
```

```
FC = log(FC, 2) # Get log2FC
FDR = 0.05 # Adj. p-val. cutoff
max_rows = 100
breaksList = seq(0, 1, by = 0.1)

treatment = c("OK", "PH")
control  = c("K")
lineages = c("RR","AB","BK_AB","BK_RR","F1_ABRR","F1_RRAB")
directions = c("up","down")

dir  = directions[1]
ctrl = control[1]
trt  = treatment[1]
lin  = lineages[1]
for(dir in directions) {
  for(ctrl in control) {
        for(trt in treatment) {
        conds = c()
        for(lin in lineages) {
        cond = paste(trt,lin,"vs",ctrl,lin,sep = "_")
        inp =
paste0(wdir,"/GO_enrichment.using_data_from_trinotate/",cond,"/FDR_",FDR,".l2FC_",FC,"/",dir,"
/enricher_GO.tsv")
        if(file.exists(inp)) conds = append(conds, cond)
        }
        tab = Reduce(function(...) {merge(..., by = c("GO_ID","GO_Category","GO_Description"),
all = T)},
                       lapply(conds, function(cond) {

        fread(paste0(wdir,"/GO_enrichment.using_data_from_trinotate/",cond,"/FDR_",FDR,".l2FC_
",FC,"/",dir,"/enricher_GO.tsv"),
                               sep = "\t",
                               select =
c("GO_ID","GO_Category","Description","GeneRatio","BgRatio","qvalue","geneID","Count"),
                               col.names =
c("GO_ID","GO_Category","GO_Description",paste0(cond,".DEG_ratio"),
                                       paste0(cond,".all_genes_ratio"),paste0(cond,".q-
value"),paste0(cond,".gene_IDs"),paste0(cond,".gene_count")))
                       })
        )
        fwrite(tab[,.SD,.SDcols=patterns("gene_IDs|^GO_")],

        paste0(wdir,"/GO_enrichment.using_data_from_trinotate/GO_terms.",trt,"_vs_",ctrl,".FDR
_",FDR,".l2FC_",FC,".",dir,"_reg.full.tsv"),
               sep = '\t',
               row.names = F,
               col.names = T,
               quote = F)
        tab_copy = tab[,.SD,.SDcols=patterns("q-value")]
        tab_copy[is.na(tab_copy)] = 1
        tab_copy[,sum:=rowSums(.SD)]
        tab[,sum:=tab_copy$sum]
        tab_copy = tab[order(sum)]
        fwrite(tab_copy[,.SD,.SDcols=patterns("q-value|^GO_")],

        paste0(wdir,"/GO_enrichment.using_data_from_trinotate/GO_heatmap.",trt,"_vs_",ctrl,".F
DR_",FDR,".l2FC_",FC,".",dir,"_reg.full.tsv"),
               sep = '\t',
               row.names = F,
               col.names = T,
               quote = F)
        hm = pheatmap(tab_copy[1:max_rows,.SD,.SDcols=patterns("q-value")],
                       main = paste0("GO enrichment for ",trt,"_vs_",ctrl," (top
",max_rows,")\n[FDR <= ",FDR," and log2FC ",ifelse(dir=="up",">= ","<= -"),FC,"]"),
                       cluster_rows = F,
                       cluster_cols = T,
                       na_col = "black",
```

```
                      angle_col = 315,
                      color = colorRampPalette(c("mediumblue", "white"))(length(breaksList)),
                      breaks = breaksList,
                      labels_row = tab_copy[1:max_rows,paste0(GO_ID,": ",GO_Description)])

       pdf(paste0(wdir,"/GO_enrichment.using_data_from_trinotate/GO_heatmap.",trt,"_vs_",ctrl
,".FDR_",FDR,".l2FC_",FC,".",dir,"_reg.top_",max_rows,".pdf"),
       width = 9,
       height = 16,
       pointsize = 12)
       print(hm)
       dev.off()
       }
  }
}

##############################
library(data.table)
library(ggplot2)

wdir = "/mnt/ssd/ssd_1/workspace/martin/abramis_rutilus_DE/"

FC = 2 # FC cutoff
FC = log(FC, 2) # Get log2FC
FDR = 0.05 # Adj. p-val. cutoff
max_rows = 10
breaksList = seq(0, 1, by = 0.1)

treatment = c("OK", "PH")
control  = c("K")
lineages = c("RR","AB","BK_AB","BK_RR","F1_ABRR","F1_RRAB")
directions = c("up","down", "both")

dir  = directions[1]
ctrl = control[1]
trt  = treatment[1]
lin  = lineages[1]
for(dir in directions) {
  for(ctrl in control) {
       for(trt in treatment) {
       conds = c()
       for(lin in lineages) {
       cond = paste(trt,lin,"vs",ctrl,lin,sep = "_")
       inp =
paste0(wdir,"/GO_enrichment.using_data_from_trinotate/",cond,"/FDR_",FDR,".l2FC_",FC,"/",dir,"
/enricher_GO.tsv")
       if(file.exists(inp)) conds = append(conds, cond)
       }
       tab = Reduce(function(...) {merge(..., by = c("GO_ID","GO_Category","GO_Description"),
all = T)},
                      lapply(conds, function(cond) {
                      tt =
fread(paste0(wdir,"/GO_enrichment.using_data_from_trinotate/",cond,"/FDR_",FDR,".l2FC_",FC,"/"
,dir,"/enricher_GO.tsv"),
                              sep = "\t",
                              select =
c("GO_ID","GO_Category","Description","GeneRatio","BgRatio","qvalue","geneID","Count"))
                      tt[,DEG_ratio:=sapply(GeneRatio, function(x)
as.double(eval(parse(text=x))))]
                      tt[,all_genes_ratio:=sapply(BgRatio, function(x)
as.double(eval(parse(text=x))))]
                      setnames(tt,
c("GO_ID","GO_Category","Description","GeneRatio","BgRatio","DEG_ratio","all_genes_ratio","qva
lue","geneID","Count"),

       c("GO_ID","GO_Category","GO_Description",paste0(cond,".GeneRatio"),paste0(cond,".BgRat
io"),paste0(cond,".DEG_ratio"),
```

```
                              paste0(cond,".all_genes_ratio"),paste0(cond,".q-
value"),paste0(cond,".gene_IDs"),paste0(cond,".gene_count")))
                    return(tt)
                    })
        )
        tab_copy = tab[,.SD,.SDcols=patterns("q-value")]
        tab_copy[is.na(tab_copy)] = 1
        tab_copy[,sum:=rowSums(.SD)]
        tab[,sum:=tab_copy$sum]
        tab[,show:=GO_ID %in% tab[,.SD[order(sum),GO_ID][1:max_rows],GO_Category]$V1]
        ltab = melt.data.table(tab, id.vars = c("GO_ID", "GO_Category", "GO_Description",
"sum", "show"),
                              variable.name = "lineage",
                              measure.vars =
patterns(GeneRatio=".GeneRatio$",DEG_ratio=".DEG_ratio$",BgRatio=".BgRatio$",all_genes_ratio="
.all_genes_ratio$",
                                                                    qvalue=".q-
value$",gene_IDs=".gene_IDs$",gene_count=".gene_count$"))
        ltab[,lineage:=conds[lineage]]
        ltab[,enrichment:=DEG_ratio/all_genes_ratio]
        plot_name_prefix =
paste0(wdir,"/GO_enrichment.using_data_from_trinotate/GO_enrich_summary.",trt,"_vs_",ctrl,".FD
R_",FDR,".l2FC_",FC,".",dir,".top_",max_rows,"_by_groups")
        ggplot(ltab[GO_Category!="" & show], aes(x=enrichment,
y=paste0(GO_Description,"\n",GO_ID))) +
        geom_point(aes(color=-log2(qvalue), size=gene_count)) +
        # lims(y =
unique(ltab[GO_Category!="",.(GO_ID,GO_Category,GO_Description,sum)])[,.SD[order(sum),paste0(G
O_Description,"\n",GO_ID)][1:10],.(GO_Category)]$V1) +
        facet_grid(vars(GO_Category), vars(lineage), scales = "free") +
        scale_x_continuous(breaks = scales::extended_breaks(n=8)) +
        labs(y = "GO terms classification",
                x = "Enrichment",
                title = paste0("GO enrichment analysis summary"),
                subtitle = paste0("DE genes filtered by FDR <= ",FDR," and
",ifelse(dir=="both","abs(log2FC) >= ",paste0("log2FC ",ifelse(dir=="up",">= ","<= -"))),FC),
                size = "DEGs count",
                color = "S-value")
        ggsave(paste0(plot_name_prefix,".png"), width = 16, height = 12, units = "in")
        ggsave(paste0(plot_name_prefix,".pdf"), width = 16, height = 12, units = "in")
        }
  }
}

##############################
library(data.table)
library(ggplot2)
library(ggnewscale)

wdir = "/mnt/ssd/ssd_1/workspace/martin/abramis_rutilus_DE/"

FC = 2 # FC cutoff
FC = log(FC, 2) # Get log2FC
FDR = 0.05 # Adj. p-val. cutoff
max_rows = 10
breaksList = seq(0, 1, by = 0.1)

treatment = c("OK", "PH")
control  = c("K")
lineages = c("RR","AB","BK_AB","BK_RR","F1_ABRR","F1_RRAB")
directions = c("up","down")

selected_GOs =
c("GO:0030218","GO:0020037","GO:0008009","GO:0006955","GO:0050896","GO:0005839","GO:0051603","
GO:0004867","GO:0071353","GO:0045070","GO:0005080","GO:0005509","GO:0005515","GO:0005178","GO:
0045638","GO:0004252","GO:0001946")
```

```
ctrl = control[1]
trt  = treatment[1]
lin  = lineages[1]
for(ctrl in control) {
  for(trt in treatment) {
        # up-regulated genes
        conds = c()
        dir = "up"
        for(lin in lineages) {
        cond = paste(trt,lin,"vs",ctrl,lin,sep = "_")
        inp =
paste0(wdir,"/GO_enrichment.using_data_from_trinotate/",cond,"/FDR_",FDR,".l2FC_",FC,"/",dir,"
/enricher_GO.tsv")
        if(file.exists(inp)) conds = append(conds, cond)
        }
        tab = Reduce(function(...) {merge(..., by = c("GO_ID","GO_Category","GO_Description"),
all = T)},
                lapply(conds, function(cond) {
                        tt =
fread(paste0(wdir,"/GO_enrichment.using_data_from_trinotate/",cond,"/FDR_",FDR,".l2FC_",FC,"/"
,dir,"/enricher_GO.tsv"),
                                sep = "\t",
                                select =
c("GO_ID","GO_Category","Description","GeneRatio","BgRatio","qvalue","geneID","Count"))
                        tt[,DEG_ratio:=sapply(GeneRatio, function(x)
as.double(eval(parse(text=x))))]
                        tt[,all_genes_ratio:=sapply(BgRatio, function(x)
as.double(eval(parse(text=x))))]
                        setnames(tt,
c("GO_ID","GO_Category","Description","GeneRatio","BgRatio","DEG_ratio","all_genes_ratio","qva
lue","geneID","Count"),

        c("GO_ID","GO_Category","GO_Description",paste0(cond,".GeneRatio"),paste0(cond,".BgRat
io"),paste0(cond,".DEG_ratio"),
                                paste0(cond,".all_genes_ratio"),paste0(cond,".q-
value"),paste0(cond,".gene_IDs"),paste0(cond,".gene_count")))
                        return(tt)
                })
        )
        tab[,show:=GO_ID %in% selected_GOs]
        utab = melt.data.table(tab, id.vars = c("GO_ID", "GO_Category", "GO_Description",
"show"),
                                variable.name = "lineage",
                                measure.vars =
patterns(GeneRatio=".GeneRatio$",DEG_ratio=".DEG_ratio$",BgRatio=".BgRatio$",all_genes_ratio="
.all_genes_ratio$",
                                                qvalue=".q-
value$",gene_IDs=".gene_IDs$",gene_count=".gene_count$"))
        utab[,lineage:=conds[lineage]]
        utab[,enrichment:=DEG_ratio/all_genes_ratio]
        utab[,direction:=dir]
        # down-regulated genes
        conds = c()
        dir = "down"
        for(lin in lineages) {
        cond = paste(trt,lin,"vs",ctrl,lin,sep = "_")
        inp =
paste0(wdir,"/GO_enrichment.using_data_from_trinotate/",cond,"/FDR_",FDR,".l2FC_",FC,"/",dir,"
/enricher_GO.tsv")
        if(file.exists(inp)) conds = append(conds, cond)
        }
        tab = Reduce(function(...) {merge(..., by = c("GO_ID","GO_Category","GO_Description"),
all = T)},
                lapply(conds, function(cond) {
                        tt =
fread(paste0(wdir,"/GO_enrichment.using_data_from_trinotate/",cond,"/FDR_",FDR,".l2FC_",FC,"/"
,dir,"/enricher_GO.tsv"),
```

```
                              sep = "\t",
                              select =
c("GO_ID","GO_Category","Description","GeneRatio","BgRatio","qvalue","geneID","Count"))
                    tt[,DEG_ratio:=sapply(GeneRatio, function(x)
as.double(eval(parse(text=x))))]
                    tt[,all_genes_ratio:=sapply(BgRatio, function(x)
as.double(eval(parse(text=x))))]
                    setnames(tt,
c("GO_ID","GO_Category","Description","GeneRatio","BgRatio","DEG_ratio","all_genes_ratio","qva
lue","geneID","Count"),

        c("GO_ID","GO_Category","GO_Description",paste0(cond,".GeneRatio"),paste0(cond,".BgRat
io"),paste0(cond,".DEG_ratio"),
                              paste0(cond,".all_genes_ratio"),paste0(cond,".q-
value"),paste0(cond,".gene_IDs"),paste0(cond,".gene_count")))
                    return(tt)
               })
        )
        tab[,show:=GO_ID %in% selected_GOs]
        dtab = melt.data.table(tab, id.vars = c("GO_ID", "GO_Category", "GO_Description",
"show"),
                              variable.name = "lineage",
                              measure.vars =
patterns(GeneRatio=".GeneRatio$",DEG_ratio=".DEG_ratio$",BgRatio=".BgRatio$",all_genes_ratio="
.all_genes_ratio$",
                                                     qvalue=".q-
value$",gene_IDs=".gene_IDs$",gene_count=".gene_count$"))
        dtab[,lineage:=conds[lineage]]
        dtab[,enrichment:=DEG_ratio/all_genes_ratio]
        dtab[,direction:=dir]

        ltab = rbind(utab, dtab)

        plot_name_prefix =
paste0(wdir,"/GO_enrichment.using_data_from_trinotate/GO_enrich_selection.",trt,"_vs_",ctrl,".
FDR_",FDR,".l2FC_",FC,".top_",max_rows,"_by_groups")
        ggplot(ltab[show==T], aes(x=enrichment, y=paste0(GO_Description,"\n",GO_ID))) +
        geom_point(aes(color=-log2(qvalue), size=gene_count), ltab[show==T & direction=="up"])
+
        scale_color_gradient(name = "S-value (Up)", low = "lightskyblue", high = "green3") +
        new_scale_color() +
        geom_point(aes(color=-log2(qvalue), size=gene_count), ltab[show==T &
direction=="down"]) +
        scale_color_gradient(name = "S-value (Down)", low = "orange", high = "red3") +
        #facet_grid(vars(GO_Category), vars(lineage), scales = "free") +
        facet_wrap(~lineage, nrow = 1, scales = "free_x") +
        scale_x_continuous(breaks = scales::extended_breaks(n=8)) +
        labs(y = "GO terms classification",
        x = "Enrichment",
        title = paste0("GO enrichment analysis for selection"),
        subtitle = paste0("DE genes filtered by FDR <= ",FDR," and abs(log2FC) >= ",FC),
        size = "DEGs count") +
        theme_bw()
        ggsave(paste0(plot_name_prefix,".png"), width = 16, height = 12, units = "in")
        ggsave(paste0(plot_name_prefix,".pdf"), width = 16, height = 12, units = "in")
  }
}
```

Script S2: KEGG enrichment analysis results postprocessing code in R language

```
library("data.table")
library("pathview")
library("clusterProfiler")

wdir = "/mnt/ssd/ssd_1/workspace/martin/abramis_rutilus_DE"
ko_sufix = ".using_DRE_CCAR"
ko_tab = fread(file =
paste0("/mnt/nfs/shared/CFBioinformatics/references_backup/rutilus_rutilus/rutilus_rutilus_TA/
annot/longest_orfs.transcripts_to_KEGG",ko_sufix,".tsv"), header = F, fill = T, sep = '\t',
col.names = c("transcript_ID","KO_ID"))
tr2gene = fread(file =
"/mnt/nfs/shared/CFBioinformatics/references_backup/rutilus_rutilus/rutilus_rutilus_TA/annot/t
ranscript2gene.map", header = F, sep = '\t', col.names = c("genes","transcripts"))
ko_tab = ko_tab[tr2gene, on="transcript_ID==transcripts"]

FC = 2 # FC cutoff
FC = log(FC, 2) # Get log2FC
FDR = 0.05 # Adj. p-val. cutoff

inpdir = "/mnt/ssd/ssd_1/workspace/martin/abramis_rutilus_DE/mRNA_DE_featureCount"
conds = dir(inpdir, pattern = "_vs_")
for(cond in conds) {
  DE = "DESeq2.tsv"
  for(REGUL in c("up", "down", "both")){
	print(paste0("Working with ", REGUL, "-regulated genes of condition ",cond))

	outdir <-
paste0(wdir,"/KEGG_enrichment.using_KAAS",ko_sufix,"/",cond,"/FDR_",FDR,".l2FC_",FC,"/",REGUL)
	dir.create(outdir, recursive = T)
	setwd(outdir)

	de_tab = fread(paste0(inpdir, "/", cond,"/all/",DE), header = T, sep = "\t", select =
1:9)
	setnames(de_tab,"V1","gene_id")
	de_tab = de_tab[ko_tab, on="gene_id==genes"]

	if(REGUL == "up"){
	signif = de_tab[!is.na(KO_ID) & KO_ID != "" & !is.na(padj) & padj <= FDR &
log2FoldChange >= FC,
	                .(gene_id, KO_ID, log2FoldChange)]
	}else if(REGUL == "down"){
	signif = de_tab[!is.na(KO_ID) & KO_ID != "" & !is.na(padj) & padj <= FDR & -
log2FoldChange >= FC,
	                .(gene_id, KO_ID, log2FoldChange)]
	}else if(REGUL == "both"){
	signif = de_tab[!is.na(KO_ID) & KO_ID != "" & !is.na(padj) & padj <= FDR &
abs(log2FoldChange) >= FC,
	                .(gene_id, KO_ID, log2FoldChange)]
	}else{
	"Unknown expression change."
	}

	kk = enrichKEGG(gene = unique(as.character(signif$KO_ID)),
	                organism      = 'ko',
	                pvalueCutoff  = 1,
	                qvalueCutoff  = 1,
	                minGSSize     = 2,
	                maxGSSize     = 30000,
	                pAdjustMethod = "BH",
	                universe      = unique(as.character(de_tab[!is.na(KO_ID) & KO_ID !=
"", KO_ID])))
	fwrite(as.data.table(kk@result), "enrichKEGG.tsv", sep = "\t", quote = F, row.names =
F, col.names = T)
```

```
        kegg_gene_list = signif$log2FoldChange
        names(kegg_gene_list) = signif$KO_ID
        kegg_gene_list = na.omit(kegg_gene_list)
        kegg_gene_list = sort(kegg_gene_list, decreasing = TRUE)

        if(as.data.table(kk@result)[,.N] > 0){
        keggname = as.data.table(kk@result)[1,ID]
        keggname = as.data.table(kk@result)[qvalue <= 0.05 & !gsub('^map','',ID) %in%
c("01100","01110"), ID]
        pathview(gene.data  = kegg_gene_list,
                pathway.id = gsub('^map','',keggname),
                species = "ko",
                kegg.native = FALSE,
                limit   = list(gene=max(abs(signif$log2FoldChange)), cpd=1))
        }
        #browseKEGG(kk, rownames(kk@result)[3])
  }
}

##############################
library(pheatmap)
library(openxlsx)

wdir = "/mnt/ssd/ssd_1/workspace/martin/abramis_rutilus_DE/"

FC = 2 # FC cutoff
FC = log(FC, 2) # Get log2FC
FDR = 0.05 # Adj. p-val. cutoff
max_rows = 30
breaksList = seq(0, 1, by = 0.1)

treatment = c("OK", "PH")
control  = c("K")
lineages = c("RR","AB","BK_AB","BK_RR","F1_ABRR","F1_RRAB")
directions = c("up","down")

dir  = directions[1]
ctrl = control[1]
trt  = treatment[1]
lin  = lineages[1]
for(dir in directions) {
  for(ctrl in control) {
        for(trt in treatment) {
        conds = c()
        for(lin in lineages) {
        cond = paste(trt,lin,"vs",ctrl,lin,sep = "_")
        inp =
paste0(wdir,"/KEGG_enrichment.using_KAAS",ko_sufix,"/",cond,"/FDR_",FDR,".l2FC_",FC,"/",dir,"/
enrichKEGG.tsv")
        if(file.exists(inp)) conds = append(conds, cond)
        }
        tab = Reduce(function(...) {merge(..., by = c("ID","Description"), all = T)},
        lapply(conds, function(cond) {

        fread(paste0(wdir,"/KEGG_enrichment.using_KAAS",ko_sufix,"/",cond,"/FDR_",FDR,".l2FC_"
,FC,"/",dir,"/enrichKEGG.tsv"),
                sep = "\t",
                select = c("ID","Description","qvalue","geneID"),
                col.names =
c("ID","Description",paste0(cond,".qvalue"),paste0(cond,".geneIDs")))
        })
        )
        fwrite(tab[,.SD,.SDcols=!patterns("qvalue")],

        paste0(wdir,"/KEGG_enrichment.using_KAAS",ko_sufix,"/KEGG_terms_from_KAAS.",trt,"_vs_"
,ctrl,".FDR_",FDR,".l2FC_",FC,".",dir,"_reg.full.tsv"),
                sep = '\t',
```

```
                row.names = F,
                col.names = T,
                quote = F)
        tab_copy = tab[,.SD,.SDcols=patterns("qvalue")]
        tab_copy[is.na(tab_copy)] = 1
        tab_copy[,sum:=rowSums(.SD)]
        tab[,sum:=tab_copy$sum]
        tab_copy = tab[order(sum)]
        fwrite(tab_copy[,.SD,.SDcols=!patterns("geneIDs")],

        paste0(wdir,"/KEGG_enrichment.using_KAAS",ko_sufix,"/heatmap_from_KAAS.",trt,"_vs_",ct
rl,".FDR_",FDR,".l2FC_",FC,".",dir,"_reg.full.tsv"),
                sep = '\t',
                row.names = F,
                col.names = T,
                quote = F)
        tab_copy = tab_copy[1:max_rows]
        hm = pheatmap(tab_copy[,.SD,.SDcols=patterns("qvalue")],
                        main = paste0("KEGG pathways enrichment for ",trt,"_vs_",ctrl," (top
",max_rows,")\n[FDR <= ",FDR," and log2FC ",ifelse(dir=="up",">= ","<= -"),FC,"]\nblack means
missing values"),
                        cluster_rows = F,
                        cluster_cols = F,
                        na_col = "black",
                        color = colorRampPalette(c("mediumblue", "white"))(length(breaksList)),
                        breaks = breaksList,
                        labels_row = tab_copy[,paste0(ID,": ",Description)])

        pdf(paste0(wdir,"/KEGG_enrichment.using_KAAS",ko_sufix,"/heatmap_from_KAAS.",trt,"_vs_
",ctrl,".FDR_",FDR,".l2FC_",FC,".",dir,"_reg.top_",max_rows,".pdf"),
        width = 16,
        height = 9,
        pointsize = 12)
        print(hm)
        dev.off()
        }
  }
}
```
